# Correction: Prevention and management of type 2 diabetes mellitus in Uganda and South Africa: Findings from the SMART2D pragmatic implementation trial

**DOI:** 10.1371/journal.pgph.0003395

**Published:** 2024-06-14

**Authors:** David Guwatudde, Peter Delobelle, Pilvikki Absetz, Josefien van Olmen, Roy William Mayega, Francis Xavier Kasujja, Jeroen De Man, Mariam Hassen, Elizabeth Ekirapa Kiracho, Juliet Kiguli, Thandi Puoane, Claes-Goran Ostenson, Stefan Peterson, Meena Daivadanam, SMART2D Consortium

The fourth author’s name is written incorrectly. The correct name is: Josefien van Olmen. The correct citation is: Guwatudde D, Delobelle P, Absetz P, van Olmen J, Mayega RW, Kasujja FX, et al. (2022) Prevention and management of type 2 diabetes mellitus in Uganda and South Africa: Findings from the SMART2D pragmatic implementation trial. PLOS Glob Public Health 2(5): e0000425. https://doi.org/10.1371/journal.pgph.0000425

## References

[1] Guwatudde D, Delobelle P, Absetz P, Van JO, Mayega RW, Kasujja FX, et al. (2022) Prevention and management of type 2 diabetes mellitus in Uganda and South Africa: Findings from the SMART2D pragmatic implementation trial. PLOS Glob Public Health 2(5): e0000425. 10.1371/journal.pgph.0000425 36962331 PMC10021626

