# Supplementary material for: Prevention and management of type 2 diabetes mellitus in Uganda and South Africa: Findings from the SMART2D pragmatic implementation trial
Source: PLOS Glob Public Health. 2022 May 2;2(5):e0000425. doi: 10.1371/journal.pgph.0000425 (PMC10021626; doi:10.1371/journal.pgph.0000425)
Supplement: S1 Table — (DOCX) [file pgph.0000425.s001.docx]

**S1 Table: Effectiveness of interventions on glycaemic control, and on reduction in HbA1c of at least 3 mmol/mol (Using intention-to-treat analysis strategy)**

| **Country** | **Study arm** | **Glycaemic control among participants with T2D** | | | | **Reduction in HbA1c of at least 3 mmol/mol among participants at high risk of T2D** | | | |
| --- | --- | --- | --- | --- | --- | --- | --- | --- | --- |
|  |  | **number**  **enrolled** | **Number with glycemic control (%)** | **Crude IRR [95% CI]** | **Adjusted IRR [95% CI]** | **number**  **enrolled** | **Number with reduction in HbA1c > 3 mmol/mol (%)** | **Crude IRR [95% CI]** | **Adjusted IRR [95% CI]** |
|  | Usual care | 141 | 46 (32.6%) | 1.0 | 1.0 | 125 | 22 (17.6%) | 1.0 | 1.0 |
| Uganda | Facility care | 141 | 42 (29.8%) | 0.91 [0.65 – 1.29] | 0.96 [0.73 – 1.26]**^a^** | 126 | 47 (37.3%) | 2.12 [1.36 – 3.30] | 1.94 [1.01 –3.74]**^d^** |
|  | Integrated care | 142 | 36 (25.4%) | 0.78 [0.54 – 1.12] | 0.82 [0.53 – 1.25]**^a^** | 126 | 69 (54.8%) | 3.11 [2.06 – 4.70] | 3.01 [1.67 – 5.40]**^d^** |
|  | sub-total | 424 | 124 (29.2%) |  |  | 377 | 138 (36.6%) |  |  |
|  |  |  |  |  |  |  |  |  |  |
|  | ICC = 0.00128^¥^ (SE = 0.01133) | | |  |  | ICC = 0.15334^±^ (SE= 0.07427) | | |  |
|  |  |  |  |  |  |  |  |  |  |
| South Africa | Facility care | 141 | 25 (17.7%) | 1.0 | 1.0 | 140 | 9 (6.4%) | 1.0 | 1.0 |
|  | Integrated care | 140 | 21 (15.0%) | 0.89 [0.52 – 1.50] | 1.00 [0.99 – 1.00]**^b^** | 145 | 7 (4.8%) | 0.75 [0.29 – 1.96] | 0.57 [0.41 – 0.79]**^e^** |
|  | sub-total | 281 | 46 (16.4%) |  |  | 285 | 16 (5.6%) |  |  |
|  |  |  |  |  |  |  |  |  |  |
|  | ICC < 00001^§^ (SE= 0.01008) | | |  |  | ICC< 0.0001^€^ (SE = 0.00994) | | |  |

ICC= Intra-Cluster Correlation coefficient

SE= Standard error

^¥^ICC in relationship to glycaemic control among participants with T2D in Uganda

^±^ICC in relationship to reduction in HbA1c > 3 mmol/mol among participants at high risk in Uganda

^§^ICC in relationship to glycaemic control among participants with T2D in South Africa

^€^ICC in relationship to reduction in HbA1c > 3 mmol/mol among participants at high risk in South Africa

**^a^** Adjusted for clustering within health facility of enrolment; and for age group, marital status, and baseline HbA1C

**^b^** Adjusted for clustering within health facility of enrolment; and for age-group, level of education, marital status, dietary score, and baseline HbA1c.

**^d^** Adjusted for clustering within health facility of enrolment; and for alcohol use and baseline HbA1C.

**^e^** Adjusted for clustering within health facility of enrolment; and for age group, marital status, alcohol use, and baseline HbA1c.
