# Supplementary material for: Prevention and management of type 2 diabetes mellitus in Uganda and South Africa: Findings from the SMART2D pragmatic implementation trial
Source: PLOS Glob Public Health. 2022 May 2;2(5):e0000425. doi: 10.1371/journal.pgph.0000425 (PMC10021626; doi:10.1371/journal.pgph.0000425)
Supplement: S1 Text — (DOCX) [file pgph.0000425.s002.docx]

**
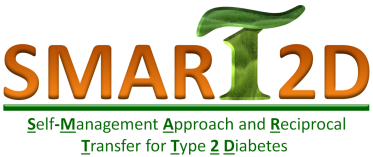
**

**THE EFFECTIVENESS OF FACILITY PLUS COMMUNITY INTERVENTIONS FOR THE PREVENTION AND MANAGEMENT OF TYPE 2 DIABETES: A MULTI-SITE CLUSTER RANDOMIZED TRIAL IN UGANDA, SOUTH AFRICA, AND SWEDEN**

**A proposal for Work Packages 4, 5 & 6 (WP4, WP5 & WP6) for the project titled: “A people centered approach to Self-Management and Reciprocal Learning in the prevention and Management of Type 2 Diabetes (SMART2D)**

#

Table of Contents

[TRIAL Protocol Team iii](#_Toc491420981)

[LIST OF ABBREVIATIONS, ACRONYMS, AND DEFINITIONS v](#_Toc491420982)

[1.0 INTRODUCTION/ BACKGROUND 6](#_Toc491420983)

[1.1 Background 6](#_Toc491420984)

[1.2 Lessons from the formative research studies (WP2 & WP3): 10](#_Toc491420985)

[1.3 Rationale for proposed trial 11](#_Toc491420986)

[2.0 Objectives 12](#_Toc491420987)

[2.1 General objective 12](#_Toc491420988)

[2.2 Specific objectives 12](#_Toc491420989)

[2.2.1 Primary objectives 12](#_Toc491420990)

[2.2.2 Secondary objectives 12](#_Toc491420991)

[3.0 DESIGN AND METHODS 13](#_Toc491420992)

[3.1 Study Design: 13](#_Toc491420993)

[3.2 Study Arms: 13](#_Toc491420994)

[3.3 The interventions 13](#_Toc491420995)

[3.3.1 Key Findings from Intervention Planning Workshops 13](#_Toc491420996)

[3.3.2. Facility Strategies 19](#_Toc491420997)

[3.3.2.1 A generic model for the SMART2D Facility Intervention 19](#_Toc491420998)

[3.3.2.2 Training for Health Facility Strategies 23](#_Toc491420999)

[3.3.2.3 Cultural Adaptation and Implementation of Health Facility Strategies in Each Site 24](#_Toc491421000)

[3.3.3. Community Strategies 31](#_Toc491421001)

[3.3.3.1 A generic model of the SMART2D Community Intervention 31](#_Toc491421002)

[3.3.3.2 Training for Community Strategies 33](#_Toc491421003)

[3.3.3.3 Cultural Adaptation and Implementation of Community Strategies in Each Site 35](#_Toc491421004)

[3.4 Study sites: 39](#_Toc491421005)

[3.4.1 Uganda: 39](#_Toc491421006)

[3.4.2 South Africa 42](#_Toc491421007)

[3.4.3 Sweden: 43](#_Toc491421008)

[3.5 Study Participants: 45](#_Toc491421009)

[3.6 Recruitment and enrollment of participants 46](#_Toc491421010)

[3.6.1 Uganda 46](#_Toc491421011)

[3.6.2 South Africa 48](#_Toc491421012)

[3.6.3 Sweden 49](#_Toc491421013)

[3.7 Baseline Evaluation: 51](#_Toc491421014)

[3.8 Follow-up evaluations: 51](#_Toc491421015)

[3.9 Outcomes of interest: 55](#_Toc491421016)

[3.10 Sample size Determination 55](#_Toc491421017)

[Sample size per arm, after further adjusting for estimated loss to follow-up of 10% 59](#_Toc491421018)

[3.11 Quality Control Measures 61](#_Toc491421019)

[3.12 Data Management Plan: 62](#_Toc491421020)

[3.12.1 Data collection tool development plans 62](#_Toc491421021)

[3.12.2 Database development plans 62](#_Toc491421022)

[3.12.3 Data management 62](#_Toc491421023)

[3.13 Statistical analysis Plan 62](#_Toc491421024)

[3.13.1 Primary intervention efficacy analysis: 62](#_Toc491421025)

[3.13.2 Analyses of secondary objectives 63](#_Toc491421026)

[3.14 Ethical Considerations 64](#_Toc491421027)

[4.0 References: 65](#_Toc491421028)

[5.0 APPENDICES 70](#_Toc491421029)

[5,1 Planned Timeline 70](#_Toc491421030)

[72](#_Toc491421031)

# TRIAL Protocol Team

| ***LEAD investigators*** |
| --- |
| David Guwatudde (BStat, MSc, PhD),  Dept. of Epidemiology and Biostatistics,  Makerere University School of Public Health,  Kampala Uganda. |
| Meena Daivadanam (MBBS, MPH, PhD),  Dept. of Food, Nutrition and Dietetics, Uppsala University &  Dept. of Public Health Sciences, Karolinska Institutet,  Sweden. |
| Pilvikki Absetz (PhD),  Collaborative Care Systems Finland (CCSF),  Finland. |
| Thandi Puoane (MD, PhD),  University of Western Cape, School of Public Health,  South Africa. |

| **Co-investigators** |
| --- |
| Peter Delobelle (MD, PhD),  University of Western Cape School of Public Health, South Africa. |
| Josefien van Olmen (MD, MPH),  Institute of Tropical Medicine, Antwerp (ITM), Belgium. |
| Roy William Mayega (MBChB, MPH, PhD),  Dept. of Epidemiology and Biostatistics,  Makerere University School of Public Health,  Kampala Uganda. |
| Helle M. Alvesson (PhD),  Department of Public Health Sciences, Karolinska Institutet, Sweden. |
| Elizabeth Ekirapa (MBChB, MPH, PhD)  Dept. of Health Policy, Planning and Management,  Makerere University School of Public Health,  Kampala Uganda |
| Juliet Kiguli (BA, MA, PhD)  Dept. of Community Health and Behavioral Sciences,  Makerere University School of Public Health,  Kampala Uganda |
| David Sanders (PhD),  University of Western Cape School of Public Health, South Africa. |
| Stefan Peterson (MD, MPH, PhD),,  Division of Global Health, Karolinska Institutet, Sweden. |

# LIST OF ABBREVIATIONS, ACRONYMS, AND DEFINITIONS

|  |  |
| --- | --- |
| AIDS | Acquired Immuno Deficiency Syndrome |
| HDREC | Higher Degrees, Research and Ethics Committee |
| HIC | High Income Countries |
| HIV | Human Immuno-deficiency Virus |
| IDF | International Diabetes Association |
| IMHDSS | Iganga-Mayuge Health and Demographic Surveillance Site |
| LIC | Low Income Country |
| LMIC | Low and Middle Income Countries |
| MakSPH | Makerere University School of Public Health |
| MIC | Middle income Country |
| SMART2D | A people centered approach to Self-Management and Reciprocal learning in the prevention and management of Type 2 Diabetes |
| T2DM | Type 2 Diabetes Mellitus |
| UNCST | Uganda National Council for Science and Technology |
| WHO | World Health Organization |
| USD | United States Dollars |
| CHW | Community Health Worker |
| FRA | Field Research Assistant |
|  |  |
|  |  |
|  |  |

#

# 1.0 INTRODUCTION/ BACKGROUND

## 1.1 Background

Type 2 diabetes mellitus (T2DM) and pre-diabetes (impaired glucose tolerance and/or elevated fasting glucose) contribute increasingly to the global burden of disease [WHO 2009]. Health systems in most parts of the world are struggling to diagnose and manage T2DM effectively. By 2035, the most dramatic change in the epidemiologic landscape is expected to come from the projected increase in T2DM cases in Sub-Saharan Africa (SSA) from 20 million in 2013 to 41 million, an increase of more than 100% [IDF 2015]. Worst, the proportion of undiagnosed diabetes, is relatively higher with a minimum of 27% in all regions [IDF 2015]. Even in Europe, of the overall prevalence of 7%, 37% of these cases are undiagnosed [IDF 2015]. Of all the T2D cases in the world, 80% live in low- and middle-income countries (LMICs), but low-income countries in Africa have the highest estimated proportion of undiagnosed diabetes estimated at 78% [IDF 2015].

The economic cost of diabetes is also increasing over time both for the individual, and the health system mainly due to complications such as cardiovascular disease, end-stage renal disease and foot ulcers [Caro et al. 2002; ADA 2013]. The mean health expenditure due to diabetes per person with diabetes was in 2011 approximately only 45 USD in low-income countries as compared to 5000 USD in high-income countries, with the additional spending probably contributing to the lower mortality rate, prevalence, and total deaths [IDF 2015]. The corresponding figures for middle-income countries ranged from 140-760 USD [IDF 2015]. One of the main risk factors for T2DM is pre-diabetes; where the annual risk for development of T2DM is about 10-15% [Fukui et al. 2011; Metzger et al. 2010]. Both T2DM and pre-diabetes have similar risk factors; including family history for diabetes, hypertension, hyperlipidaemia, physical inactivity, overweight or obesity, previous gestational diabetes and non-European ethnic background [Fukui 2011; Metzger 2010].

Preventing or delaying T2DM onset and its complications through appropriate strategies that include effective management and control of blood sugars is therefore, the primary focus of the SMART2D Project. Taking into account, the global nature of this problem, this the SMART2D Project focuses on three settings and three population groups namely, rural population in a low-income country, Uganda; urban population in a middle-income country, South Africa; and urban vulnerable immigrant populations in a high-income country, Sweden. In particular the focus is on implementation research to get existing interventions to reach those who would benefit from them.

*T2DM as a burden on the healthcare systems*

Healthcare systems in high-, and in particular, in low- and middle-income countries are poorly equipped to tackle the T2DM epidemic [Samb et al. 2010]. Moreover, health care professionals are more oriented towards medical care and often at the expense of preventive care [Woolf et al. 2007].

The Swedish health care system for example, has historically had a strong emphasis on equity [Jakubowski et al. 1998; Burstrom 2009]. However, the traditional focus on hospital-care has led to a weaker status and integration of primary care; and in spite of the above-average health indicators, Sweden faced a widening of the gap in health status by social class even in the 1990s [Jakubowski 1998]. With the rising prevalence of T2DM and the current immigration scenario, this is likely to worsen. An ageing population will further add to the burden. Currently, the prevalence of diabetes in Sweden is less than 5% [Hemminki et al. 2010], while that of pre-diabetes among adults aged 35-55 years is about 6% [Alvarsson et al. 2009]. In a high-income country like Sweden, the main problems are: 1) inadequate identification of those at risk due to the focus on hospital-based care; 2) poor integration and contextualization of evidence-based practices for lifestyle modification and disease management into routine care; and 3) inadequate focus on vulnerable population groups in prevention and management of T2DM.

The gaps in low- and middle-income countries (LMICs) predominantly relate to: 1) several health systems building blocks in terms of weakness in health policies and guidelines (governance), lack of finance, human resources and medicines, and inadequate coverage of service delivery [Tumusiime et al. 2012], which leads to poor provision of diagnostics and care; and 2) lack of focus on prevention efforts leading to an increase in prevalence of the high-risk pre-diabetes state. The national health systems in many LMICs, like in Uganda, may be weak, and service provisions may often be heavily skewed in favour of urban areas e.g. in South Africa and Uganda [Apoc 2007]. Health systems are primarily designed to tackle the high burden of communicable diseases and other related conditions, i.e., the care of acute infections, under-nutrition and maternal and child health conditions [Atun et al. 2013]. Into this mix we now add the challenge of rapidly rising chronic non-communicable diseases like T2DM, which is expected to hit Sub-Saharan Africa the hardest over the next two decades [Lozano et al. 2012; Dalal et al. 2011]. With the underfunded and understaffed health systems, this will stretch further an already overstretched system [Samb 2010].

In South Africa, a middle-income country in Sub-Saharan Africa, the national prevalence of T2DM is around 8%, ranging from 4% among the white population to about 13% among those of Indian origin[Distiller et al. 2010; IDF 2015]; and rising rapidly, especially among the disadvantaged population in the townships [Peer et al. 2012]. A study in 2012 revealed an age-standardised prevalence of 13.1% for T2DM among urban-dwelling black South Africans, with a large proportion of these being undiagnosed, and less than 40% being treated [Peer 2012; Bertram et al. 2013]. South Africa has guidelines for diabetes management focusing on patient education, lifestyle modification and pharmacotherapy with metformin as the initial drug of choice [Guideline Committee 2009]. However, compliance to these guidelines has been poor; and prevention of T2DM through risk reduction is not addressed.

In Uganda, a low-income country, the rural prevalence among adults aged 30 years or older, of T2DM is about 7% and of pre-diabetes is 20% [Mayega et al. 2013]. However, diagnostic facilities for T2D are available only at the higher health centres starting at health center level IV (Health Centre IV serves population of approximately 100,000 people), with almost no diagnostic and care capacity for T2D below this level [Mayega 2014]. The increase in number of diabetes cases, and the skewed distribution of care and diagnostic facilities, will over-burden the system; draining the few functioning centres, while leaving an even larger section of the population without adequate access [IDF 2015; Mbanya et al. 2010]. Moreover, those at high risk for T2DM are largely ignored because of low capacity [Mayega 2014].

*Need for community participation*

There is convincing evidence that lifestyle interventions can prevent or at least delay the onset of diabetes in individuals with pre-diabetes [Eriksson et al. 1991; Tuomilehto et al. 2001; Pan et al. 1997] and delay the onset of complications in those with T2DM. The value of delaying or preventing the development of T2DM can be seen from a societal as well as from the perspective of the individual's health and quality of life. However, this requires more than a bio-medical approach to the problem. A multidisciplinary approach focusing at an individual in terms of his or her family, community and environment and their inter-linkages and inter-dependencies is more relevant. This is in sharp contrast with the medical approach where people with an illness (like diabetes or those living with HIV or AIDS) were considered ‘patients’ dependent on medical providers [van Olmen et al. 2012]. Approaches that include community or outreach components to reach those who are not accessing the health system for one reason or other would therefore be very appropriate. However, this becomes a challenge with the current structure of the Swedish health care system, where patients are customers and the concept of catchment area for each primary care centre is no longer valid. It is therefore imperative that more innovative approaches are tried to expand the health system beyond its formal boundaries and link it to those in need.

Many of the SSA countries have successfully experimented with and developed community-directed or community empowerment approaches that establish ‘care structures’ at grassroots level where no routine health systems exist like, the African Programme for Onchocerciasis Control and Integrated Community Case Management (iCCM) [Apoc 2007; Nalwadda et al. 2013], which is more in line with the ideology of people-centred health systems [WHO 2013]. iCCM is now WHO/UNICEF policy and is being scaled up across the continent drawing on community level workers for prevention as well as curative care. Similar developments to draw on community resources to tackle preventive and curative newborn practices are following, and community agents play an important role in HIV prevention and care in may health systems. T2DM is a ‘chronic life long condition’ similar to that of HIV and AIDS and hence there are parallels that can be drawn and incorporated from the HIV-related experiences in SSA [van Olmen 2012][Rabkin et al. 2012]. Integrated HIV care and task shifting all the way to the primary health care level and use of expert patients and peer support groups were utilized to expand care networks and limit the heavy caseload at already understaffed health care facilities [Decroo et al. 2013; Decroo et al. 2012; Schull et al. 2011]. Using a well-coordinated scale up strategy, struggling health systems have been able to roll out basic and comprehensive HIV care to the lowest of primary care facilities, maintaining acceptable quality and high levels of adherence to treatment [Chalker et al. 2013].

Developing and expanding the reach of the health system right down to the community level through these proven strategies, can reduce the burden on the formal health system, while increasing its effectiveness [Samb 2010]. It would be highly relevant and likely cost-effective to borrow these ideas and utilize the facilities, communities or networks where applicable, to promote and enable self-management for diabetes and other non-communicable diseases. Expanding care networks through peer support and other community networks and empowering patients and their families to manage their own illness is as valid an approach for T2DM as it was for HIV and AIDS. Even drawing parallels to the HIV and AIDS experience, the key component that has been lacking in T2DM care and prevention models is the involvement of communities in prevention, care and lifestyle support. Provision of prevention efforts targeting lifestyle factors through the formal healthcare system becomes both a resource- and time-intensive exercise, as compared to peer-, family- or community-led approaches. With the increasing healthcare costs and burden on the qualified health personnel, even high-income countries like Sweden will find it difficult to maintain such healthcare practices over time. Lifestyle interventions can be delivered and maintained effectively through non-physician and even non-health care personnel, including family members, volunteers, peers or patients themselves [Diabetes UK 2009].

*Contextualization as a key factor*

Contextualization is relevant when adapting evidence-based interventions from high-income settings so that it suits the realities, including specific strengths and limitations of the LMIC setting; and also for the practical implementation of health guidelines or interventions with respect to the immigrant populations in high-income settings. This requires taking an inside, “emic” view, and not just a biomedical “etic” view. Studies in rural Uganda for example, have not only demonstrated low levels of awareness about lifestyle diseases [Mayega et al. 2012], but also misconceptions about obesity as a sign of ‘success’, ‘affluence’, or ‘having a good life’ [Mayega et al. 2014]. Moreover, the management of T2DM in Uganda still largely takes place in secondary care facilities. The health care packages of lower level health centres do not include provisions for prevention, risk assessment or chronic care [Mayega 2014]. As a result, patients have to travel long distances to seek care while preventive education for lifestyle diseases is not routinely provided in the integrated health education sessions. Nutrition education that is offered to care-takers of children often focuses on under-nutrition and less on over-nutrition. Patients treated with insulin face special challenges as there are no refrigerators in the rural homes. Anecdotal evidence also points to low levels of awareness about lifestyle diseases among healthcare workers [Mayega 2014].

Like most of Europe, Sweden’s population profile has become more multi-cultural over the past few decades, with the steady rise in immigration. People with a foreign background now comprise 21% of the population, including those born outside Sweden (16%) and those with both parents born outside Sweden (5%). The ethnic mix is quite eclectic with about 49% coming from Europe and North America (predominantly Finland, Poland and Turkey), and 51% from non-European countries. The non-European immigrant groups mainly come from

Asia (36%), particularly the Middle East – Iraq, Iran and Syria; Africa (10%), mainly

Somalia; and Latin America (5%). Several studies have demonstrated a higher prevalence and earlier onset of T2DM [31,32] and other non-communicable diseases (NCD); and a higher prevalence of behavioural risk factors like overweight, obesity, leisure-time physical inactivity and unhealthy dietary habits among immigrant populations of non- European origin as compared to native Swedes [Steiner 2013; Wändell et al. 2007; Jonsson et al. 2002; M. Lindström et al. 2001].

A study among 16 EU countries, including Sweden, identified best practices in healthcare for immigrants and recommended culturally sensitive health services; and enabling support networks as two of the eight essential elements [Priebe et al. 2008]. On the other hand, cultural differences relating to health communications, dietary requirements and gender roles were identified as one of several problems [Priebe 2008]. Most of the non-European immigrant populations in Sweden come from a more collectivist background as opposed to the individualistic nature of the modern society in Sweden [Inglehart et al. 2010]. Although several resources for lifestyle change are available within primary care services in Sweden, these programs are not culturally adapted [Saha et al. 2013]. Additionally, there are no guidelines or programs to assist health care providers in delivering culturally adapted lifestyle interventions [Saha 2013]. Sweden is one of the few countries in the world, which has National Guidelines for Prevention, with specific recommendations for tackling unhealthy lifestyle habits at the primary care level [Socialstyrelsen n.d.]. The government has also provided monetary support to professional associations (doctors, nurses, physiotherapists) for education and training on lifestyle modification methods. While the guidelines state that healthcare should reach out to socially disadvantaged groups in general, it does not really address the needs of individuals from different cultural backgrounds. The National Guidelines for Diabetes Care [Socialstyrelsen Sweden n.d.] also emphasises the need for culturally adapted training programmes, but without any specific direction as to how this should be achieved.

Research is also scarce on the effectiveness of lifestyle interventions in high-risk immigrant populations in Sweden with different cultural and socioeconomic backgrounds. Gåfvels and Wändell found that educating immigrant patients in diabetes self-care was more complex compared to Swedish-born patients, as cultural beliefs often appeared as attitude towards the illness, and assumptions about its causes, treatment and consequences [Gåfvels et al. 2007]. A culturally adapted lifestyle intervention is also currently running in the Malmo region, where immigrants from Iraq at high risk for developing type-2 diabetes are targeted using a community- and primary health care-oriented prevention model [Saha 2013].

*A strong case for reciprocal learning*

Between our three settings, we have identified three strengths: pioneering and innovative programmes for individual physical activity on prescription and policies in terms on National Guidelines for Prevention in Sweden; strong community component for T2DM care in Uganda; and evidence-based community-care models for chronic conditions like HIV and AIDS in South Africa. In terms of prevention and T2DM care, each setting also has its own major gaps: the strong facility-based care practice in Sweden, which limits identification of at-risk individuals and contextualization possibilities for vulnerable immigrant populations outside the healthcare setting; and the lack of prevention efforts and guidelines in Uganda and South Africa due to inadequacies in health system building blocks or challenges in adopting innovations to the contextual realities.

The traditional development paradigm has focused on the flow of knowledge, skills, and ideas (or innovations) from the ‘north’ or more developed or high-income countries to the ‘south’ or the so-called developing or low- and middle-income countries [Syed et al. 2013]. There has also been a move in the opposite direction through a growing trans-disciplinary movement that is realising the potential of ‘reverse innovation’, to tap the potential of low-cost health innovations being developed in many LMICs for implementing cost-effective solutions in HICs [Syed 2013]. However, Máire Geoghegan-Quinn, Commissioner for Research, Innovation and Science at the European Commission has aptly commented that, “More broadly, international cooperation in research works as a kind of ‘science diplomacy’ helping to bring nations together, whatever their differences in culture, politics and wealth”. We are now moving towards ‘reciprocal learning’. The eight-year collaboration between several partners: the Rwandan Ministry of Health; the non-governmental organization, Partners In Health; Harvard Medical School; and the Brigham and Women’s Hospital in Boston has highlighted the potential for shared and reciprocal learning through collaborative work [Binagwaho et al. 2013]. This move towards ‘a learning health system’ through mutual capacity building [Binagwaho 2013], with gaps in one setting being filled by the strengths of another, irrespective of north-south or income divisions is more appropriate for today’s globalized world; and particularly relevant for the subject of this proposal, where potentially effective interventions exist, but innovations in delivery systems are required to realize their potential.

## 1.2 Lessons from the formative research studies (WP2 & WP3):

*Methods used:* A conceptual framework was built to analyze the health system, the environment, the community, the population and relevant contextual elements per site with respect to self-management. Based on this framework, a topic guide was developed to structure data per site and to make data comparable among sites. Re-translating these data to the conceptual framework allowed identification of potential strategies and cross-site analysis. Data were collected from individuals with diabetes or pre-diabetes, community members, and health professionals from an urban township in Cape Town, a rural Iganga and Mayuge Districts in Uganda, and immigrant populations in Stockholm. Data were collected through focus-group discussions, in-depth interviews, and observations. Data were analyzed by thematic analysis.

*Summary of findings:* In each site, data revealed a strong capacity of the environment to influence people’s behavior through a promotive culture, access to food and external conditions facilitating physical activity. Data suggest that family plays a crucial role in social support and that health services are not sufficiently tailored and lack autonomy-supportiveness. Data from the three sites revealed a weak link among the community platform, the proximal environment and professional health providers. Data from the Ugandan and South-African site revealed a lack of adequate knowledge on self-management among persons with diabetes, but a strong potential for self-management education through the community. Interviewees acknowledged the ability to manage oneself, and expressed openness to learn more about how to do so.

*Conclusions:* Our study suggests an important role of the proximal environment, the community and the family in self-management support for T2DM. The weak link among these elements calls for better coordination and communication. The data further suggest that more attention should be given to making services more autonomy supportive and better tailored to the individual; and that community interventions may be an appropriate strategy to do this.

## 1.3 Rationale for proposed trial

Utilizing findings from the formative research conducted as Work Package 2 (realist synthesis of evidence), and Work Package 3 (cross-learning on implementation of care) of the SMART2D project, we have conceptualized an interaction between health system and communities where the approach is to empower an individual with T2DM or pre-diabetes and his or her family to live a quality life with their chronic condition through supported self-management. In addition, we aim to optimize diabetes care delivery to improve patient management. We want to do so through proven strategies, like task-shifting to non-physician health care providers and community health workers, and expanding care networks through community-based peer support groups. The study is designed to evaluate interventions for the prevention and management of T2D that are feasible for scale up should they prove effective.

# 2.0 Objectives

## 2.1 General objective

The overall objective of the trial is to determine the effectiveness of the facility + community strategies intended to improve prevention, management, access and adherence to type 2 diabetes care; in comparison with facility only strategies. Elements of the facility + community strategies, as well as elements of the facility only strategies are described later in section 3.7.

## 2.2 Specific objectives

### 2.2.1 Primary objectives

The primary objectives of the trial are as follows:

1. To determine the combined effect of the facility+ community strategies, in controlling blood sugar levels among patients with T2D
2. To determine the effect of the facility+ community strategies, in reducing blood sugar levels among individuals with pre-diabetes
3. To determine the effect of the facility+ community strategies, on retention into care among patients with T2D.
4. To estimate the incremental provider cost per patient of managing patients with T2D, as a result of implementing the facility+ community strategies.
5. To estimate the incremental client cost per patient of managing patients with T2D, as a result of implementing the facility+ community strategies.

### 2.2.2 Secondary objectives

1. To determine the effect of the facility+ community strategies, in reducing the incidence of conversion from pre-diabetes state to diabetes state
2. To evaluate differences between the country sites, in regard to baseline context infrastructure situation for prevention and management of diabetes
3. To analyze the degree of implementation of the intervention elements in each country, and differences between the country sites, through a process evaluation at each site
4. To determine the effect of the interventions on: perceptions towards behavioral risk factors for diabetes; including physical activity and dietary behavior.

# 3.0 DESIGN AND METHODS

## 3.1 Study Design:

A stratified cluster-randomized trial design will be used. At each of the study sites, the clusters will be the catchment areas of participating health facilities. The health facility clusters will be randomized to either the facility alone arm, or the facility + community arm. Thus each health facility to participate in the trial, together with the community in its catchment area will be the units of randomization. Although the clusters will be the units of randomization, the enrolled individual participants will be the primary study units of measurement.

## 3.2 Study Arms:

Sweden and South Africa will use two study arms, one with facility only interventions as the control condition, and a second arm with facility plus community interventions. The intervention elements in each of these arms are described later in section 3,3.

In Uganda, because the proposed facility only interventions are not yet standard of care in the country, a third arm that will comprise of “current practices” in the prevention and management of type 2 diabetes will be used against which the effectiveness of facility only, and the that facility+ community intervention arms will be compared.

## 3.3 The interventions

The SMART2D Intervention study is designed to evaluate the added value of a community component intervention to optimal facility care, compared to optimal facility care only. The trial includes two study arms, the ***Health Facility alone*** as a control condition and ***Health Facility plus Community*** as the intervention condition. This intervention protocol includes the following sections: 1) A summary of key findings from Intervention planning workshops conducted at the three research sites – South Africa, Sweden and Uganda; 2) Facility Strategies: 2.1) A generic model of the SMART2D facility intervention strategies and components; 2.2) Training Program for Facility Strategies; 2.3) Cultural adaptation of the facility strategies; 3) Community Strategies; 3.1) A generic model of the SMART2D community intervention strategies and components; 3.2) Training Program for Community Strategies; 3.3) Cultural adaptation of the community strategies. The generic strategies were agreed upon in the general meetings and workshops of the SMART2D research group, and the cultural adaptations in intervention planning teleconferences and local workshops with the SMART2D WP4.

### 3.3.1 Key Findings from Intervention Planning Workshops

Intervention strategies for SMART2D were identified through mapping the patient process from pre-diabetes to diabetes in the community and in the health facilities in each of the three SMART2D research sites. For the mapping, findings from situational analysis, and knowledge transfer from HIV/AIDS research literature, stakeholder interaction, and other SMART2D sites were utilized in participatory workshops.

See Figures 1-3 for identified community connections and pathways for people with prediabetes and T2DM in Sweden and Uganda, and Table 1 for some potential platforms in South Africa. More information on the intervention planning process and stakeholder interaction can be found in the appendix: Internal Reports on SMART2D Intervention Development Workshops.

Figure 1: Community connection points for people with pre-diabetes and T2DM in Sweden


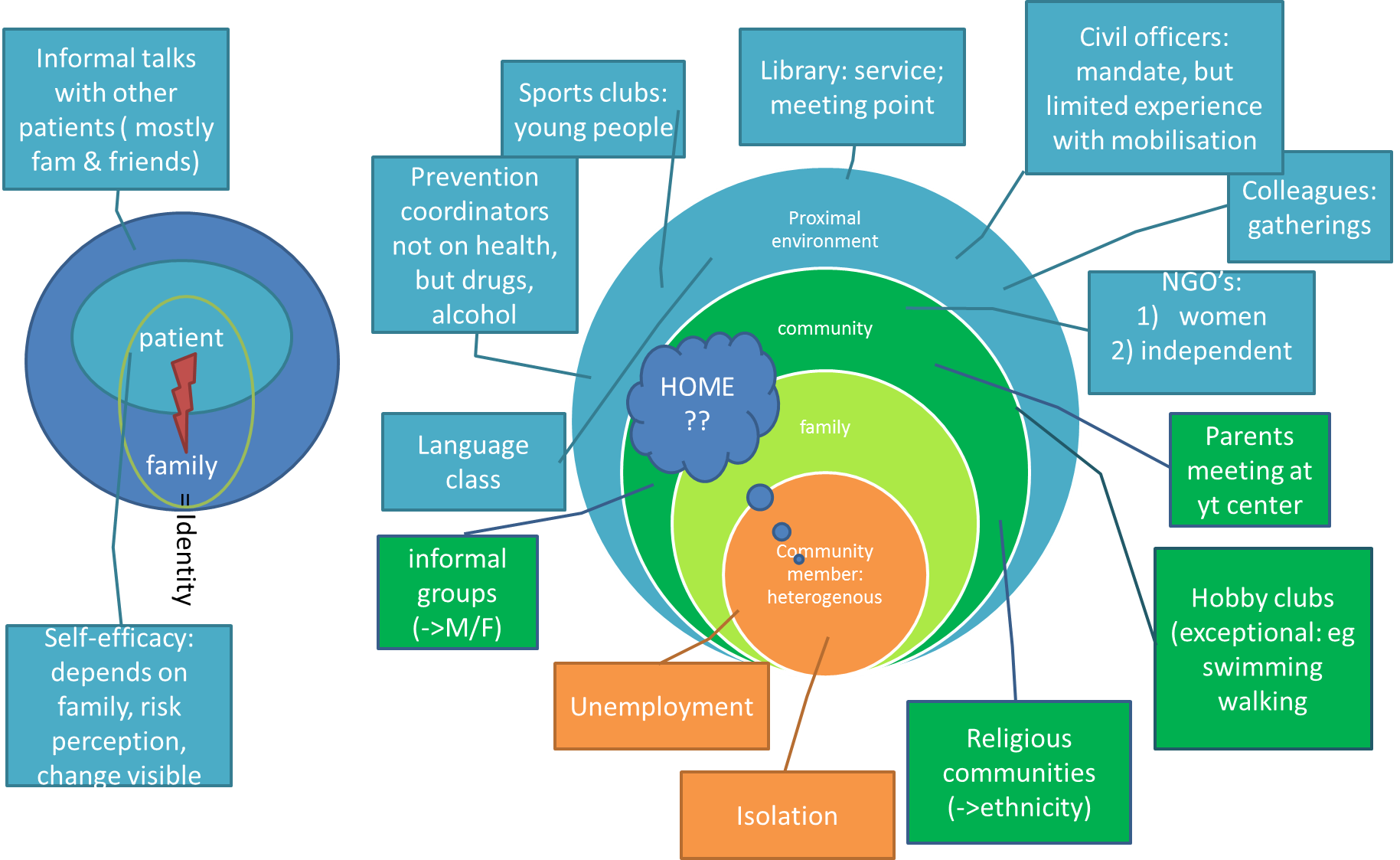


Figure 2: Facility care process of people with pre-diabetes and T2DM in Sweden

Figure 3: Facility care process of people with pre-diabetes and T2DM in Uganda

Table 1: Summary of potential platforms, strategies and partners identified in South Africa

| **Platform** | **Strategies** | **Partners** |
| --- | --- | --- |
| Support group (peer/comm’y): created subgroups with new patients (recruited for the study) | -Participatory development with intervention groups  -train facilitators (pt, nurse, CHW)  -Create a pool of interested patients (Expert patients) to become facilitator  -Tools & materials  -supportive supervision to facilitators | NGOs  Support group facilitators  Comm’y based services (DoH) |
| CHW (one to one) | -train in counseling skill  -basic information package | NGOs  Comm’y based services (DoH) |
| (linked with) facility | -discuss patient referral to groups (also new patients) |  |
| Care companion / family | -involve them  -tools & materials (also for the carer) | NGOs with experience (HIV/aids) |
| Community at large  (beware contamination) | -awareness raising events  -involvement of key people | KDF, several actors, local authorities |

Figure 4: A Generic Flowchart for the SMART2D Facility (blue) and Community (green) Intervention

Abbreviations: FPG=Fasting Plasma Glucose; CLT=Community Link Team; CC=Care Companion or Healthy Lifestyle Buddy; PGM=Peer Group Meeting

A generic intervention flowchart based on the workshop outcomes is shown in Figure 4. Main elements of the 12-month intervention are an organized care process that supports patient engagement (depicted in blue in the flowchart); social and physical environment supportive of healthy lifestyle and self-management of T2DM (depicted in green); and as part of the community intervention, a linkage between these two.

### 3.3.2. Facility Strategies

#### 3.3.2.1 A generic model for the SMART2D Facility Intervention

To optimize and standardize facility care across the two arms (as well as across sites, to the extent possible), key common elements related to the organization of care process need to be in place in all the health facilities. SMART2D will review the present care, checking the implementation of the minimum level of quality care elements as outlined below in table 2. Should key elements be missing, they will be implemented as part of the trial.

*Facility Strategy I: Organization of the care process*

The first part of the facility strategy entails the optimization of the organization of the care process, which is divided into three components.

Firstly, ***minimal infrastructure*** should be ***adequate***, i.e. being in place and functional. Minimal basic equipment present at all facilities delivering care is: a glucometer and a sufficient amount of test strips, weighing scales, measuring tapes (for waist circumference), blood pressure machines (preferably electronic) and a stadiometer. Newly bought machines should have a certified quality brand, and all machines need to be calibrated following the quality procedures for medical equipment. A ***Quality Control Checklist*** will help to assure that adequate infrastructure is in place across all facilities in the trial.

Secondly, ***guidelines*** should be implemented. In case they do not exist, they will be developed, based upon internationally available guidelines for diabetes care and management, but adapted to the local context. The follow-up appointments with full clinical evaluation are proposed at month 6 and 12 (and 18?), but the frequency of doctor / nurse appointments will be contextualized to each setting based on need and current practice – e.g., patients with unstable glucose levels will need more frequent appointments. Three main entities depicted in the guidelines are: 1) ***a clinical guideline*** for medical practitioners; 2) ***a task-shifting guideline*** to map the organization of care, identifying level of staff, tasks and responsibilities, and shifting these to a lower level where appropriate; and 3) ***a patient flow chart***, identifying the steps in the care process from the patient’s perspective.

Thirdly, a ***patient health information system*** should be in place to allow the longitudinal follow-up of patient, identification and tracing of those lost to follow-up.

*Facility Strategy II: Strengthening patient role in self-management*

The other main facility strategy, strengthening patient role in self-management, includes brief motivational coaching for behavior change (with focus on self-monitoring and goal setting), overview of care process and patient role in the management of DM2, and access to measuring devices.

Table 2: Elements of Minimum Level of Quality Care in the Health Facilities

| **FACILITY STRATEGIES** | **KEY ELEMENTS** | **SPECIFICATIONS** |
| --- | --- | --- |
| **I.** **Organization of care process** | a. Available and functioning minimal infrastructure adequate | i) Basic equipment for diagnostics and follow-up: glucometers, weighing scales, measure tapes, blood pressure machines, stadiometers/measuring boards |
|  |  |  |
|  |  |  |
|  | b. Guidelines and task identification | i) Clinical guideline |
|  |  |  |
|  |  | ii) Task shifting guideline |
|  |  |  |
|  |  | iii) Patient flow chart |
|  | d. Information system to follow up patients |  |
|  |  |  |
| **II. Strengthened patient role in self-management** | a. Brief motivational coaching for behavior change | i) Motivational coaching guideline / portfolio for groups and/or individuals |
|  | b. Overview of care process and patient role | i) Patient version of the patient flow chart |
|  |  | ii) Brief patient info / health education material |
|  | c. Access to measuring devices | i) Self-monitoring instructions  ii) Glucometers, weighing scales, measure tapes, blood pressure machines available at facility / from Expert patient^[[1]](#footnote-1)^ / in Patient peer group^1^ |

***Brief motivational coaching for behavior change*** is based on autonomy support integrated into a strength model and a participatory, appreciative approach, i.e., the aim is to identify existing healthy habits and self-management behaviors, and help the individual build further changes onto these strengths. It can be delivered either in individual or group sessions by nurses within the routine schedule defined in the care process at each site. The most simple, brief generic form of motivational coaching in individual sessions includes the following four steps (see Figure 5 for examples of specific questions for each step):

1. Identify what the patient already does and knows (=STRENGTHS).
2. Help to build from the strengths with one or two simple things the patient is willing to do (=GOALS).
3. Agree on and seek commitment for next steps (=ACTION).
4. Reinforce behavior and support learning at next encounter (=FOLLOW-UP).

When motivational coaching is delivered within group sessions, specific objectives of each session will determine the activities used. All activities will be embedded in a participatory framework applying the strength model. A generic structure for an introductory session delivered in a group (see table 3) includes activities that will provide an overview of the care process and the patient role, self-monitoring training, and distribution of health education material. Follow-up group sessions may focus on further adoption and maintenance of healthy dietary and physical activity habits (pre-diabetes) or address a broader range of self-management behaviors (diabetes). See Appendix for ***Motivational Coaching Guidelines***.

***Overview of care process and patient role*** will give the patient reassurance of the continuity of care, help him/her to understand the importance and role of self-management, and outline the different services available at the facility, and – in the Health Facility + Community arm of the study – the support systems available in the community.

***Access to measuring devices*** will be provided at health facilities and – in the Health Facility + Community arm of the study – in the communities. Patients will also be provided with written instructions and practical training in using the devices. The resource materials – ***A Brief patient information leaflet*** – will include an overview of the care process for patients, a brief patient information / health education package and instructions for using the measuring devices and other relevant self-monitoring, as well as a self-monitoring diary (see Appendix).

Figure 5: A Generic Model for Supporting Self-Management by Motivational Coaching in Individual Sessions

Table 3: A Generic Motivational Coaching Introductory Group Session for Patients with Type 2 Diabetes / Pre-Diabetes

_______________________________________________________________

#### 3.3.2.2 Training for Health Facility Strategies

SMART2D will organize training and mentoring / supervision for health workers to deliver the facility strategies (table 4). Our aim is not just to build the capacity for the SMART2D intervention delivery, but also to help build a trainer network that can eventually take over the training program to maintain activities also after the end of the SMART2D trial.

Training sessions will be organized for health facility staff on the organization of care and use of guidelines (where needed). SMART2D team will conduct the initial round of training workshops in all three sites and provide certification for the participating staff.

***Guideline training*** ***protocol*** will consist of the following elements:

- Refresh about diabetes
- Key aspects of clinical care with overview of the care process and patient flowchart
- Self-management needs
- Roles of health facility staff
- Specific context issues

Table 4: Training and Supervision/Mentoring for Implementation of Health Facility Strategies

| **FACILITY STRATEGIES** | **KEY ELEMENTS** | **TRAINING & SUPERVISION** |
| --- | --- | --- |
| **I.** **Organization of care process** | a. Available and functioning minimal infrastructure adequate | 1-day training for doctors and nurses including:   - Appropriate use of diagnostic devices - Use of information system - Clinical and task shifting guideline training   Quality assurance   - Guideline supervision |
|  |  |  |
|  | b. Guidelines and task identification |  |
|  |  |  |
|  |  |  |
|  |  |  |
|  |  |  |
|  | d. Information system to follow up patients |  |
| **II. Strengthened patient role in self-management** | a. Brief motivational coaching (MC) for behavior change | Half-a-day or 1-day training for nurses including:   - MC principles - Brief MC for individuals - Activities for group MC |
|  | b. Overview of care process and patient role | No separate training required   - Materials reviewed in guideline training - Implementation reviewed in MC training |
|  | c. Access to measuring devices | No separate training required   - Materials reviewed in guideline training - Implementation reviewed in MC training |

***Guideline supervision protocol*** will include brief repetition of guideline training and structure and tools for performing supportive supervision.

***Motivational coaching training*** ***protocol*** will include 1) a half-a-day / one-day training workshop structure and activities for nurses to acquire adequate skills to conduct the group and/or individual sessions. Resource materials supporting the training include ***Motivational Coaching – a Trainer’s Guide*** (see Appendix).

#### 3.3.2.3 Cultural Adaptation and Implementation of Health Facility Strategies in Each Site

Main adaptations and implementation of the Health Facility Strategies are listed in Table 5 and depicted in the site-specific flowcharts in Figures 6-8.

***In South Africa,*** a set of guidelines, the PC101 for symptom-based treatment of common conditions, has been developed for use in primary care (DOH. Primary Care 101: Symptom-based integrated approach to the adult in primary care. Pretoria: National Department of Health; 2013/14.). To strengthen patient role in self-management, individual motivational coaching focusing on self-monitoring and goal setting will be given during home visits by community care workers using parts of Diabetes South Africa health education material. Patient flowchart and measuring devices will be at the facilities, but all participants will receive a brief patient information material. The intervention process flowchart as adapted to South Africa is presented in Figure 6.

***In Sweden,*** organization of care process is already largely streamlined by the 4D-project (ref.). However, discussions with stakeholders have revealed a need to establish a routine to receive participants from screening at the health center for all procedures. Also, there is a need to establish a care pathway for pre-diabetes, which is currently non-existent. Nurses will be solely responsible for pre-diabetes.

To strengthen patient role in self-management, motivational coaching will be introduced with a structure and tools for visit 2, which is a key visit for both T2D and pre-diabetes. In the Community arm, a specific session will also be introduced into the facility for linkage with the community. A flowchart of the intervention process as adapted to Sweden is shown in Figure 7.

***In Uganda***, however, the minimal infrastructure is not yet adequate, guidelines have not been implemented, and information system is lacking, so all these aspects will be developed and implemented as part of the intervention (Figure 8). SMART2D will provide the devices necessary for diagnostics and follow-up as listed in Table 5. The appointment card, Patient register and Patient file will be developed and used to follow up patients. The patient register will include the participant’s telephone contacts and that of their care companion. Patient tracking will involve 2 steps:

1. Contacting the participant by telephone.
2. Contacting participants’ care companion by telephone if the patient was unreachable or failed to visit the clinic after being contacted on their mobile phone.

Patient flowchart from the clinical guidelines will be printed and displayed in the health facilities to guide both the participants and the health workers. A health worker will guide patients through the process. Separate flowcharts will not be provided for the patients, as most people in the study area are illiterate.

Motivational coaching will only be delivered in groups at months 3, 6, 12 & 18 at the study follow up visits. Motivational coaching guideline will give special tools for addressing those who are non-adherent to medication and those with poorly controlled blood glucose.

Self-monitoring will involve only monitoring of blood sugar using glucometer. Other measurements including weight, height and waist circumference will be done at the health facility during the follow up visits

Table 5: Elements of Minimum Level of Quality Care as Implemented in the Health Facilities Across the SMART2D Sites

| **FACILITY**  **STRATEGIES** | | **KEY ELEMENTS** | **South Africa** | **Sweden** | **Uganda** |
| --- | --- | --- | --- | --- | --- |
| **1. Organization of care process** | | a. Available and functioning minimal infrastructure adequate | Pre-existing but standardized for trial:   - Glucometers & strips - Automatic BP machines - Weighing scales - Stadiometers - Measuring tapes | Pre-existing but standardized for trial:   - Glucometers & strips - Automatic BP machines - Weighing scales - Stadiometers - Measuring tapes | By SMART2D:   - Glucometers & strips - HbA1C analyser & test kits - Automatic BP machines - Weighing scales - Stadiometers - Measuring tapes - Medicines to cover stock out |
|  |  | b. Guidelines and task identification | Pre-existing:   - PACK guidelines for health workers at primary care level   By SMART2D:   - Patient flow chart | Pre-existing:   - Clinical guidelines for T2DM, overweight and obesity, and healthy lifestyle   By SMART2D:   - Screening algorithm - Patient flow chart | By SMART2D:   - Clinical guidelines, including task shifting guidelines - Clinical treatment algorithm posters - Patient flow chart |
|  |  | d. Information system to follow up patients | Pre-existing:   - System for patient register with patient appointment cards | Pre-existing:   - Digital registration - System to trace/follow-up defaulters via mail and telephone (max 3 calls after 1 visit missed) | By SMART2D:   - Manual patient register filled in daily by HCW, with appointment diary, appointment cards and contact information forms - Patient tracking in two steps: 1) by phone to patient   2) via care companion (after each missed visit) |
| **2. Strengthen patient role in self-management** | a. Brief motivational, behavioural coaching for behaviour change | Pre-existing:   - Individual lifestyle counselling by HCW and health education delivered in groups by health promotors   By SMART2D:   - Skill-enhancement / standardization of delivery of motivational, behavioural coaching | Pre-existing:   - Individual lifestyle counselling by HCW   By SMART2D:   - Skill-enhancement / standardization of delivery of motivational, behavioural coaching | By SMART2D:   - Standardized health education and motivational, behavioural coaching in groups by HCW (documented guide provided) |  |
|  | b. Overview of care process and patient role | By SMART2D:   - Patient flowchart - Brief patient info / health education material | By SMART2D:   - Patient flow chart - Patient brochure and care companion role at visit 2 (or enrolment) by smart2d | By SMART2D:   - Brief patient info / health education material at enrolment |  |
|  | c. Access to measuring devices | No self-monitoring devices at facility  By SMART2D:   - Self-monitoring instructions in health education material | By SMART2D:   - Pedometers at visit 2 - Self-monitoring instructions in health education material | No self-monitoring devices at facility  By SMART2D:   - Self-monitoring instructions in health education material |  |

Figure 6: A Flowchart for the SMART2D Facility and Community Intervention as Adapted in South Africa

Figure 7: A Flowchart for the SMART2D Facility and Community Intervention as Adapted in Sweden

Figure 8: A Flowchart for the SMART2D Facility and Community Intervention as Adapted in Uganda

### 3.3.3. Community Strategies

#### 3.3.3.1 A generic model of the SMART2D Community Intervention

The SMART2D Community intervention arm comprises of the above outlined Health Facility strategies, as well as three main Community strategies: Community mobilization; Strengthening support from social and physical environment; and Community extension providing linkage between facility and community. Key elements for these strategies as well as specifications for generic tools are outlined in the table 6 below.

Table 6 SMART2D Community Strategies for the Health Facility + Community Arm

| **COMMUNITY STRATEGIES** | **KEY ELEMENTS** | **SPECIFICATIONS FOR TOOLS** |
| --- | --- | --- |
| **I. Community mobilization** | a. Messages on lifestyle and diabetes for community members / key stakeholders | i) One-pagers with key messages for: timely care seeking; risk monitoring & screening; healthy lifestyle |
|  |  |  |
|  |  |  |
| **II. Strengthen support from the environment** | a. Patient peer group program | i) Peer Group Guidelines with program topics and activities for monthly visits for one year |
|  |  | ii) Peer leader selection criteria |
|  |  | iii) Peer leader training protocol + Short guide to peer leader behaviors |
|  | b. Care Companion (CC) / Healthy Lifestyle Buddy (HLB) involvement | i) Booklet with CC / HLB role and task description |
|  |  |  |
|  | c. Promoting supportive physical environment | i) EPOCH manual and training slides  ii) PhotoVoice Training protocol and accompanying facilitator guidelines for PG’s. |
| **III. Community extension** | a. Community Link Team | i) Community Link Team guidelines  ii) Community Link Team training protocol |

*Community Strategy I: Community mobilization*

Community mobilization is designed to raise awareness of type 2 diabetes and it’s prevention and management through healthy lifestyle, timely seeking of care and self-management of the disease. It will be used to raise interest in participation in the trial, and it will also help to create and strengthen advocacy for healthy lifestyle in the communities, thereby supporting the goals and activities of the Peer group program, Care Companions / Healthy Lifestyle Buddies and Community Link Team. Key messages for community mobilization will address knowledge gaps identified in the formative research, and they will be compiled in collaboration with relevant stakeholder organizations. Materials for community mobilization will include one-pagers with the key messages and/or visuals. They will be distributed in the Health Facility + Community arm at community screening events, and through partner organizations, Community Link Team, and Peer groups.

*Community Strategy II: Strengthening support from the environment*

This strategy includes strategies for both social and physical environment. ***Peer group program*** will provide an arena for emotional support, practical support and information exchange for individuals living with diabetes and pre-diabetes. Participants in the Health Facility + Community arm are referred to a peer group in their first motivational coaching session. Peer groups meet monthly at a local community venue under the leadership of appointed ***Expert patients*** and co-leaders selected from within each group. A generic ***SMART2D Peer Group Program manual*** (see Appendix) includes topics and activities for 1) building rapport; 2) setting agenda for the group; 3) working with topics selected by the group; 4) receiving and giving ongoing support; and 5) evaluation and adjourning. In addition to the activities, each group meeting will include a brief physical activity break and opportunity for unstructured discussion and sharing. Peer groups are also open for family members and Care Companions / Healthy Lifestyle Buddies. ***Selection criteria*** and recruitment of Expert patients as Peer group program facilitators are outlined in the ***Peer group program manual*** (refer to Appendix). Peer leaders will be asked to give a written commitment for taking the role; in return they will get training, a SMART2D Peer leader certificate, a fixed budget for meeting-related expenses, and a small compensation for each group meeting.

In all the SMART2D sites, individuals with diabetes are already often accompanied by their significant others at the health facility. In the SMART2D intervention, we will enhance the ***involvement of*** ***Care Companions*** / Healthy Lifestyle Buddies (for individuals with pre-diabetes) who will be appointed by the participants to act as their confidants, supporters, and companions in managing their health and in leading a healthy lifestyle.

In both arms, participants will be asked to nominate a Care Companion / Healthy Lifestyle Buddy at the study enrollment visit at the facility.

In the Health Facility + Community intervention arm, the role of Care Companions / Healthy Lifestyle Buddies as supporters of the participants healthy lifestyle is further systematized and enhanced. A ***screening tool*** for appropriate candidates has been modified for pre-diabetes/diabetes by the SMART2D team based on the Personal network inventory instrument [Knowlton 2003]. ***Selection criteria*** of a Care Companion / Healthy Lifestyle Buddy have been modified based on work by Nachega et al (2010), Kunutsor et al (2011) and Duwell et al (2013) [Nachega et al. 2010][Kunutsor et al. 2011][Duwell et al. 2013] (for both, refer to Appendix: ***Care Companion guidelines***). The Care Companions / Healthy Lifestyle Buddies are invited to accompany the participants at health facility visits, and they will receive a ***Care Companion Information booklet*** at the first visit. They will also be encouraged to ***participate in the Peer group program meetings***, which include special activities and discussion points designed to help the Care Companions become effective supporters and to enable them to learn from each other.

The ***six*** ***key tasks for Care Companions*** / Healthy Lifestyle Buddies in supporting individuals with diabetes / pre-diabetes include three tasks for both arms and three additional tasks only for the Health Facility + Community study arm.

Tasks in both arms:

1. Remind the individuals or help them to set up a reminder system of clinic appointments, help them overcome any obstacles for attending, and accompany them when feasible.
2. Remind the individuals of or help them to set up a reminder system for taking diabetes medication as prescribed by the doctor and help them overcome any obstacles for it.
3. Remind the individuals of or help them to set up a reminder system for self-monitoring and help them overcome any obstacles for it.

Additional tasks in Health Facility + Community study arm:

1. Remind the individuals or help them to set up a reminder system to go for peer group sessions, help them overcome any obstacles, and accompany them when feasible.
2. Support the individuals in meeting daily/weekly physical activity goals by helping to create a family environment and processes enabling physical activity, and by inspiring, encouraging, joining in, and giving positive feedback.
3. Support healthy diet for the individuals by helping to create a family environment and processes enabling healthy eating, and by inspiring, encouraging, joining in, and giving positive feedback.

***Promoting Healthy Physical Environment.*** This strategy will entail actions embedded into the other strategies. For example, key messages of the community mobilization may involve calls for action to restructure the environment; peer group program activities may include e.g., learning of kitchen gardening, or clearing a safe walking path into the neighborhood; Care Companion tasks may be translated into redesigning the home environment into a healthier one; and the Community Link Team advocacy role may extend e.g., to promoting healthier food environment or safer environment for physical activity in the local community.

***Community walks with or without PhotoVoice*** will be implemented within the Peer group program. Originally, PhotoVoice is a research methodology in which research ‘subjects’ become co-researchers in investigating a given research question. In this instance, peer group participants, guided by their peer leaders will conduct walks in their community to investigate their own food and physical activity environments, and share their perspectives on their role in their management of diabetes. They can do this either through photography or journaling. Resulting observations can also be used for promotion/advocacy purposes.

*Community Strategy III: The Community Extension*

A linkage will be formed between facility and community to enhance the flow of information, feedback, and support vital for proper management of diabetes. SMART2D will help to set up and support the functioning of ***Community Link Teams*** (CLT) that will consist, ideally, of one-to-two Expert Patients; a representative of a local/regional NGO with relevant objectives and/or activities; a relevant community worker, e.g., a community health worker or a prevention coordinator; and a health facility worker (e.g., a nurse). The CLT’s tasks are to 1) advocate for healthy lifestyle in the community; 2) support implementation of all other community strategies; and 3) form a linkage between the facilities and the community.

#### 3.3.3.2 Training for Community Strategies

Table 7 outlines all the training activities required to deliver the generic community strategies. Most strategies are linked with peer group activities or facility care, hence training of peer leaders and health care workers (MC) includes training for these strategies as well.

***Peer group training*** protocol includes a training workshop for peer leaders for getting the peer group started, and further training and mentoring workshops or short sessions according to need to support the conduct of further meetings, guiding of the community walks, utilization of the CC and maintaining linkages with the facility. Resource materials include a ***Peer Group Guidelines*** document, and a ***Short*** ***Guide to Peer Leader Behaviors***, consisting of rapport building and autonomy supportive behaviors.

***Care Companion training.*** Instead of separate training program for Care Companions / Healthy Lifestyle Buddies, introduction and/or support for their role in the Health Facility + Community study arm will be integrated into other activities in the intervention:

1. Motivational coaching group visits at the health facility, to which they will be asked to accompany the individuals with pre-diabetes/diabetes.
2. Peer group meetings to which they will have open invitations.

***Training for Community Extension*** is integrated with the peer leader training, and with the HCW training program.

Table 7: Training and Supervision/Mentoring for Implementation of Community Strategies

| **COMMUNITY STRATEGIES** | **KEY ELEMENTS** | **TRAINING & SUPERVISION / MENTORING** |
| --- | --- | --- |
| **I. Community mobilization** | a. Messages on lifestyle and diabetes for community members / key stakeholders | No separate training required |
|  |  |  |
|  |  |  |
| **II. Strengthen support from the environment** | a. Patient peer group program | Training:   - One day skill-building workshop for peer leaders to get groups started: |
|  |  | - - Peer group principles and facilitation   - Peer group portfolio for themes and activities   - Practical organization of peer group |
|  |  | Mentoring:   - Half-a-day maintenance workshop/s for peer leaders to share and learn - Short mentoring sessions (f2f or group TC) |
|  | b. Care Companion (CC) / Healthy Lifestyle Buddy (HLB) involvement | No separate training for CC   - Training for conducting CC introduction as part of the MC session at facility integrated to HCW training - CC support integrated to PL training |
|  | c. Promoting supportive physical environment | Community walk overview as part of peer leader training |
| **III. Community extension** | a. Community Link Team | Training:   - Half-a-day introductory and skill building workshop   Mentoring:   - Short mentoring sessions (f2f or group TC) - Half-a-day closing workshop for evaluation and maintenance |

#### 3.3.3.3 Cultural Adaptation and Implementation of Community Strategies in Each Site

Adaptation and implementation of the community strategies across the three countries is described in Table 8. In all the three sites, the community mobilization, and the social support components of peer group program and care companions will be implemented in relatively similar ways and as depicted in the generic model. E.g., it is a relatively common practice for people across the three countries to have significant others as care companions when they visit health facilities. However, there are no systematic procedures or formal guidance for care companion selection or supporting their role in any of the countries. Hence, while care companions will function in both arms, SMART2D will provide guidance and support for the role in the community arm in each country. Furthermore, the care companions will be linked with other community activities such as the peer group meetings. The delivery of the physical environmental support component differs between the countries, with South Africa focusing most on this component and the other two countries implementing it as part of the peer group program. Also, much of the community intervention in South Africa will be delivered by the community care workers, who have a strong role in that setting. Finally, the community linkage is emphasized in Sweden with a multisectorial community link team.

Table 8: Community Strategies as Implemented in the Health Facility + Community Study Arm in the SMART2D study sites

| **COMMUNITY STRATEGIES** | **KEY ELEMENTS** | **South Africa** | **Sweden** | **Uganda** |
| --- | --- | --- | --- | --- |
| **1. Community mobilisation** | a. Messages on lifestyle and diabetes for community members / key stakeholders | Pre-existing:   - Diabetes SA leaflets distributed by SMART2D at community awareness raising event(s) | By SMART2D:   - Brochures on timely care seeking; risk monitoring & screening; healthy lifestyle distributed at different places & events in community | By SMART2D:   - Oral information at household visits for screening purpose |
| **2. Strengthen support from the environment** | a. Peer group program | By SMART2D:   - Peer group facilitator topic guide & manual for 9 sessions of separate groups for people with pre-diabetes and diabetes | By SMART2D:   - Peer group facilitator topic guide & manual for 9 sessions of separate groups for people with pre-diabetes and diabetes | By SMART2D:   - Peer group facilitator topic guide & manual for 9 sessions of mixed groups with people with pre-diabetes and diabetes |
|  | b. Care Companion (CC) / Healthy Lifestyle Buddy (HLB) involvement | CHWs will take on the task of CC / HLB  Pre-existing:   - PACK CHW guidelines   By SMART2D:   - Skill-enhancement of motivational, behavioural coaching to | CC is a family member, relative, friend, neighbour  By SMART2D:   - Brochure describing CC role and tasks - Identification of CC at facility visit 2 - Invitation to peer groups & activities | CC is a family member, relative, friend, neighbour  By SMART2D:   - CC Guideline for HCW to instruct patient and CC on CC selection and tasks - Identification at month 1(CC) /3 (HLB) - Invitation to HC visits and peer group meetings |
|  | c. Promoting supportive physical environment | By SMART2D:   - Community walks +/- PhotoVoice as part of peer group program | By SMART2D:   - Physical activity and healthy food related activities as part of peer group activities linking with community resources through municipalities / NGOs - Community walks as part of peer group activities | By SMART2D:   - Community walks as an optional activity for peer group |
| **3. Community extension** | a. Community Link Team | By SMART2D:   - Support to CHWs to act as liaison between facility and community. | Pre-existing:   - Citizen Offices with Prevention Coordinators and Citizen Hosts helping citizens and linking them with different public, private and non-governmental organizations and services.   By SMART2D:   - Utilization of the existing networks of Citizens Offices and bringing T2DM on their agenda - Mobilization for recruitment through the network - Linkage of the network with peer groups | By SMART2D:   - Formal introduction meeting between peer leader and facility nurses - Peer leaders to remind peer group participants to go to visits, contact with health facility if loss to follow-up/problems - HCW delivering health education remind participants to attend peer group meetings |

## 3.4 Study sites:

The trial will be conducted at the three SMART2D participating sites in Uganda, South Africa, and Sweden.

### 3.4.1 Uganda:

In Uganda, the trial will be conducted within the two districts of Iganga and Mayuge that host the Iganga-Mayuge Health & Demographic Surveillance Site (IMHDSS). The two districts are adjacent to each other and are located in eastern Uganda approximately 120 kilometers east of Kampala along the Kampala-Kenya Highway.

Briefly, Uganda implements a level based health care system. Health Centre level I (HC I) is the lowest level of health care serving at village level to approximately 1,000 people, and has no physical structure but is a team of community health workers (CHW). Health Centre level II (HC II) is at parish level serving approximately 5,000 people. It is headed by an enrolled nurse and has approximately 3 established staff. HC II implements the “minimum activity package” including treatment of common diseases (malaria, diarrhea, skin infections, etc), integrated preventive outreaches, immunization, antenatal care and referral. It is therefore possible to provide general health education on diabetes and non-communicable diseases at this level as well as basic messages on diet, physical activity and risky habits like smoking and harmful use of alcohol. Health Centre level II (HC III) is an intermediate referral level serving about 20,000 people at sub-county level, with additional services including normal deliveries, limited in-patient care and TB treatment. It is headed by a Clinical Officer and has a laboratory that conducts basic tests (malaria, urine, blood sugar and TB). At this level, it is possible in theory but not in practice to provide blood sugar tests for individuals with proximal risk factors and refer them for follow-up care. Health Centre level IV (HC IV) is mini-hospital referral unit serving about 100,000 people in a zone called a Health Sub-district, usually covering a county. They are headed by a medical officer and have nursing officers with diverse skills. HC IVs are supposed to provide comprehensive referral services as well as support to the lower health units in their catchment zone (county). At this level, it is possible to provide pragmatic follow-up programs for high risk groups identified through targeted screening and to coordinate and NCD prevention program for this health service zone. HC V is a district hospital, HC VI regional hospital, whereas HC VII the National Referral Hospital.

***Uganda health facilities to participate in the trial***

Only government owned public health facilities at level III to level V in Iganga and Mayuge Districts will participate in the trial. There are 4 HC IV and 19 HC III. The four HC IVs will all participate in the study, whereas 5 of the 19 HC III’s were selected to participate in the study, two from Mayuge District and 3 from Iganga District. The HC III facilities were selected such that they are reasonably far apart from each other, allowing for sufficient buffer zones between all participating health facilities to minimize chances of contamination between the trial arms. Table 9 gives the list of the selected health facilities, whereas Figure 9 shows the geographical location of the selected health facilities within the study area.

Table 9: Health facilities selected to participate in the trial

| **#** | **Name** | **Level** | **Sub-county** | **County** | **District** |
| --- | --- | --- | --- | --- | --- |
| **Iganga District** | | | | | |
| 1 | Lubira HC | III | Buyanga | Bugweri | Iganga |
| 2 | Busesa HC | IV | Ibalanku | Bugweri | Iganga |
| 3 | Bugono HC | IV | Nabitende | Kigulu North | Iganga |
| 4 | Namungalwe HC | III | Namungalwe | Kigulu North | Iganga |
| 5 | Busowobi HC | III | Nakigo | Kigulu South | Iganga |
| **Mayuge District** | | | | | |
| 6 | Kigandalo HC | IV | Kigandalo | Bunya East | Mayuge |
| 7 | Kityerera HC | IV | Kityerera | Bunya South | Mayuge |
| 8 | Baitambogwe HC | III | Baitambogwe | Bunya West | Mayuge |
| 9 | Mayuge HC | III | Mayuge Town Council | Bunya West | Mayuge |

Figure 9: Location of health facilities in Uganda selected to participate in the trial


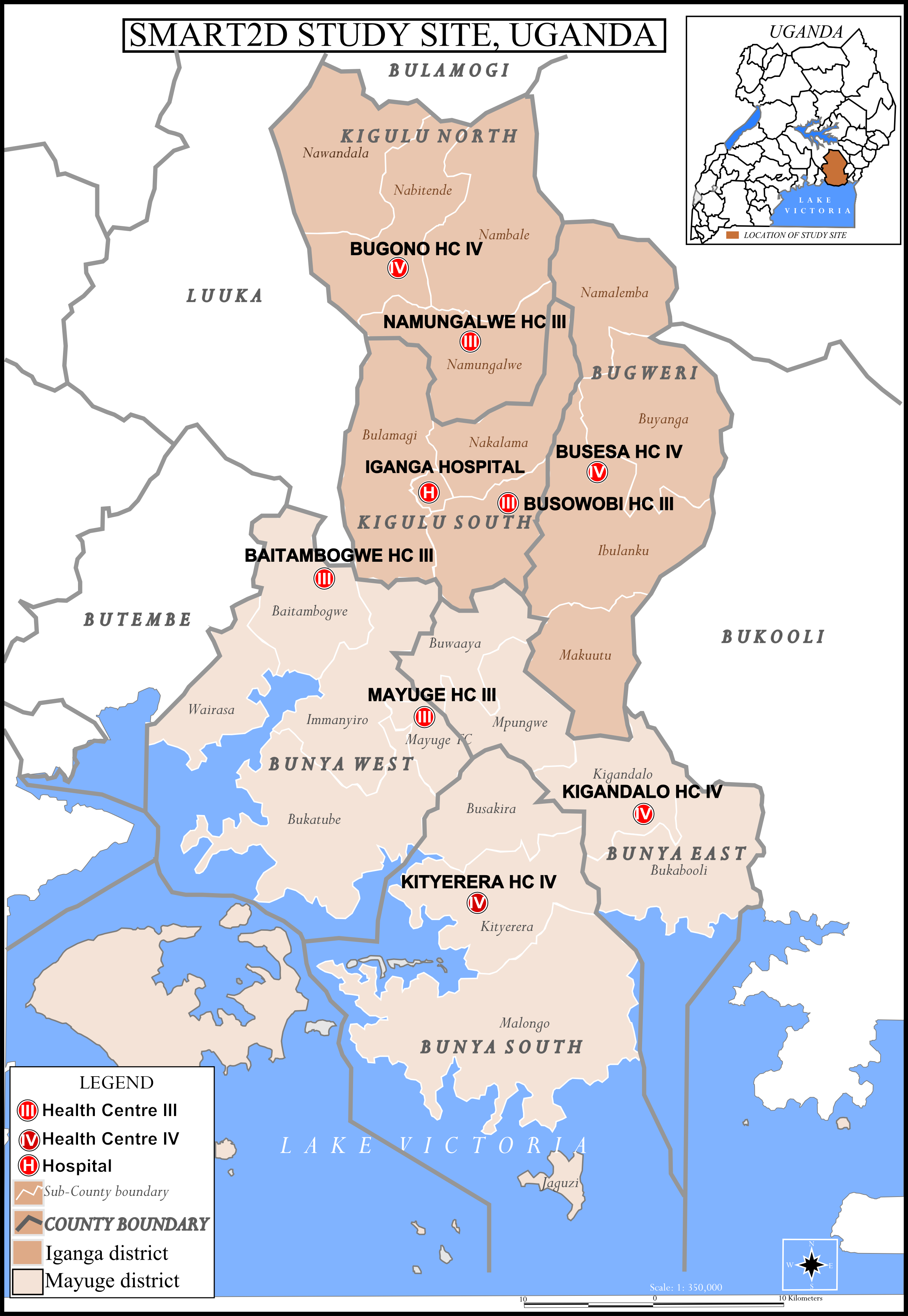


### 3.4.2 South Africa

In South Africa, the trial site will be conducted at Khayelitsha. According to the census data (2011), there are about 391 749 people living in in this urban township. About 36% of those aged 20 years and older have completed Grade 12 or higher and 38% of the labor force (aged 15 to 64) is unemployed. In addition only 45% of the population live in formal dwelling (Census, 2011).

The health care system is organized in three levels namely primary health care (clinics and community care centers), secondary (regional hospitals) and tertiary (tertiary hospitals) level care. Chronic care services are offered at the primary level care and there is a hierarchical referral systems where problem cases are referred to secondary level care. At primary level care there are also Community Based Services (CBS) are provided by Non-profit Organizations (NPOs)/ non-government organizations (NGOs), subsidized by the Provincial Government. The Western Cape Chronic Disease Policy has set up community-based support groups via local NPOs/NGOs - with input from the Community Health Centers. The implementation of support groups is seen as a way to improve adherence and increase self-management amongst patients. These community support groups are run by community health workers.

***South Africal health facilities to participate in the trial***

In South Africa, chronic care is offered at primary health care (PHC) facilities. In Khayelitsha PHC services are provided at 14 facilities but only 7 offer chronic care services (Table 10). These facilities are governed by 2 administrative authorities. Governed by the Provincial Government and Local Municipality (City of Cape Town)

Table 10: Facilities offering services to patients with chronic conditions

| **Name of Facility** | **Area** | **Administrative Authority** |
| --- | --- | --- |
| Khayelitsha Day Hospital/CHC | Site B | Provincial Department |
| Nolungile Day Hospital/CHC | Site C | Provincial Department |
| Michael Mapongwana Day Hospital/CHC | Harare | Provincial Department |
| Luvuyo Clinic | Makhaza | Local Authority |
| Kuyasa Clinic | Kuyasa | Local Authority |
| Matthew Goniwe Clinic | Makhaza | Local Authority |
| Town 2 Clinic | Town 2 | Local Authority |

Figure 10 shows the distribution of facilities offering services for chronic conditions that will participate in the study.

Figure 10: Location of health facilities in Khayelitsha, Cape Town, selected to participate in the trial

For the SMART2D intervention, only health facilities attached to a non-governmental organization utilizing CHWs will participate in the trial.

The SMART2D study sites will be 2 or four neighborhoods based on the facility that will be selected.

### 3.4.3 Sweden:

Migration has shaped Sweden over a long time. In the late nineteenth century 1.5 million Swedes migrated to America mainly due to poverty and to religious and political repression. During 1887 as many as 50,000 Swedes emigrated to the Americas. This record was only broken in 2011 when 51,000 Swedes emigrated to other countries in Europe, America and China; however the reasons were different (and the total population of Sweden about twice as large).

Currently immigration to Sweden involves two parallel processes: labor immigration and refugee immigration. An increase in political instability around the world has led to large groups of people fleeing their home countries, and immigration to Sweden has grown significantly. During 2014, the total population of Sweden increased by around 100,000 mainly due to refugees from Syria, Iraq, Afghanistan, Eritrea and Somalia seeking asylum in Sweden. Political unrest in Latin America and the Balkans in the 1970’s and 1980’s led to immigration waves from these areas. About 21 percent of the population in Stockholm is born outside Sweden or have parents not born in Sweden (16 percent in the nation).

Stockholm is a capital city characterized by a strong urbanization and with more than two million inhabitants– out of almost ten living in Sweden. The popularity of Stockholm is positive even though it brings challenges such as a competitive housing & job markets, pressure on schools and healthcare, and on traffic. Segregation and the development of an increasing number of neighborhoods with high immigrant density have emerged as a political issue during the last 20 years. These neighborhoods are composed of residents of many nationalities, in some cases from more than 100 countries. There are no neighborhoods where one single ethnic group comprises more than 10 percent of the population.

The SMART2D study sites will be in two or four such neighborhoods yet to be selected. The neighborhoods are geographically located in the suburbs of Stockholm in areas built during the 1960s as part of a “Million Homes Programme” that provided a million apartments over a short period of time. Rental housing dominates in these areas in addition to condominiums (*bostadsrätt*). The neighborhoods are considered to be socially vulnerable with low income levels and high unemployment rates. The mobility in these neighborhoods is higher than in areas where residents are mainly born in Sweden. The dynamics of mobility within segregated areas is a relatively new field of research in Sweden and findings from one recent study suggests that residents born in Sweden are less inclined than most migrant groups to move into migrant dense areas.

Healthcare in Sweden is publicly financed and the primary health care is the point of entry for persons seeking preventive or curative services. The primary healthcare centers provide the main part of the services for persons with diabetes and other chronic conditions. Diabetes rates are increasing in Stockholm. The share of persons diagnosed with diabetes was 4.6% in 2010 compared to 2.8% in 1990. Increasing overweight and obesity problems in the population are likely the main reasons for this trend. Socially disadvantaged neighborhoods have higher rates of disease, in particular diabetes. Women in areas with poor socioeconomic conditions have two to three times more often diabetes than women in the rest of Stockholm County.

***Sweden health facilities to participate in the trial:***

In Sweden, a health-centre and its catchment area (defined as the areas represented by the zip-code numbers of the patients currently registered in the health centre) will be designated as a cluster. The 4D project, jointly managed by Karolinska Institutet and the Stockholm County Council looks at four disease conditions within Stockholm County, of which diabetes is one. The Swedish arm of SMART2D is collaborating with the ‘diabetes’ part of the 4D project in Stockholm. As the 4D project has already developed a working collaboration with four primary care centres in Stockholm area to develop and implement quality control measures and to understand the healthcare-patient dynamics for diabetes, SMART2D will also be working in these centres. The municipalities being served by these four primary healthcare centres and their proportion of immigrant populations and the CNI index is given in Table 11 below. Figure 11 shows the geographical locations of the health centers selected to participate in the study.

Table 11: Municipalities considered for participation in the trial

| **#** | **Municipalities** | **% of migrants** | **CNI *** |
| --- | --- | --- | --- |
| 1 | Järfälla (Jakobsberg) | 36% | 1.42 |
| 2 | Hässelby-Vällinby (Hässelby) | 36% | 1.70 |
| 3 | Spånga-Tensta (Tensta) | >75% | 2.54 |
| 4 | Spånga-Tensta (Rinkeby) | >75% | 2.44 |

*Care Need Index - a social deprivation index

There are currently diabetes-focused interventions underway in Flemingsberg and therefore will have to be excluded. Of the remaining four, Tensta and Rinkeby are migrant-dense areas (>75%) with more mobile and socio-economically disadvantaged populations. Jakobsberg and Hässelby on the other hand have comparable proportion of immigrants (36%), who are more stable and socio-economically more advanced.

Figure 11: Location of health facilities in Stockholm Sweden, selected to participate in the trial


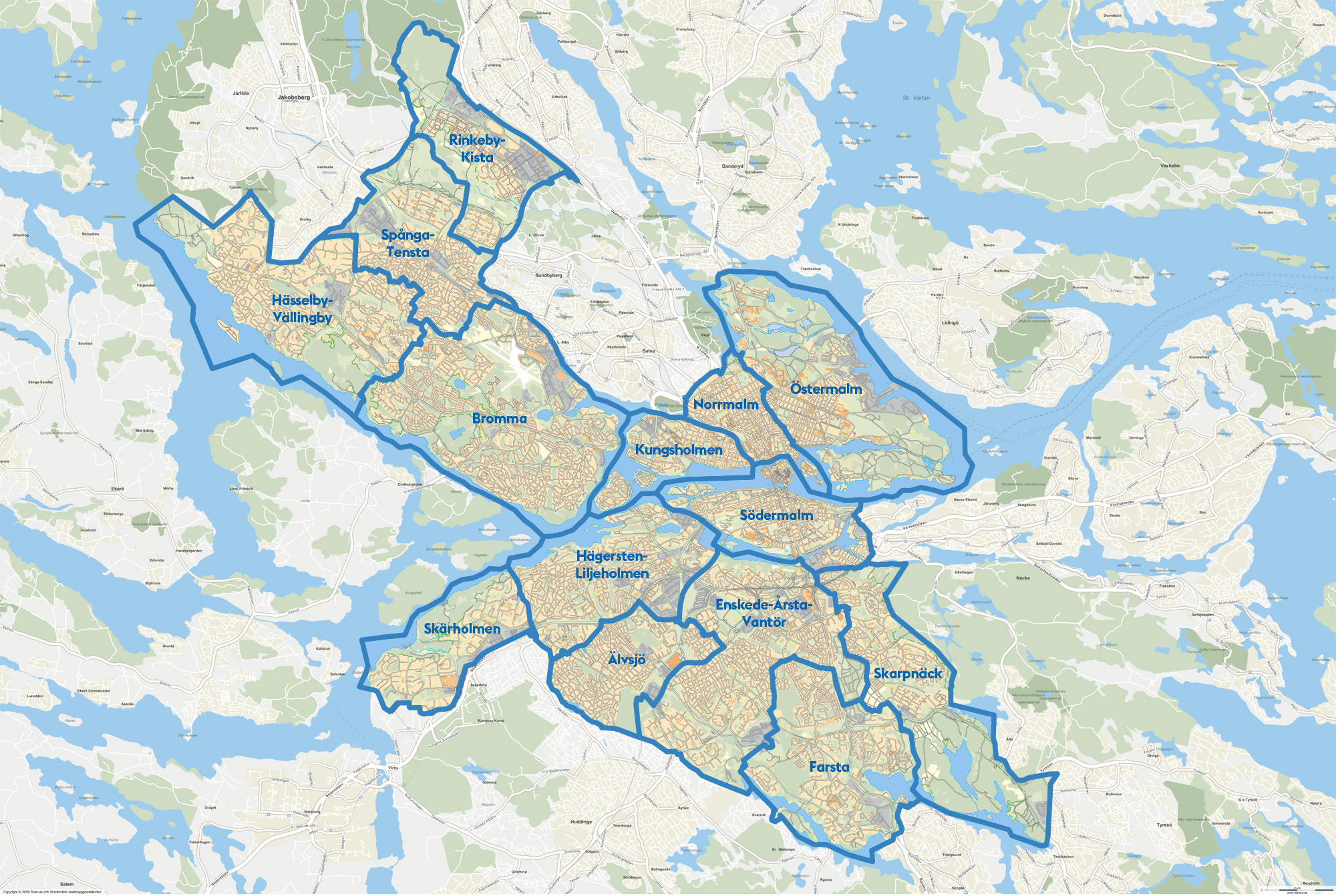


## 3.5 Study Participants:

Participants will be residents within the identified catchments areas at each of the study sites as described above. Eligible subjects will be:

1. current residents who have resided in their respective communities for at least 6 months prior to enrollment into the study,
2. aged 30 – 75 years,
3. currently not known to be pregnant
4. have no plans of migrating out of the study area over the next 12 months from the date of enrollment into the study,
5. able to provide written informed consent,
6. agree to allow home visits and follow-up contacts as part of participating in the trial,
7. no previous diagnosis with diabetes, or diagnosis of diabetes of no longer than 12 months prior to being screened for enrollment into the study
8. have a positive confirmatory test of pre-diabetes or diabetes

## 3.6 Recruitment and enrollment of participants

Prior to initiation of the trial, the pre-identified health facility clusters will be randomized either to the facility only, or to the facility plus the community arm al the three country sites; and additional to the current practice arm in Uganda. Each site will screen subjects in their study population to identify potential trial participants, using different algorithms as described below.

### 3.6.1 Uganda

***Community sensitization:***

In Uganda, engagement of the community will begin with sensitization of the political and health service delivery leadership in the two districts of Iganga and Mayuge. A number of meetings will be conducted in which the study team will provide the necessary information about the planned study to the district health and political leaders in the two districts. The leaders to be targeted will include the District Health Officers of the two districts, local council chairpersons, and the in-charges of the health facilities to participate in the study. Study investigators will make presentations to the community leaders to explain the objectives of the study, the participant recruitment process, procedures to be conducted on enrolled participants, the benefits, and the risks of participating in the study. The leaders will be allowed to ask questions and answers provided. Subsequently, radio talk shows on the local radio station (Busoga FM) will be conducted to provide information about the planned study to the wider community in the two districts.

***Recruitment:***

Once the sensitization activities are completed, trained Field Research Assistants (FRA) will approach households within each of the specified cluster communities, explaining the study to household members, and seeking consent to participate in screening for the trial. The FRA will seek a written pre-eligibility screening informed consent from adult members of the household. The FRA will then administer a short screening form to consenting adults to identify potential trial participants. In this first stage of screening, potential eligible household members would need to meet the following criterion:

1. current resident that have resided in their respective communities for at least 6 months prior to the date of screening
2. have no plans of migrating out of the study area over the next 12 months from the date of enrollment into the study,
3. aged 30 – 75 years,
4. currently not known to be pregnant
5. no previous diagnosis with diabetes, or diagnosis of diabetes of no longer than 12 months prior to the date of screening

The FRA will arrange an appointment with household members that meet the preceding criteria to return early morning on an agreed date, for a fasting plasma glucose (FPG) test. The FRA will return to the household on the agreed morning and conduct the FPG test usig an AccuCheck glucometer.

The FRA will arrange for a second FPG test for subjects with the first (Test 1) FPG results of at least 6.1 mmol/L (see Figure 3.7). Potentially eligible household members will those with a second (Test 2) FPG test result of at least 6.1 mmol/L, and will be referred to the respective cluster health facility for a confirmatory eligibility FPG test. The subjects will be given a referral note, and advised to report to the health facility within seven days, after an overnight fast on the day they report, with no exercise or smoking.

On presentation to their respective health facility with the referral note, a third FPG test will be conducted to confirm eligibility. Subjects will be classified as: a) normal if two of their FPG tests are less than 6.1 mmol/L, b) pre-diabetes if two of their FPG tests is between 6.1 and 6.9 mmol/L, and, c) diabetic if two of their FPG test results are greater than 6.9 mmol/L. Figure 12 and Table 12 summarizes the decision guide to identify eligible subjects in Uganda, as far as their FPG levels are concerned.

Figure 12: Algorithm for sequence of screening tests for Uganda


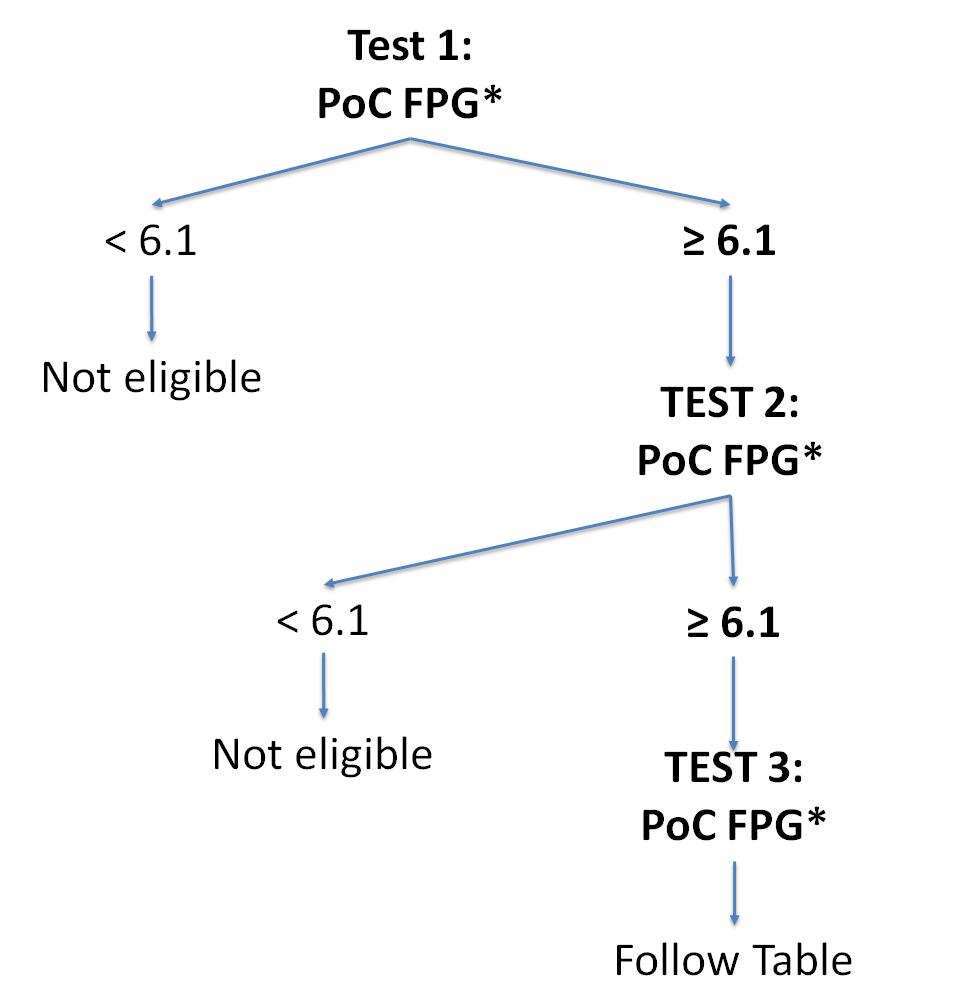


Table 12: Decision guide to enroll for Uganda

| **Test 1***  **(PoC FPG at home)** | **Test 2***  **(PoC FPG at home)** | **Test 3***  **(PoC FPG at health facility)** | **Outcome** |
| --- | --- | --- | --- |
| FPG = 6.1-6.9 | FPG ≥ 7.0 | FPG= 6.1-6.9 | Pre-D: RECRUIT |
|  |  | FPG ≥ 7.0 | T2D: RECRUIT |
|  | FPG= 6.1-6.9 | n/a | Pre-D: RECRUIT |
|  | FPG< 6.1 | Not eligible | DO NOT RECRUIT |
| FPG ≥ 7.0 | FPG > 7.0 | n/a | T2D: RECRUIT |
|  | FPG= 6.1-6.9 | FPG= 6.1-6.9 | Pre-D: RECRUIT |
|  |  | FPG ≥ 7.0 | T2D: RECRUIT |
|  | FPG < 6.1 | Not eligible | DO NOT RECRUIT |

*FPG values based on WHO recommendation[WHO 2006]

Following eligibility confirmation on the day they report for their FPG Test 3, an “Eligibility Confirmation Form” will be administered, and written informed consent obtained from the subject prior to enrollment into the trial. Briefly, a study staff at the health facility will provide information on the study objectives, study procedures, benefits from participating, risks of participating, and confidentiality issues will be emphasized using a written consent form document. Importantly, even while participating in the trial, participants will continue receiving standard medical care as needed, and as available at the health facility. Administration of any study procedures will only happen after the eligible subjects have consented to participate in the study. After a subject has consented to participate in the study, the subject will be enrolled with assignment of a unique study number and baseline evaluation conducted.

### 3.6.2 South Africa

**3.6.1.** **Community sensitization**

Community engagement will be implemented by sensitizing key stakeholders, incl. NGOs and health service providers within the Khayelitsha Eastern Sub-Structure (KESS). A series of meetings will be conducted in which the study team will provide information about the planned study to the stakeholders in the sub-Structure, including the KESS Director, local NGOs, and the managers of the selected study facilities. Study investigators will also conduct a community awareness raising campaign in partnership with the local branch of a National NGO to drive participant recruitment. The venue will be a central location which draws large numbers of visitors, such as a shopping mall located in close proximity to the study facilities.

Recruitment drivers will screen consenting adults to identify potential trial participants. They will conduct a random plasma glucose (RPG) test and refer eligible participants in line with the diagnostic criteria outlined in the Practical Approach to Care Kit (PACK) guidelines for primary care to the health facility to test for fasting plasma glucose (FPG) (Figure 3.2). Patients with an RPG ≥ 6.1 mmol/L will be advised to visit their health facility within a week, after an overnight fast on the day they report with no exercise or smoking.

**3.6.2.** **Facility recruitment**

Recruitment will take place at the two selected facilities. Persons presenting at the facilities who are either diagnosed with T2DM, or present with NCDs risk factors, including obesity, high blood pressure and/or impaired glucose tolerance, will be invited to participate in the trial. They will be recruited when visiting the facility for routine clinical care or upon referral from the community as per the above outlined protocol. Subjects will be classified as diabetic if two consecutive FPG tests > 6.9 mmol/L and as pre-diabetic if two consecutive FPG tests are between 6.1-6.9 mmol/L. A third FPG test may be needed to validate different test results (Figure 13 and Table 13).

Following eligibility confirmation, an ‘Eligibility Confirmation Form’ will be administered and written informed consent sought from the subject prior to study enrollment. Briefly, the study manager at the health facility will provide information on SMART2D study objectives, procedures, benefits and risks of participating, as well as confidentiality issues. Participants will receive a consent form and continue to receive standard medical care as needed, and as available at the health facility. Administration of any study procedures will only happen after eligible subjects have consented to participate in the study, upon which they will be enrolled and given a unique study number.

*Figure 13 Algorithm for sequence of screening tests for South Africa*


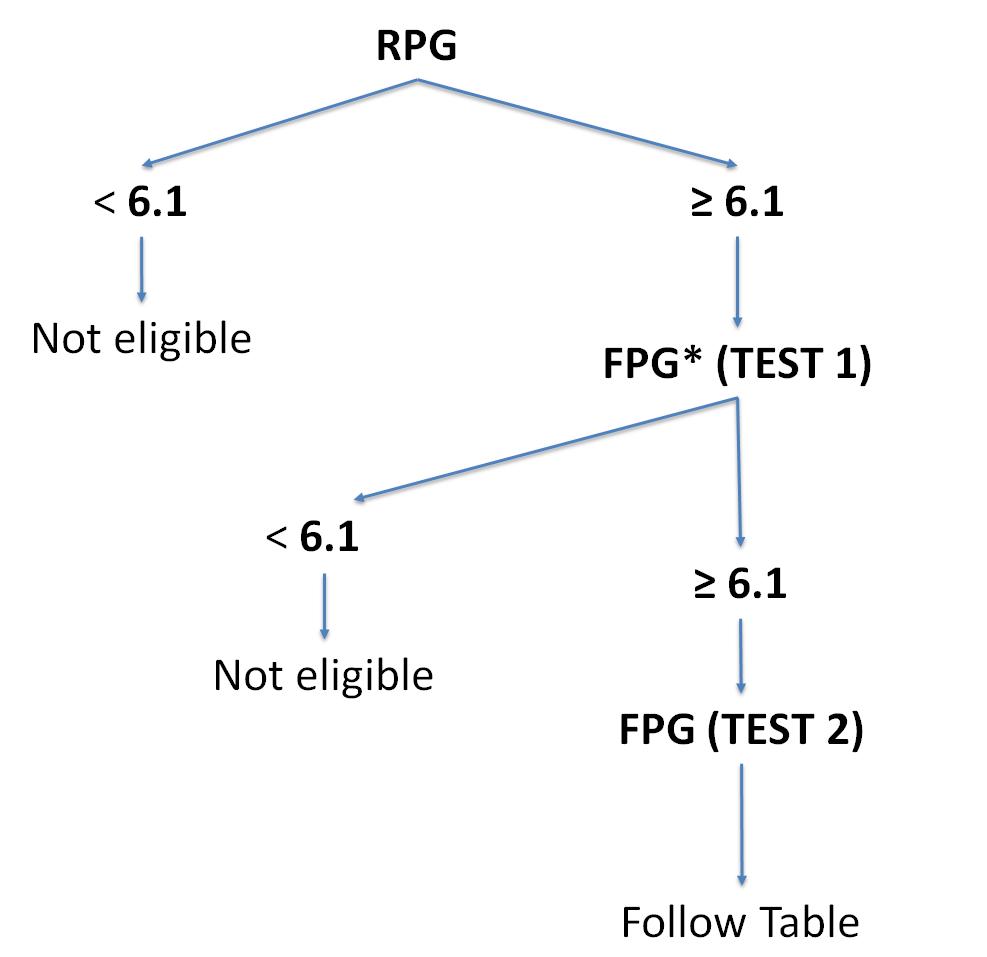


**FPG (TEST 2)**

**FPG (TEST 2)**

*Table 13: Decision guide to enroll for South Africa*

| **RPG**  **(mmol/L)** | **Test 1 (FPG)**  **(mmol/L)*** | **Test 2 (FPG)**  **(mmol/L)*** | **Outcome** |
| --- | --- | --- | --- |
| RPG ≥ 6.1 | FPG > 6.9 | FPG > 6.9 | T2DM (RECRUIT) |
|  |  | FPG= 6.1-6.9 | AT RISK (RECRUIT) |
|  | FPG = 6.1-6.9 | FPG= 6.1-6.9 | AT RISK (RECRUIT) |
|  |  | FPG < 6.1 | NOT ELIGIBLE |

*FPG values based on WHO recommendation [WHO 2006]

### 3.6.3 Sweden

In Sweden, since the suburbs chosen varied in terms of the composition of immigrant groups and the overall socio-economic status, the catchment area of each of the participating health centres were divided into zones using postal codes. These zones were randomly allocated to primary care versus integrated care, taking care to prevent contamination and spillover. Participants are recruited to the trial through community screening. The research team with input from health centres and municipalities have identified opportunities and spaces in the community to carry out screening activities. Both research staff as well as outreach workers from the municipalities are involved in mobilizing community members and inviting them to participate in the screening, by: 1) facilitating access to the target population through local NGOs, immigrant associations, religious bodies, such as churches and mosques, and cafes or other ventures owned by immigrant groups; 2) identifying and facilitating access to spaces to set up the screening activities such as shopping malls, local library, municipality hall or other public spaces such as swimming halls.

Community screening activities are carried out by the research team on weekdays and weekends using a combination of FINDRISC [J. Lindström et al. 2003], and hemoglobin A1c (HbA1c) test (both point-of-care and lab-based) using a recruitment algorithm summarized in Figure 14 and Table 14. All individuals with HbA1c values in the diabetes or pre-diabetes range are referred to the health centres to be registered and enrolled into the primary care only arm, or the integrated care arm as the case may be.

Figure 14: Algorithm for sequence of screening tests for Sweden


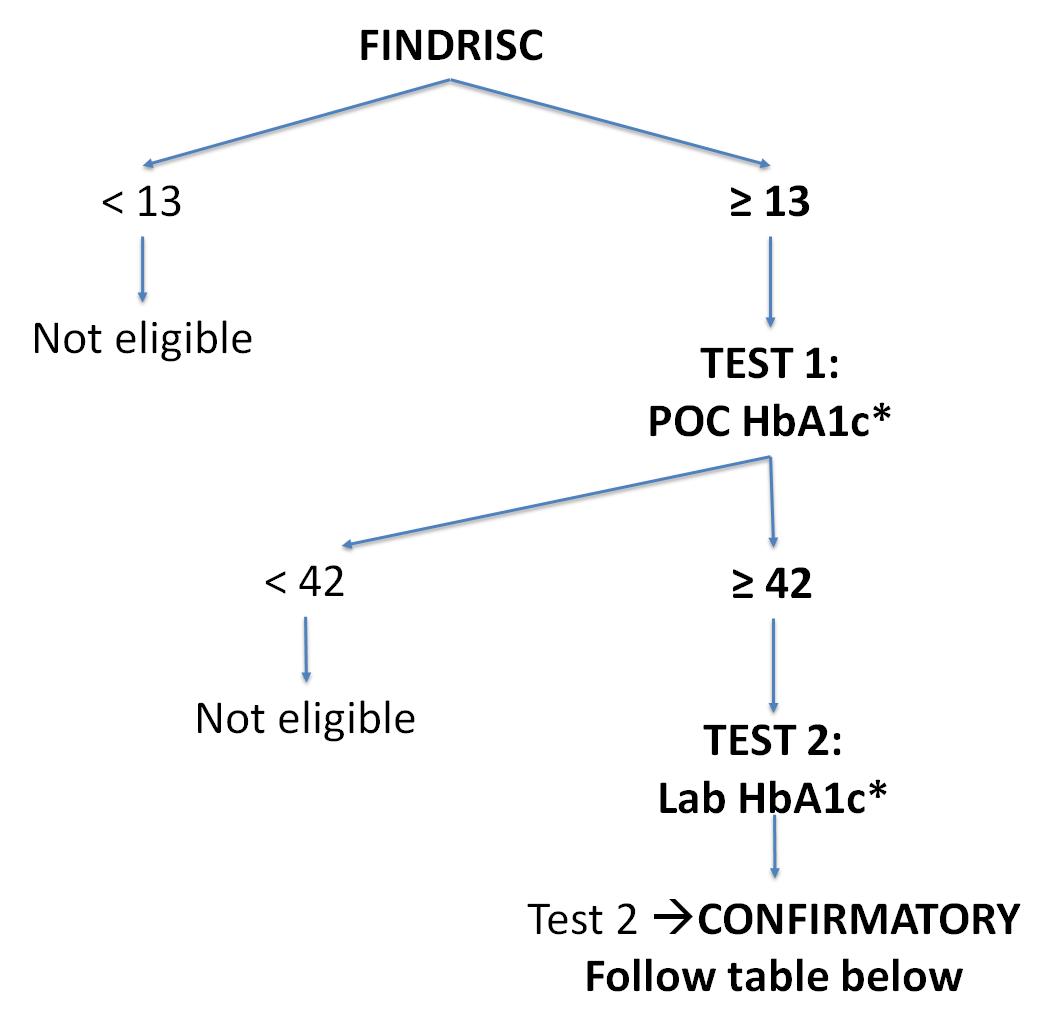


Table 14: Decision guide to enroll for Sweden

| **Test 1 (POC HbA1c)*** | **Test 2 (Lab HbA1c)*** | **Outcome** |
| --- | --- | --- |
| HbA1c = 42-47 | HbA1c = 42-47 | **Pre-D: RECRUIT** |
|  | HbA1c ≥ 48 | **T2D: RECRUIT** |
|  | HbA1c < 42 | NOT ELIGIBLE |
| HbA1c ≥ 48 | HbA1c ≥ 48 | **T2D: RECRUIT** |
|  | HbA1c = 42-47 | **Pre-D: RECRUIT** |

*HbA1c values based on WHO and ADA recommendation[WHO 2011; ADA 2016]

## 3.7 Baseline Evaluation:

At enrollment before baseline evaluation begins, and at all country sites, participants will be assigned a unique study number that will be used on all study forms. The study participant number will take the following format.

Country code

Facility/ cluster code

Participant

number

Study arm code

AA

**-**

#

**-**

#

#

#

#

#

**-**

The study number will be used to identify the study participant at each study visit, and will be used on all study data collection forms, on biochemical sample test requisition and results forms, as well as in the databases.

Following enrollment and study number assignment, participants in all the trial arms will under-go a baseline evaluation that will comprise the following:

1. Recording of the participant’s contact information to be used to contact the participant in case they fail to report back to the health facility for their clinic and/or study visits,
2. A background information that will collect demographic and social characteristics
3. Behavioral risk factor characteristics (physical activity, tobacco and alcohol use history, and dietary behavior assessment).
4. Medical history assessment
5. Physical measurements (weight, height, waist circumference and blood pressure),
6. Biochemical measurements including HbA1c and FPG (Uganda & South Africa only)
7. A 7-day pedometer steps assessment
8. Participant diabetes knowledge
9. Illness perception
10. Social support and sources of support
11. Self-efficacy, autonomy support & self-regulation
12. Psychological adjustment
13. Quality of life & Stress
14. Adherence to study procedures and to intervention elements

***Facility baseline evaluations:***

We will conduct baseline facility evaluations that will include:

1. Health facility assessment on the level of staffing with different health care cadres, equipment and supplies, and availability of guidelines relevant for diabetes care
2. A context assessment to assess the opinion and views of facility health care workers regarding facilities in their working environment

***Community evaluations:***

Furthermore as part of baseline evaluation, we will conduct community environment evaluation (EPOC)

## 3.8 Follow-up evaluations:

Each participant will be followed up to 12 months from the time of enrollment, or until his/her death or loss to follow-up. Active data collection will occur at baseline and months 3, 6 and 12. However participants with diabetes will return to their respective cluster health facilities monthly for their clinic visits and drug refills.

During follow-up study visits, we will conduct the following assessments:

1. Medical history assessment
2. Clinical evaluation of participants to assess any changes in clinical participants markers and outcomes
3. Physical measurements (weight, waist circumference and blood pressure),
4. Biochemical measurements including HbA1c and FPG (Uganda & South Africa only)
5. 7-day pedometer steps assessment
6. Participant diabetes knowledge
7. Illness perception
8. Social support and sources of support
9. Self-efficacy, autonomy support & self-regulation
10. Psychological adjustment
11. Quality of life & Stress
12. Diabetes treatment satisfaction
13. Adherence to study procedures and to intervention elements
14. Out of pocket expenditure and incremental cost on the system
15. Incremental cost to the health facility
16. Process evaluation

***Process Evaluation***: In any trial, a high degree of compliance to the intervention elements is necessary to minimize decrease in statistical power and obtain unbiased results. In the case of non-compliance that is associated with both intervention element(s) and the study outcomes of interest, there is a particularly serious threat to validity. However, the best method for ensuring full compliance is to monitor compliance with the trial processes. Thus, participants will receive encouragement at their monthly visits to take their medications at the same time every day, and identify a Care Companion to provide medication reminders, as well as healthy lifestyle adherence. Research nurses will assess compliance in three ways: direct questioning and pill count assessed at each monthly visit, and biochemical assessment of plasma glucose. Research nurses will also make an effort to identify and address barriers to compliance.

Table 15 summarizes the schedule of study procedures and tools to be applied.

Table 15: Schedule of study procedures

| **Study Activity** | | **-7 to 0 days** | **BASELINE** | **M3** | **M6** | **M12** |
| --- | --- | --- | --- | --- | --- | --- |
| **I. Consent forms** | Screening consent | X |  |  |  |  |
|  | Study informed consent |  | X |  |  |  |
|  |  |  |  |  |  |  |
| **II. Enrollment & Study ID** | Study ID number assignment |  | X |  |  |  |
|  | Patient registration in Register |  | X |  |  |  |
|  | Patient file creation |  | X |  |  |  |
|  | Patient appointment card |  | X |  |  |  |
|  |  |  |  |  |  |  |
| **III. Physical Measurements** | Height |  | X |  |  |  |
|  | Weight |  | X | X | X | X |
|  | Waist circumference |  | X |  | X | X |
|  | Blood pressure |  | X | X | X | X |
|  | 7-day pedometer steps |  | X |  |  | X |
|  |  |  |  |  |  |  |
| **IV. Biochemical** | FPG (Uganda & South Africa) | X, X | X | X | X | X |
|  | HbA1c (All sites) |  | X |  |  | X |
|  |  |  |  |  |  |  |
| **V. Basic participant data** | Pre-eligibility assessment | X |  |  |  |  |
|  | Eligibility confirmation |  | X |  |  |  |
|  | Eligibility and recruitment check |  | X |  |  |  |
|  | Contact Information |  | X |  |  |  |
|  | Demographic and social measures |  | X |  |  |  |
|  | Medical and medication history |  | X |  |  |  |
|  | Behavioral measures (diet, physical activity, foot care, tobacco use, alcohol consumption) |  | X |  |  | X |
|  |  |  |  |  |  |  |
| **VI. Intervention process measures** | Participant diabetes knowledge |  | X |  |  | X |
|  | Illness perception |  | X |  |  | X |
|  | Social support and sources of support |  | X |  |  | X |
|  | Self-efficacy, autonomy support & self-regulation |  | X |  |  | X |
|  | Psychological adjustment |  | X |  |  | X |
|  | Quality of life & Stress |  | X |  |  | X |
|  | Diabetes treatment satisfaction |  |  |  |  | X |
|  | Out of pocket expenditure |  |  | X | X | X |
|  | Incremental cost to the health facility |  |  | X | X | X |
|  | Adherence to study procedures and to intervention elements |  | X |  |  | X |
|  |  |  |  |  |  |  |
| **VII. Facility forms** | Process evaluation |  |  | X | X | X |
|  |  |  |  |  |  |  |
| **VIII. Community forms** | Environment context measurement (EPOC) |  | X |  |  |  |

## 3.9 Outcomes of interest:

*Primary outcomes of interest*

Among participants with pre-diabetes, the primary outcome of interest will be reduction in plasma glucose between baseline and month 12 of follow-up. We hypothesize that participants with pre-diabetes in the facility + community arm will on average have a higher reduction in their HbA1c reading by the end of follow-up compared to participants with pre-diabetes in the facility alone arm, and the current practice arm in Uganda.

Regarding participants with type 2 diabetes, research has shown that, reduction of HbA1c level by 1% among such individuals leads to: a) 19% less likelihood of cataracts, b) 16% less likelihood of heart failure, and c) 43% less likelihood of amputations or death due to peripheral vascular disease [Anon 1993]. Further the UK Prospective Diabetes Study (UKPDS) [King et al 1999], and the Diabetes Control and Complications Trial (DCCT) demonstrated that improving HbA1c by 1% for people with type 1 diabetes *or* type 2 diabetes cuts the risk of microvascular complications by 25%. Therefore among participants with diabetes, the primary outcome of interest will also be reduction of their plasma glucoce levels. A participant will be classified as having had their plasma glucose reduced if:

1. Their HbA1c reading at month 12 is reduced to below 53 mmol/mol, or
2. Their HbA1c reading at month 12 is 5 mmol/mol below their baseline reading.

*Secondary outcomes of interest*

Secondary outcomes in this trial will include:

a) Incidence of diabetes among participants with pre-diabetes

b) Incidence of conversion from a state of pre-diabetes to normal plasma glucose levels.

c) Incidence of adverse events, including hospitalizations due to hypo- or hyperglycemia, or complications of diabetes.

d) Behavioral outcomes, including diet, physical activity, foot care, tobacco and alcohol consumption.

e) Costs of implementation of the intervention elements

f) Participant satisfaction with the individual intervention elements, including self-management

g) Differences between the country sites, in regard to baseline context infrastructure for prevention and management of T2D

h) Degree of implementation of intervention elements in each country, and differences between country sites informed by site-specific process evaluations

i) Effect of the interventions on perceptions towards diabetes, life style measures including physical activity, dietary behavior, etc.

j) Differences between the country sites, and changes within these contexts, as a result of implementing the intervention elements.

## 3.10 Sample size Determination

Although the overall aim during data analysis will be to combine participants across all the three country sites, it is recognized that: a) there will most likely be baseline differences across the country sites reflecting the different population characteristics between the countries; and, b) each site will want to analyze data from their site-specific arms. Thus the sample was calculated such that differences between the trials arms in regard to primary outcomes can be detected at each study site separately when desired.

The sample size for this trial has been determined based on the primary hypothesis that by the end of follow-up, the “facility plus community” arm will be superior to the “facility alone” arm, and the current practice arm in Uganda, with respect to: a) mean HbA1c readings among participants with pre-diabetes, and b) the proportion of participants with diabetes with controlled blood sugar levels.

*Sample size for participants with pre-diabetes*

With the aim of being able to detect an average difference between any two trial arms, in mean HbA1c by month 12 of at least 3 mmol/mol (effect size), with a standard deviation of 2.5 mmol/mol in the intervention arm, and a standard deviation of 2.5 mmol/mol in the comparison (control) arm; the required sample size before adjusting for clustering, at an 80% power and 5% level of significance is 34 participants per arm. For this first stage calculations, we used the formula as described in Hayes and Bennett (1999) [Hayes et al. 1999] for a continuous outcome:

$$Initial sample size(n_{i})=\left[ \frac{\left( Z_{\alpha/2}+Z_{\beta} \right)^{2}*\left( \sigma_{0}^{2}+\sigma_{1}^{2} \right)}{\left( \mu_{0}-\mu_{1} \right)^{2}} \right]$$

where: $Z_{\alpha/2}$ = standard normal distribution value at $\alpha$ level of significance

= standard normal distribution value at level of significance


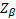

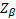

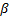

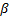


$\alpha$ = level of significance

= type 2 error, for which (1-$\beta$) is the power of the study to detect the desired effect size


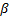

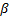


$\sigma_{0}^{2}$ = variance of outcome variable (HbA1c) at month 12 in the comparison (control) arm

$\sigma_{1}^{2}$ = variance of outcome variable (HbA1c) at month 12 in the intervention arm

$\mu_{1}$ = mean value of outcome variable (HbA1c) at month 12 in the intervention arm

$\mu_{0}$ = mean value of outcome variable (HbA1c) at month 12 in the comparison (control) arm

$\left( \mu_{0}-\mu_{1} \right)$ = minimum effect size to be detected


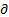

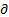


The necessary sample size for a cluster randomized trial depends on the variation in the intervention effects between the arms, and the clustering due to trial arm allocation (which reduces the variance of the difference between the arms), and the correlation between the repeated observations in each participant. Given that we have a fixed number of health facilities (clusters), to adjust for clustering we used a formula for fixed number of clusters and fixed number of participants within clusters as described in Hemming et al (2011) page 19 [Hemming et al. 2011].

$$Sample size per cluster=\frac{n_{i}*k*\left( 1-\rho\right)}{\left( k-n_{i}*\rho\right)}$$

where: $n_{i}$ = initial sample size

$k$ = number of clusters

= inter-cluster correlation


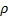

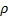


It is important to note that each site will be using different numbers of clusters; specifically Uganda will be working in 9 clusters, Sweden in 4 clusters, whereas South Africa will be working in 2 clusters. Thus different inter-cluster correlation (ICC) coefficient values are expected. We were unable to find published literature providing estimates for ICC coefficients for HbA1c among persons with pre-diabetes. We therefore explored various values of ICC coefficients for each site, and used values that provided the highest sample sizes to detect the desired effect size. For Uganda we used an ICC value of 0.200 to obtain a sample size of 112 participants, for South Africa we used an ICC value of 0.044 to obtain a sample size of 129, whereas for Sweden we used an ICC value of 0.06 to obtain a sample size of 79; all per study arm. For Sweden, there will be a further interest to compare the primary outcome of interest between immigrant participants of non-European origin versus immigrant participants of European origin. In order to allow for this sub-analysis, the sample size of 79 for Sweden was doubled to 158 to enable detection of the same effect size between immigrant participants of non-European origin versus immigrant participants of European origin.

Further, we project a loss to follow-up of up to 10% over the 12 months follow up, thus the sample sizes obtained were accordingly adjusted upwards by 10%. Table 16 (a) provides a summary of the estimates used, and the sample size estimates obtained.

Table 16 (a): Parameter value estimates used in sample size calculations for participants with pre-diabetes

| **Parameter** | **Estimate** | | |
| --- | --- | --- | --- |
| Level of significance (α) | 5% | | |
| Power (1-β) | 80% | | |
| Primary outcome of interest | Mean change in HbA1c reading | | |
| Standard deviation (σ), of mean change in HbA1c | 2.5 | | |
| Minimum anticipated difference between arms, in primary outcome by month 12 | $\left( \mu_{0}-\mu_{1} \right)$ =  5 mmol/mol | | |
| Required sample size per arm, before adjusting for clustering (*n_i_*) | 34 | | |
|  | Uganda | South Africa | Sweden |
| Number of clusters | 9 | 2 | 4 |
| Inter-cluster correlation (ρ) | 0.200 | 0.044 | 0.070 |
| Sample size per arm, after adjusting for clustering (n) | 112 | 129 | 158 |
| Sample size per arm, after further adjusting for estimated loss to follow-up of 10% | 124 | 142 | 174* |

* *For Sweden, at least 87 participants with pre-diabetes will need to be immigrants of non-European origin, and at least 87 participants with pre-diabetes will need to be immigrants of European origin*

*Sample size for participants with diabetes*

A prospective cohort study among patients with diabetes in the United Kingdom showed that people with type 2 diabetes who reduce their HbA1c level by 1% are: a) 19% less likely to suffer cataracts, b) 16% less likely to suffer heart failure, and c) 43% less likely to suffer amputation or death due to peripheral vascular disease [Stratton et al. 2000]. Therefore among participants with diabetes, we aim at reducing their plasma glucose levels by month 12. A participant with diabetes will be defined as having had their plasma glucose reduced if:

1. Their HbA1c reading at month 12 is reduce to below 53 mmol/mol, or
2. Their HbA1c reading at month 12 is 5 mmol/mol below their baseline reading.

Thus we will aim at maximizing the proportion of participants with diabetes whose blood sugar levels has been reduced, per the criteria defined above. We aim at being able to detect a difference of at least 2% in the percentage of participants with diabetes with reduced plasma glucose levels by month 12 between the trial arms (effect size). The required sample size before adjusting for clustering, at 80% power and 5% level of significance is 58 participants per arm. We have also assumed that in the intervention arm we shall attain reduction in plasma glucose per the criteria given above, in 30% of participants with diabetes (P_1_= 0.3). For this first stage calculations, we used the formula as described in Hayes and Bennett (1999) [Hayes 1999] for proportions, that is:


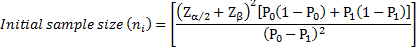


where: $n_{i}$ = initial sample size (58)

$P_{0}$ = Proportion of participants with diabetes in the comparison (control) arm, whose plasma glucose levels are controlled by month 12 (32%)

$P_{1}$ = Proportion of participants with diabetes in an intervention arm, whose plasma glucose levels are controlled by month 12 (30%)

For an estimate of inter-cluster correlation (ICC) among individuals with diabetes, we found only two publications of cluster randomized trials reporting ICC estimates among diabetes patients, one by Littenberg et al (2006) in which they report an inter-cluster correlation (ICC) coefficient for HbA1c among patients with diabetes of 0.055 [Littenberg et al. 2006]; and another by Singh et al (2015) in which they report an ICC coefficient of 0.091 [Singh et al. 2015]. We use some of these estimates. For Uganda we used an ICC value of 0.0.091 to obtain a sample size of 128 participants, for South Africa we used an ICC value of 0.020 to obtain a sample size of 136, whereas for Sweden we also used an ICC value of 0.020 to obtain a sample size of 81; all per study arm.

Once again for Sweden, there will be a further interest to compare the primary outcome of interest between immigrant participants of non-European origin versus immigrant participants of European origin. In order to allow for this sub-analysis, the sample size of 81 for Sweden was doubled to 162 to enable detection of the same effect size immigrant participants of non-European origin versus immigrant participants of European origin.

Similarly, we project a loss to follow-up of up to 10% over the 12 months follow up, thus the sample sizes obtained were accordingly adjusted upwards by 10%. Table 16 (b) provides a summary of the estimates used, and the sample size estimates obtained.

Table 16 (b): Parameter value estimates used in sample size calculations for participants with diabetes

| **Parameter** | **Participants with Diabetics** | | |
| --- | --- | --- | --- |
| Level of significance (α) | 5% | | |
| Power (1-β) | 80% | | |
| Primary outcome of interest | % of participants with reduced plasma glucose | | |
| Proportion of participants with reduced HbA1c by month 12 in control arm ($P_{0}$) | 30% | | |
| Minimum anticipated difference between arms, in primary outcome by month 12 | $\left( P_{0}-P_{1} \right)$ = 2% | | |
| Required sample size per arm, before adjusting for clustering (*n_i_*) | 58 | | |
|  | Uganda | South Africa | Sweden |
| Number of clusters | 9 | 2 | 4 |
| Inter-cluster correlation ($\rho$) | 0.091 | 0.020 | 0.020 |
| Sample size per arm, after adjusting for clustering (n) | 128 | 136 | 81 |
| Sample size per arm, after further adjusting for estimated loss to follow-up of 10% | 141 | 150 | 90 |

* *For Sweden, at least 90 participants with diabetes will need to be immigrants of non-European origin, and at least 90 participants with diabetes will need to be immigrants of European origin*

Figure 15 summarizes the trial and the sample sizes to be used at each site per study arm.

Figure 15: Schema for the trial

^ф^265 = 124 with pre-diabetes, & 141 with diabetes

^€^292 = 142 with pre-diabetes, & 150 with diabetes

^¥^353 = 174 with pre-diabetes (87 immigrants of European origin plus 87 immigrants of non-European origin),

& 179 with diabetes (90 immigrants of European origin plus 90 immigrants of non-European origin)

Overall total participants

N=1997

Uganda

n=795

South Africa

n=584

Sweden

n=706

Follow-up

Month 3

Month 6

Month 12

Baseline evaluation

Facility only

n=292^€^

Facility+ community

n=292

Facility only

n=353^¥^

Facility+ community

n=353

Facility only

n=265^ф^

Facility+ community

n=265

Control

n=265

Randomization

## 3.11 Quality Control Measures

PROVIDER TRAINING: As a first step in our patient-centered approach, all clinical staff with patient contact will undergo a health care provider training that will last 3 – 5 days depending on the site. The goal of this training will be to standardize the care provided, raise awareness of inadvertent responses that could be stigmatizing to participants, and of possible self-stigmatization among patients, especially when the effects could restrict access to family and community support. Study clinicians and nurses will be trained in adherence counseling; they will be taught strategies for reducing patient risk of non-adherence, identifying and prioritizing problems leading to non-adherence, and building patient self-efficacy for problem-solving and emotional support.

MINIMIZING LOSS TO FOLLOW UP: We will make every effort to minimize loss to follow-up. As part of an incentive for continued participation, participants will have access to the health facility clinics on all working days of the week (Monday to Friday), even outside of their scheduled visits, for the duration of the study period. Participants will be encouraged to use the facility clinic any time they require medical attention. All participants will receive care and services in accordance with standard medical practice available at the health facility.

Further, in our experience it is most helpful to know the participants’ telephone contact information, and the exact location of each participant's residence. This enables study staff to contact participants who miss a clinic appointment, and conduct home visits for participants who miss a clinic appointment by more than one week. Thus a detailed description of each participant's contact information will be recorded at the baseline visit; and this will facilitate future home visits (if necessary). We will collect information on landmark buildings on the way to a participant’s home, the name of the participant’s local community leader, and the participant’s street address (when available). At the time of enrollment, we will ask each participant’s permission to conduct home visits. Regular checks will be made during the follow-up period to determine any residency changes. However, despite our efforts to track participants, some will move without notifying us or will provide an inaccurate address. In these cases, we will pay regular visits to each participant’s home and talk with relatives, neighbors, and friends to determine the participants’ whereabouts. These procedures will be conducted in a culturally sensitive manner without disclosure of information on the participant’s health status or study participation.

ADHERENCE TO STUDY INTERVENTION ELEMENTS: Adherence counseling will occur during the health education and motivational couching sessions that participants will be encouraged to attend. The general health education and motivational couching sessions will occur in morning hours of every clinic day. Further one-on-one health education and motivational couching will be conducted during interactions between the participant and health care workers. Further, treatment adherence counseling will be provided by the pharmacy assistant or adherence nurse-counselor that will include the following: (1) the importance of diabetes treatment adherence, including the adherence level necessary for good treatment outcomes; (2) methods of monitoring treatment outcomes to optimize patient progress; and (3) the patient’s beliefs about the effects of diabetes treatment. The counselor will discuss lifestyle issues (ranging from physical activity, alcohol and tobacco use and diet), explore possibilities for incorporating diabetes and/or lifestyle modification into the participant’s daily schedule, and determine the participant’s motivation and expectations for seeking treatment. Participants will be asked to think about these issues and prepare any questions for their next counseling session. During visits subsequent to treatment, and/ or lifestyle modification initiation, counselors will summarize information presented during previous visits and assess the participant’s confidence in and commitment to long-term diabetes treatment , and/ or healthy lifestyle compliance (using a rating scale of low, moderate, and high). Furthermore for participants with diabetes, the counselor will explore strategies for ensuring that each patient is supported in their home environment while receiving treatment, with encouraging and helping the patient identify a “Care Companion”.

At each health facility, the study staff will help with the establishment of a peer support group for participants with diabetes, that will comprise of patients with diabetes, with a peer group leader being an “expert diabetes patient”. The progress and activities of the peer support groups will be monitored over the course of the study, with support from the study team where needed.

## 3.12 Data Management Plan:

### 3.12.1 Data collection tool development plans

To ensure consistency and minimization of errors in the data collected, standardized methods of data management will be used across the trial sites. This will start with development of common data collection tools to be administered at each of the trial sites. The tools will contain the common elements to be administered at all sites, and elements that may be administered by one or two sites only. Prior to the tools being adopted, Site Leaders and their respective research team members will discuss each tool and agree on the minimum set of common questions/ data items to be collected at all the three sites. Each site may thereafter add any site-specific questions of interest.

### 3.12.2 Database development plans

Once the content of the data collection tools has been agreed upon and finalized; common data entry formats will be developed in an appropriate data management and easy to use software, e.g. REDCap or EPIDATA. The data entry formats will then be sent to the three study sites, and pilot testing of these data entry formats conducted using pilot/ pre-test data. Should a given site opt to use a different software, they will do so but the variable names and their formatting will be maintained at all sites to enable quick merging of the data at the end of the trial. Any required corrections will be made before initiation of the main trial activities. This approach will ensure that data from each site can easily be merged to perform overall intervention analysis that includes data from all the three sites.

### 3.12.3 Data management

As a standard operating procedure, completed questionnaires and other data collection tools will be checked by designated data collection supervisors at each site, for missed entries, inconsistencies and any other obvious errors on the completed tools. In case errors are identified, the responsible data collection personnel will attempt to correct, and/or contact participant where possible. Once the completed tools have been checked and cleared by the respective data collection supervisor, they will be logged with the data entry supervisor for data entry.

Except when a site opts to use electronic data capture methods, for example using tablets, all on completed questionnaires and other data collection tools will be double entered (by two different data entry personnel), to minimize data entry errors. Variance in values of the data entered will be periodically generated (at least once a week), and the errors corrected. Each site will be responsible for ensuring that data cleaning is conducted at adequate frequency.

## 3.13 Statistical analysis Plan

### 3.13.1 Primary intervention efficacy analysis:

We will use intention-to-treat analyses for all primary outcomes, that is, all participants will be analyzed as assigned to their respective study arms. Since the unit of analysis for the primary outcome of interest are the individual participants, to minimize the effect of within cluster correlations, we will employ generalized estimating equations (GEEs) to evaluate changes over time in HbA1c values as a continuous variable among participants with pre-diabetes; and the proportion of participants with stabilized plasma glucose among participants with diabetes, in relation to assigned trial arm. Thus we use multivariable linear regression mixed effects modeling with a random intercept, unstructured correlation matrix and robust standard errors, to evaluate changes over time in HbA1c, and logistic regression analysis to compare the proportion of participants with stabilized plasma glucose among participants with diabetes.

Missing data are expected due to missed appointments, drop-outs, loss-to-follow-up and/or death. One technique to deal with missing data when the outcome is continuous, is hierarchical linear modeling (HLM; also called random effects regression) [Singer et al. 2003].

We do not anticipate differences in the intercept within each site, as randomization is expected to balance out baseline differences between the groups. However, there may be differences in the intercepts between sites, reflecting differences within the client populations in each site. We anticipate that the primary factor affecting the slope (i.e., the rate of change of the outcome measure) will be group membership (i.e, facility only or facility plus community intervention). If there is any significant unexplained variance after baseline differences and site membership have been accounted for, we can look for other factors influencing the slope, such as sex, age, co-morbidities, etc. Another analytic option that will be considered is generalized estimating equations (GEE) [Zeger et al. 1986], which can adjust for clustered, serially correlated data with missing values. The choice of techniques will be based on the nature of the data.

The analysis plan will combine the study groups across sites. However, it may turn out that the demographic characteristics of participants and available treatments differ so widely from site to site that this will not be possible. In that case, it will be necessary to compare each treatment group to its site-specific study groups.

### 3.13.2 Analyses of secondary objectives

1. For the "incidence of progression from pre-diabetes state to diabetes state", we will use Kaplan Meier methods to compare incidence between the two arms. The log-rank test will be used and Kaplan-Meier curves will be plotted for each treatment group.
2. Adverse events, including hospitalizations due to hypo- or hyperglycemia, morbidity. For adverse events outcomes, we must account for the possibility that participants may experience multiple episodes over the study period. Thus, we will use log-binomial models for repeated outcomes to examine the treatment effect on these outcomes
3. For costs outcomes, cost evaluation will be undertaken from the health program provider perspective. The health service use costs of the two strategies will be calculated at 12 months in both arms. Costs incurred between baseline and 12 months will be discounted at ??%. The mean cost of the two groups over 12 months will be compared using non-parametric tests. Results will be reported with 95% confidence intervals and uncertainty will be examined using sensitivity analysis. An incremental cost-effectiveness ratio will be calculated over the 12 months of the intervention. The need or otherwise for cost-effectiveness modeling will depend on there being economically important clinically significant differences in glycaemic control, as determined by the likely impact on and hence, long-term effects on morbidity and mortality. Whether any differences in glycaemic control are important will be informed by reviewing the cost-effectiveness of other interventions that have been evaluated using lifetime models. If such differences do exist, the modeling of the longer-term effects to assess the incremental cost per quality adjusted life-year will be conducted
4. For comparison of differences between the country sites, in regard to baseline context infrastructure situation for prevention and management of diabetes, these are baseline comparisons thus for continuous context variables, we will use the t-test statistic or the equivalent non-parametric test. For categorical context variables we will use the chi-square test statistic.
5. Similarly, for the degree of implementation of the intervention elements in each country, and differences between the country sites, we will compare the process evaluation variables/ indicators regarding ways in which program services and goods are provided (i.e. deviations) using the t-test statistic or the equivalent non-parametric test. For categorical context variables we will use the chi-square test statistic.
6. To compare changes in participant perceptions towards diabetes, physical activity, dietary behavior; and changes within contexts, as a result of implementing the intervention elements; we use multivariable linear regression mixed effects modeling with a random intercept, unstructured correlation matrix and robust standard errors, to evaluate changes over time in these outcomes of interest in relation to the assigned study arm. Visit month (categorical) at which the outcome is measured, study arm would be included in the model as fixed effects. The null hypothesis to be tested in this analysis would be that the mean changes at all the post-baseline visit months are equal between the treatment groups. The alternative hypothesis would be that the mean changes are not equal at, at least one post-baseline visit month.

We recognize that since sample size calculations for this trial were not based on the secondary outcomes, lack of significant differences in analysis of secondary outcomes might either be true lack of differences in these outcomes, or due to the fact that the trial was not sufficiently powered to detect differences in these outcomes. Thus interpretation of findings on secondary outcomes will be done with caution in case we do not detect any differences.

## 3.14 Ethical Considerations

Approval for conduct of this trial will be sought, and research ethical oversight provided by the Institutional Review Boards (IRB) at the respective trial sites; that is, the Higher Degrees, Research and Ethics Committee (HDREC) of Makerere University School of Public Health in Uganda, the Senate Research Committee of the University of the Western Cape in South Africa, and the Regional Ethical Board in Stockholm, in Sweden. In Uganda, further approval will be sought from the Uganda National Council for Science and Technology (UNCST).

We will seek written informed consent, making sure that each eligible subject understands that participation in the trial is voluntary, and that he/she may withdraw participation at any time, even after consent is granted. Thus Field Research Assistants (FRA) will review to eligible subjects, the trial objectives, procedures and expectations of the trial from any enrolled participant; prior to enrollment in the trial. Eligible subjects will then be provided with the opportunity to voluntarily decide whether to provide consent to participate or not. The consent process will be completed with the subject providing their signature, or a witnessed thumb-print on the consent document.

To ensure confidentiality of trial participant data, completed data tools will only bear the participant study number (no names or locator information), and will only be handled by authorized study personnel. The names of participants will only appear on the consent documents, and their locator information on the Contact Informtion form. These participant identifying documents will be stored separately from the rest of the databases. All data forms will be kept in a lockable office space/drawer that is accessible to only authorized trial supervisors and project coordinators at each respective trial site.

# 4.0 References:

ADA, 2016. 2015 American Diabetes Association (ADA) Diabetes Guidelines. , pp.1–46. Available at: http://www.ndei.org/uploadedFiles/Common/NDEI/Treatment_Guidelines/ADA 2015 Summary PDF.pdf [Accessed May 16, 2016].

ADA, 2013. Economic costs of diabetes in the U.S. in 2012. *Diabetes care*, 36(4), pp.1033–46.

Alvarsson, M., Hilding, A. & Ostenson, C.-G., 2009. Factors determining normalization of glucose intolerance in middle-aged Swedish men and women: a 8-10-year follow-up. *Diabetic medicine : a journal of the British Diabetic Association*, 26(4), pp.345–53.

Anon, 1993. The effect of intensive treatment of diabetes on the development and progression of long-term complications in insulin-dependent diabetes mellitus. *N Engl J Med*.

Apoc, 2007. African Programme for Onchocerciasis Control: Revitalising health care delivery. *Development*, pp.1–36.

Atun, R., Jaffar, S., Nishtar, S., Knaul, F.M., Barreto, M.L., Nyirenda, M., et al., 2013. Improving responsiveness of health systems to non-communicable diseases. *Lancet*, 381(9867), pp.690–7.

Bertram, M.Y., Jaswal, A.V.S., Van Wyk, V.P., Levitt, N.S. & Hofman, K.J., 2013. The non-fatal disease burden caused by type 2 diabetes in South Africa, 2009. *Global health action*, 6, p.19244.

Binagwaho, A., Nutt, C.T., Mutabazi, V., Karema, C., Nsanzimana, S., Gasana, M., et al., 2013. Shared learning in an interconnected world: innovations to advance global health equity. *Globalization and health*, 9, p.37.

Burstrom, B., 2009. Will Swedish healthcare reforms affect equity? *BMJ (Clinical research ed.)*, 339, p.b4566.

Caro, J.J., Ward, A.J. & O’Brien, J.A., 2002. Lifetime costs of complications resulting from type 2 diabetes in the U.S. *Diabetes care*, 25(3), pp.476–81.

Chalker, J.C., Wagner, A.K., Tomson, G., Johnson, K., Wahlström, R. & Ross-Degnan, D., 2013. Appointment systems are essential for improving chronic disease care in resource-poor settings: learning from experiences with HIV patients in Africa. *International health*, 5(3), pp.163–5.

Dalal, S., Beunza, J.J., Volmink, J., Adebamowo, C., Bajunirwe, F., Njelekela, M., et al., 2011. Non-communicable diseases in sub-Saharan Africa: what we know now. *International journal of epidemiology*, 40(4), pp.885–901.

Decroo, T., Van Damme, W., Kegels, G., Remartinez, D. & Rasschaert, F., 2012. Are Expert Patients an Untapped Resource for ART Provision in Sub-Saharan Africa? *AIDS research and treatment*, 2012, p.749718.

Decroo, T., Rasschaert, F., Telfer, B., Remartinez, D., Laga, M. & Ford, N., 2013. Community-based antiretroviral therapy programs can overcome barriers to retention of patients and decongest health services in sub-Saharan Africa: a systematic review. *International health*, 5(3), pp.169–79.

Diabetes UK, 2009. Improving supported self-management. , (November). Available at: https://www.diabetes.org.uk/Documents/Reports/Supported_self-management.pdf [Accessed August 24, 2015].

Distiller, L.A., Brown, M.A., Joffe, B.I. & Kramer, B.D., 2010. Striving for the impossible dream: a community-based multi-practice collaborative model of diabetes management. *Diabetic medicine : a journal of the British Diabetic Association*, 27(2), pp.197–202.

Duwell, M.M., Knowlton, A.R., Nachega, J.B., Efron, A., Goliath, R., Morroni, C., et al., 2013. Patient-nominated, community-based HIV treatment supporters: patient perspectives, feasibility, challenges, and factors for success in HIV-infected South African adults. *AIDS patient care and STDs*, 27(2), pp.96–102.

Eldridge, S.M., Ashby, D. & Kerry, S., 2006. Sample size for cluster randomized trials: effect of coefficient of variation of cluster size and analysis method. *International journal of epidemiology*, 35(5), pp.1292–300.

Eriksson, K.F. & Lindgärde, F., 1991. Prevention of type 2 (non-insulin-dependent) diabetes mellitus by diet and physical exercise. The 6-year Malmö feasibility study. *Diabetologia*, 34(12), pp.891–8.

Fukui, M., Tanaka, M., Toda, H., Senmaru, T., Sakabe, K., Ushigome, E., et al., 2011. Risk factors for development of diabetes mellitus, hypertension and dyslipidemia. *Diabetes research and clinical practice*, 94(1), pp.e15-8.

Gåfvels, C. & Wändell, P.E., 2007. Coping strategies in immigrant men and women with type 2 diabetes. *Diabetes research and clinical practice*, 76(2), pp.269–78.

Guideline Committee, 2009. SEMDSA Guidelines for Diagnosis and Management of Type 2 Diabetes Mellitus for Primary Health Care-2009. *Journal of Endocrinology, Metabolism and Diabetes of South Africa*, 14(1), pp.55–58.

Hayes, R.J. & Bennett, S., 1999. Simple sample size calculation for cluster-randomized trials. *International journal of epidemiology*, 28(2), pp.319–26.

Hemming, K., Girling, A.J., Sitch, A.J., Marsh, J. & Lilford, R.J., 2011. Sample size calculations for cluster randomised controlled trials with a fixed number of clusters. *BMC medical research methodology*, 11, p.102.

Hemminki, K., Li, X., Sundquist, K. & Sundquist, J., 2010. Familial risks for type 2 diabetes in Sweden. *Diabetes care*, 33(2), pp.293–7.

IDF, 2015. *Internationl Diabetes Association: Deabetes Atlas, 7th Edition. Brussels.*,

Inglehart, R. & Welzel, C., 2010. Changing Mass Priorities: The Link between Modernization and Democracy. *Perspectives on Politics*, 8(2), pp.551–567.

Jakubowski, E. & Busse, R., 1998. *Health care systems in the EU*,

Jonsson, I., Hallberg, L.-M. & Gustafsson, I., 2002. Cultural foodways in Sweden: repeated focus group interviews with Somalian women. *International Journal of Consumer Studies*, 26(4), pp.328–339.

Knowlton, A.R., 2003. Informal HIV caregiving in a vulnerable population: toward a network resource framework. *Social science & medicine (1982)*, 56(6), pp.1307–20.

Kunutsor, S., Walley, J., Katabira, E., Muchuro, S., Balidawa, H., Namagala, E., et al., 2011. Improving clinic attendance and adherence to antiretroviral therapy through a treatment supporter intervention in Uganda: a randomized controlled trial. *AIDS and behavior*, 15(8), pp.1795–802.

Lindström, J. & Tuomilehto, J., 2003. The diabetes risk score: a practical tool to predict type 2 diabetes risk. *Diabetes care*, 26(3), pp.725–31.

Lindström, M. & Sundquist, J., 2001. Immigration and leisure-time physical inactivity: a population-based study. *Ethnicity & health*, 6(2), pp.77–85.

Littenberg, B. & MacLean, C.D., 2006. Intra-cluster correlation coefficients in adults with diabetes in primary care practices: the Vermont Diabetes Information System field survey. *BMC medical research methodology*, 6, p.20.

Lozano, R., Naghavi, M., Foreman, K., Lim, S., Shibuya, K., Aboyans, V., et al., 2012. Global and regional mortality from 235 causes of death for 20 age groups in 1990 and 2010: a systematic analysis for the Global Burden of Disease Study 2010. *Lancet*, 380(9859), pp.2095–128.

Mayega, R.W., 2014. *Type 2 Diabetes in Rural Uganda: Prevalence, risk factors, perceptions and implications for the health system*. Karolinska Instututet and Makerere University.

Mayega, R.W., Etajak, S., Rutebemberwa, E., Tomson, G. & Kiguli, J., 2014. “Change means sacrificing a good life”: perceptions about severity of type 2 diabetes and preventive lifestyles among people afflicted or at high risk of type 2 diabetes in Iganga Uganda. *BMC public health*, 14, p.864.

Mayega, R.W., Guwatudde, D., Makumbi, F., Nakwagala, F.N., Peterson, S., Tomson, G., et al., 2013. Diabetes and pre-diabetes among persons aged 35 to 60 years in eastern Uganda: prevalence and associated factors. *PloS one*, 8(8), p.e72554.

Mayega, R.W., Makumbi, F., Rutebemberwa, E., Peterson, S., Östenson, C.-G., Tomson, G., et al., 2012. Modifiable socio-behavioural factors associated with overweight and hypertension among persons aged 35 to 60 years in eastern Uganda. *PloS one*, 7(10), p.e47632.

Mbanya, J.C.N., Motala, A.A., Sobngwi, E., Assah, F.K. & Enoru, S.T., 2010. Diabetes in sub-Saharan Africa. *Lancet (London, England)*, 375(9733), pp.2254–66.

Metzger, B.E., Gabbe, S.G., Persson, B., Buchanan, T.A., Catalano, P.A., Damm, P., et al., 2010. International association of diabetes and pregnancy study groups recommendations on the diagnosis and classification of hyperglycemia in pregnancy. *Diabetes care*, 33(3), pp.676–82.

Nachega, J.B., Chaisson, R.E., Goliath, R., Efron, A., Chaudhary, M.A., Ram, M., et al., 2010. Randomized controlled trial of trained patient-nominated treatment supporters providing partial directly observed antiretroviral therapy. *AIDS (London, England)*, 24(9), pp.1273–80.

Nalwadda, C.K., Waiswa, P., Kiguli, J., Namazzi, G., Namutamba, S., Tomson, G., et al., 2013. High compliance with newborn community-to-facility referral in eastern Uganda:.an opportunity to improve newborn survival. *PloS one*, 8(11), p.e81610.

van Olmen, J., Schellevis, F., Van Damme, W., Kegels, G. & Rasschaert, F., 2012. Management of Chronic Diseases in Sub-Saharan Africa: Cross-Fertilisation between HIV/AIDS and Diabetes Care. *Journal of tropical medicine*, 2012, p.349312.

Pan, X.R., Li, G.W., Hu, Y.H., Wang, J.X., Yang, W.Y., An, Z.X., et al., 1997. Effects of diet and exercise in preventing NIDDM in people with impaired glucose tolerance. The Da Qing IGT and Diabetes Study. *Diabetes care*, 20(4), pp.537–44.

Peer, N., Steyn, K., Lombard, C., Lambert, E. V, Vythilingum, B. & Levitt, N.S., 2012. Rising diabetes prevalence among urban-dwelling black South Africans. *PloS one*, 7(9), p.e43336.

Priebe, S. & Sandhu, S., 2008. *EUGATE Project: Best practice in health services for immigrants in Europe.*, University of London.

Rabkin, M., Melaku, Z., Bruce, K., Reja, A., Koler, A., Tadesse, Y., et al., 2012. Strengthening Health Systems for Chronic Care: Leveraging HIV Programs to Support Diabetes Services in Ethiopia and Swaziland. *Journal of tropical medicine*, 2012, pp.1–6.

Saha, S., Leijon, M., Gerdtham, U., Sundquist, K., Sundquist, J., Arvidsson, D., et al., 2013. A culturally adapted lifestyle intervention addressing a Middle Eastern immigrant population at risk of diabetes, the MEDIM (impact of Migration and Ethnicity on Diabetes In Malmö): study protocol for a randomized controlled trial. *Trials*, 14, p.279.

Samb, B., Desai, N., Nishtar, S., Mendis, S., Bekedam, H., Wright, A., et al., 2010. Prevention and management of chronic disease: a litmus test for health-systems strengthening in low-income and middle-income countries. *Lancet (London, England)*, 376(9754), pp.1785–97.

Schull, M.J., Cornick, R., Thompson, S., Faris, G., Fairall, L., Burciul, B., et al., 2011. From PALSA PLUS to PALM PLUS: adapting and developing a South African guideline and training intervention to better integrate HIV/AIDS care with primary care in rural health centers in Malawi. *Implementation science : IS*, 6, p.82.

Singer, J. & Willett, J., 2003. *Applied Longitudinal Data Analysis: Modeling Change and Event Occurence*, New York: Oxford University Press.

Singh, J., Liddy, C., Hogg, W. & Taljaard, M., 2015. Intracluster correlation coefficients for sample size calculations related to cardiovascular disease prevention and management in primary care practices. *BMC research notes*, 8, p.89.

Socialstyrelsen, S., Socialstyrelsen, Sweden. National guidelines for Methods of Preventing Disease – summary. Available at: http://www.socialstyrelsen.se/nationalguidelines [Accessed August 25, 2015].

Socialstyrelsen Sweden, National guidelines for Diabetes Care – summary. Available at: http://www.socialstyrelsen.se/nationalguidelines [Accessed August 25, 2015].

Steiner, K., 2013. *Diabetes among Turkish Immigrants in Sweden. A study of prevalence and risk factors (Doctoral degree - PhD). Stockholm: Karolinska Intitutet*.

Stratton, I.M., Adler, A.I., Neil, H.A.W., Matthews, D.R., Manley, S.E., Cull, C.A., et al., 2000. Association of glycaemia with macrovascular and prospective observational study. *BMJ*, 321, pp.405–412.

Syed, S.B., Dadwal, V. & Martin, G., 2013. Reverse innovation in global health systems: towards global innovation flow. *Globalization and health*, 9, p.36.

Tumusiime, P., Gonani, A., Walker, O., Asbu, E.Z., Awases, M. & Kariyo, P.C., 2012. *Health systems in sub-Saharan Africa: What is their status and role in meeting the health Millennium Development Goals?*,

Tuomilehto, J., Lindström, J., Eriksson, J.G., Valle, T.T., Hämäläinen, H., Ilanne-Parikka, P., et al., 2001. Prevention of type 2 diabetes mellitus by changes in lifestyle among subjects with impaired glucose tolerance. *The New England journal of medicine*, 344(18), pp.1343–50.

Wändell, P.E. & Gåfvels, C., 2007. High prevalence of diabetes among immigrants from non-European countries in Sweden. *Primary care diabetes*, 1(1), pp.13–6.

WHO, 2006. Definition and diagnosis of diabetes mellitus and intermediate hyperglycemia. *A report of a WHO/IDF consultation*. Available at: http://www.http//who.int/iris/bitstream/10665/43588/1/9241594934_eng.pdf [Accessed May 24, 2016].

WHO, 2009. *Global health risks: mortality and burden of disease attributable to selected major risks*, Geneva.

WHO, 2013. Towards-people-centred-health-systems-an-innovative-approach-for-better-health-outcomes. Copenhagen: Division of Health Systems and Public Health, World Health Ornganization, Regional office for Europe. Available at: http://www.euro.who.int/__data/assets/pdf_file/0006/186756/Towards-people-centred-health-systems-an-innovative-approach-for-better-health-outcomes.pdf.

WHO, 2011. WHO | Use of glycated haemoglobin (HbA1c) in the diagnosis of diabetes mellitus. *Abbreviated report of a WHO consultation*, pp.1–25.

Woolf, S., Jonas, S. & E., K.-L., 2007. *Health Promotion asnd Disease Prevention in Clinical Practice*, Lippincott Williams & Wilkins.

Zeger, S.L. & Liang, K.Y., 1986. Longitudinal data analysis for discrete and continuous outcomes. *Biometrics*, 42(1), pp.121–30.

# 5.0 APPENDICES

## 5,1 Planned Timeline

|  | **2016** | | | | | | | | | | | | **2017** | | | | | | | | | | | | **2018** | | | | | | | | | | | | **2019** | | | | | |
| --- | --- | --- | --- | --- | --- | --- | --- | --- | --- | --- | --- | --- | --- | --- | --- | --- | --- | --- | --- | --- | --- | --- | --- | --- | --- | --- | --- | --- | --- | --- | --- | --- | --- | --- | --- | --- | --- | --- | --- | --- | --- | --- |
|  | J | F | M | A | M | J | J | A | S | O | N | D | J | F | M | A | M | J | J | A | S | O | N | D | J | F | M | A | M | J | J | A | S | O | N | D | J | F | M | A | M | J |
| Trial proposal development | X | X | X | X |  |  |  |  |  |  |  |  |  |  |  |  |  |  |  |  |  |  |  |  |  |  |  |  |  |  |  |  |  |  |  |  |  |  |  |  |  |  |
| Data collection tools and guidelines development |  | X | X | X | X | X |  |  |  |  |  |  |  |  |  |  |  |  |  |  |  |  |  |  |  |  |  |  |  |  |  |  |  |  |  |  |  |  |  |  |  |  |
| Site-specific additions/modifications to the proposal |  |  |  |  | X | X |  |  |  |  |  |  |  |  |  |  |  |  |  |  |  |  |  |  |  |  |  |  |  |  |  |  |  |  |  |  |  |  |  |  |  |  |
| Acquire equipment, print tools, etc |  |  |  |  |  | X | X | X | X |  |  |  |  |  |  |  |  |  |  |  |  |  |  |  |  |  |  |  |  |  |  |  |  |  |  |  |  |  |  |  |  |  |
| Ethical approval processes |  |  |  |  |  |  | X | X | X |  |  |  |  |  |  |  |  |  |  |  |  |  |  |  |  |  |  |  |  |  |  |  |  |  |  |  |  |  |  |  |  |  |
| Community sensitization |  |  |  |  |  |  | X | X | X |  |  |  |  |  |  |  |  |  |  |  |  |  |  |  |  |  |  |  |  |  |  |  |  |  |  |  |  |  |  |  |  |  |
| Hire field personnel |  |  |  |  |  | X | X | X |  |  |  |  |  |  |  |  |  |  |  |  |  |  |  |  |  |  |  |  |  |  |  |  |  |  |  |  |  |  |  |  |  |  |
| Pre-test tools and guidelines |  |  |  |  |  |  | X | X |  |  |  |  |  |  |  |  |  |  |  |  |  |  |  |  |  |  |  |  |  |  |  |  |  |  |  |  |  |  |  |  |  |  |
| Database structure dev't |  |  |  |  |  |  | X | X | X |  |  |  |  |  |  |  |  |  |  |  |  |  |  |  |  |  |  |  |  |  |  |  |  |  |  |  |  |  |  |  |  |  |
| Training of study staff |  |  |  |  |  |  |  | X | X |  |  |  |  |  |  |  |  |  |  |  |  |  |  |  |  |  |  |  |  |  |  |  |  |  |  |  |  |  |  |  |  |  |
| Pilot implementation of intervention |  |  |  |  |  |  |  |  |  | X |  |  |  |  |  |  |  |  |  |  |  |  |  |  |  |  |  |  |  |  |  |  |  |  |  |  |  |  |  |  |  |  |
| Do necessary adjustment to the intervention |  |  |  |  |  |  |  |  |  |  | X | X |  |  |  |  |  |  |  |  |  |  |  |  |  |  |  |  |  |  |  |  |  |  |  |  |  |  |  |  |  |  |
| Enrollment into main trial |  |  |  |  |  |  |  |  |  |  |  |  | X | X | X | X | X | X | X | X | X | X | X |  |  |  |  |  |  |  |  |  |  |  |  |  |  |  |  |  |  |  |
| Follow-up |  |  |  |  |  |  |  |  |  |  |  |  | X | X | X | X | X | X | X | X | X | X | X | X | X | X | X | X | X | X | X | X | X | X | X | X |  |  |  |  |  |  |
| Data entry |  |  |  |  |  |  |  |  |  |  |  |  | X | X | X | X | X | X | X | X | X | X | X | X | X | X | X | X | X | X | X | X | X | X | X | X | X |  |  |  |  |  |
| Data cleaning |  |  |  |  |  |  |  |  |  |  |  |  |  | X | X | X | X | X | X | X | X | X | X | X | X | X | X | X | X | X | X | X | X | X | X | X | X |  |  |  |  |  |
| Preliminary report writing |  |  |  |  |  |  |  |  |  |  |  |  |  |  |  |  |  |  |  |  |  |  |  |  |  |  |  |  |  |  | X | X | X | X | X | X |  |  |  |  |  |  |
| Merging data from sites |  |  |  |  |  |  |  |  |  |  |  |  |  |  |  |  |  |  |  |  |  |  |  |  |  |  |  |  |  |  |  | X |  |  |  |  |  |  |  |  |  |  |
| Data analysis |  |  |  |  |  |  |  |  |  |  |  |  |  |  |  |  |  |  |  |  |  |  |  |  |  |  |  |  |  |  |  |  |  |  |  |  | X | X | X |  |  |  |
| Final report writing |  |  |  |  |  |  |  |  |  |  |  |  |  |  |  |  |  |  |  |  |  |  |  |  |  |  |  |  |  |  |  |  |  |  |  |  |  | X | X | X |  |  |
|  |  |  |  |  |  |  |  |  |  |  |  |  |  |  |  |  |  |  |  |  |  |  |  |  |  |  |  |  |  |  |  |  |  |  |  |  |  |  |  |  |  |  |

5.2: Care companion guidelines

1. **RECRUITMENT GUIDELINES**

**Recruitment of care companions screening (*to be answered by the patient*)(**[**Knowlton 2003**](#_ENREF_1)**)**

1. Who would you prefer to help you out if you needed help? E.g if you couldn’t take care of yourself, who would you want to stay with?
2. Who do you think will be the most helpful person in your life in dealing with Diabetes/prediabetes?
3. Is there anyone else who would be especially helpful with dealing with diabetes/prediabetes?

**Criteria for selection (*information for clinicians*)(**[**Nachega, Chaisson et al. 2010**](#_ENREF_3)**,** [**Kunutsor, Walley et al. 2011**](#_ENREF_2)**)**

1. Must be living in the patients’ household/neighborhood
2. Aware of patients’ health condition
3. Has accepted patients’ health condition and is a confidant
4. Willing and committed to support patient with care for a long period of time
5. **ROLE OF CARE COMPANIIONS**
6. Remind patients to go for next clinic appointments and peer group sessions.
7. Remind patients to take diabetes medication; observe and record while patient takes at least one dose per day?([Nachega, Chaisson et al. 2010](#_ENREF_3)).
8. Observe and confirm that daily/weekly physical activity goals are met.
9. Ensure a healthy diet for the patient.
10. **TRAINING GUIDELINES**

Patients and their selected care companions will undergo 90 minutes of baseline training on;

1. Diabetes/ prediabetes (causes, diagnosis, signs and symptoms, treatment and control)
2. Diabetes/prediabetes lifestyle (physical activity, diet)
3. Role of care companions

Care companions will receive booster health education every 3 months

5.3 Data Collection Tools


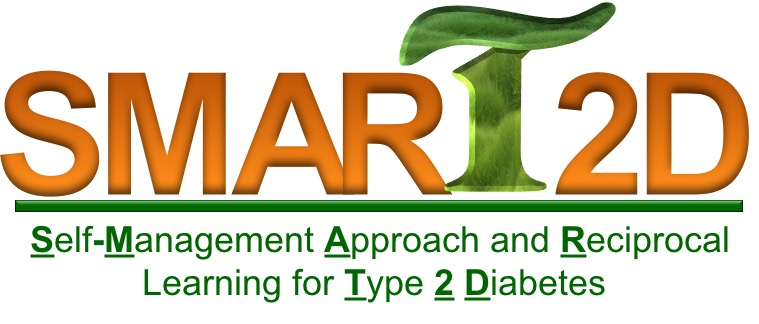


| **WP 5** |
| --- |
| **DATA COLLECTION TOOLS**  **(BASELINE & INTERMEDIATE TIME-POINTS ONLY)** |
| Generic Version for **all participants in SMART2D trial** |

| **Lead Institution** | Uppsala University |
| --- | --- |
| **Coordinating Investigator** | Meena Daivadanam & Josefien van Olmen |
| **Other collaborating Investigators** | ITM: Jeroen de Man  KI/UU: Helle Mölsted Alvesson, Aravinda Berggreen Clausen, Linda Timm, Juliet Aweko, Helena Elmståhl  MakSPH: David Guwatudde, Elizabeth Ekirapa, Roy Mayega, Francis Kasujja, Gloria Naggayi  UWC: Peter Delobelle, Mark Spires, Bonaventure Egbujie, Lungiswa Tsolekile, Hanani Tabana, Mariam Hassan  CCSF: Pilvikki Absetz |
| **Special acknowledgements** | Irma Nordin, Agneta Andersson & Dell Saulnier |
| **Version** | **5.0, dated 30/01/2017**  Changes from 4.0 based upon:   - Formatting the tools for use in Redcap and on paper (DS). - Double-checking tools for errors and uniform coding (DS and DG)   **Status: FINAL VERSION for baseline and intermediate time points.**  *Please note that all questions relevant for end-line have been removed from this version and will be developed further and added to end-line tools.* |
| **Changes from previous version (from version 4.0)**  All changes highlighted in yellow in the document | **ALL PARTS**   1. All variables that have “yes” or “no” answers have been coded as:   Yes = 1  No = 0   1. All variables that use “Don’t know/don’t remember”, “Not applicable” or “Refused to answer” have been coded as:   Don’t know or don’t remember = 777  Not applicable = 888  Refused to answer = 999  **PART I**   1. New variable (study_id) added for screening ID before Step 1, ***only for sites using Redcap for data collection*** 2. Instruction variable (Inst_eli_1) added after Step 1 if eli_1=no 3. REDCAP USERS: STEP 2 d: third option (“participant has pre-diabetes”) added, and instruction text for data collectors (“For interviewers: If the participant has pre-diabetes, mark the selection ‘Participant has pre-diabetes’”) 4. REDCAP USERS: STEP 2 e: third option (“participant is male”) added, and instruction text for data collectors (“For interviewers: If the participant is male, mark the selection ‘Participant is male’”) 5. Instruction variable (inst_eli_2) added after Step 2 h if all Step 2 1-h are marked no 6. Section 3 d: dem_1d_other changed to “If 9, specify:” 7. Variable (inst_dem_1f_opt) added to choose option for reporting household income. Option 1=salary per day * number of days of work per month, Option 2=salary per month 8. REDCAP USERS: Note that variables dem_1f1 and dem_1f2 must be calculated by hand 9. Section 5: Instruction variable (inst_diet1) added at start 10. Section 5: The wording “How many servings of ____ do you eat on a typical day?” has been added to each food option (e.g. whole fruit, cut fruit, etc.). Applies to the following variables:     1. Diet_1b1-4     2. Diet_1d1-4     3. Diet_1h1-2     4. Diet_1j1-2 11. Instruction variable (inst_diet3) added after Section 5 p. 12. Section 5 t: 13. Start of diet_1t1a (“How many servings of sweetened, non-carbonated drinks…”) has been split from the question into a new instruction variable (inst_diet2a) 14. Diet_1t1a-f have been changed to “Number of Showcard 5.__ drinks consumed” 15. New variables added (diet_1t1a_total to diet_1t1f_total) to calculate total in mL: “Amount in mL of Showcard 5.___ drinks consumed:” (**Note. In Redcap, the calculation will be automatic) 16. Start of diet_1t2a (“How many servings of sugar-sweeteened fizzy drinks…”) has been split from the question into a new instruction variable (inst_diet2b) 17. Diet_1t2a-f have been changed to “Number of Showcard 5.__ drinks consumed” 18. New variables added (diet_1t2a_total to diet_1t2f_total) to calculate total in mL: “Amount in mL of Showcard 5.___ drinks consumed:” (**Note. In Redcap, the calculation will be automatic) 19. Section 10: Instruction variable (inst_fpg1) added if fpg_1a=yes 20. Section 10: Variable (inst_fpg1b_opt) added to choose option for reporting FPG. Option 1=mmol/L, Option 2=mg/dL 21. Section 10 : Variable (hba1c_1a_opt) added to choose option for reporting HBA1C. Option 1=percent (%), Option 2=mmol/m   **PART II**   1. Instruction variable (inst_sos1) added at start of Section 13 on how to use Likert scale 2. Text on peer groups and peer leaders removed from start of Section 13 as no longer asking about peer groups/leaders 3. Instruction variable (inst_eff1) added at start of Section 14 on how to use Likert scale 4. Section 14 intro: text “and do physical activity” removed here 5. Section 14 after f: text “maintain a healthy diet and” removed here 6. Instruction variable (inst_aus1) added at start of Section 15 on how to use Likert scale 7. Instruction variable (inst_sreg1) added at start of Section 16 on how to use Likert scale 8. Instruction variable (inst_psa1) added at start of Section 17 on how to use Likert scale 9. Instruction variable (inst_ts1) added at start of Section 18 on how to use Likert scale 10. Section 19: Relabelled and recoded questions L through P to G-K   **PART III:**   1. Instruction variable (inst_oopi1) added at start of Section 21B for referring to the last 3 hospitalizations 2. Section 21B, J-W: New codes for each hospitalization option (e.g. oopi_1k1-3, oopi_1m1-3) 3. Instruction variable (inst_oopi2) added at after question L for reporting hospitalization costs   **SHOWCARDS**   1. Showcard 7 option for Not Applicable changed to 888 |
|  | - Make appropriate changes to translated tools. - This is final version to be administered once questions are clarified. |

INSTRUCTIONS to site teams

- This set of data collection tools contains all the generic instruments that should be used during screening/recruitment and baseline.
- All sections are numbered. Kindly do not change the section numbers, question numbers or codes in this version.
- Please provide laminated SHOWCARDS in colour to all data collectors. Instruct data collectors to give the appropriate SHOWCARD for each section to the participant to make it easier for them to respond.
- It is recommended that baseline data collection is staggered over two occasions to decrease participant fatigue and improve the quality of the data collected. Please follow the recommended steps below to divide the baseline data collection in two steps.
  - **During RECRUITMENT visit**

STEP 1 = Section 1 to confirm eligibility 🡺 Sign informed consent 🡺 Section 2 to assign identifiers 🡺 Sections 2-11.2 🡺 Fill in start date for pedometer reading in Section 11.3 🡺 Give instructions on pedometer use + demonstrate use of pedometer + reset and help the participant to wear a masked pedometer 🡺 Confirm instructions and decide on date to collect back the pedometer after 7 days 🡺 If you are unable to collect back the pedometer after 7 days – give specific instructions on what the participant is expected to do 🡺 BREAK off baseline data collection HERE.

- - **During visit to COLLECT PEDOMETER**

STEP 2 = Meet on previously decided date 🡺 Check pedometer and enter reading and end-date in section 11.3 🡺 Complete sections 12-21. END of BASELINE data collection.

About this document

This is a master document containing the complete set of data collection tools that will be administered using a survey methodology. Please note, that evaluations that involve qualitative methodologies are not included here.

The document is divided into four parts:

*Part I: Basic participant data*

*Part II: Intervention process measures (based on Self Determination Theory)*

*Part III: Selected context measures and expenditure*

*Part IV: Process and fidelity checks*

Please note that this document includes tools pertaining to individuals, facilities and local community. It is not meant to be administered together in one visit. Part I includes the set of tools that should be administered together in one baseline/end-line visit.

Sites have to decide on how to administer parts II and III based on the feasibility of administration. Please take care to include relevant identification information in all sets of questionnaires (particularly, if they are collected during separate visits). Some of the tools can be measured in only a sub-set of the study sample and these have been marked with an * in the tables at the beginning of each part.

**Structure of this document**

Each part starts with a table outlining the individual tools included in that section, the sources of the same and the data collection time-points for each. A framework which explains the choice of tools is also included for parts II and III.

**Variable coding strategy and tool formats:** As far as possible, all tools have been converted to a 4-column format – s.no, questions, response and variable code. Variable coding format and characters have been chosen such that they will be accepted in different statistical software packages such as STATA, SPSS, etc. Please ensure that the codes are same for each site – to avoid any problems while merging data. If sites include additional questions to the ones in the generic format, please use different codes to the ones in the generic format.

Each response is coded as a separate variable. All variables (except ID and measurement codes) are coded with the format AAAA_00x.

*For tools that will be administered multiple times (e.g. baseline and end-line):*

- AAAA represents 2-4 alphabets representing the section. E.g. Demographic – DEM;
- 00 represents the month of data collection. E.g. Baseline will be 1, end-line will be 12; and
- x is consecutive alphabets representing question numbers in each section.
- Any sub-questions under each question will be further identified by and underscore + number + alphabet as needed (e.g. _1 or _1a)
- Sometimes additional details are needed and these are added after an underscore.
- E.g. DIET_1a is first question in section on dietary behaviour collected during baseline. When the same variable is collected at end-line, it will be DIET_12a.

*For tools that will be administered only once (e.g. either baseline or end-line):*

- AAAA represents 2-4 alphabets representing the section. E.g. Demographic – DEM;
- 00 represents the question numbers
- x represents any sub-questions under each
- E.g. ELI_2a is first sub-question under question number 2 in the section on eligibility and recruitment check. Sometimes additional details are needed and these are added after an underscore.

PART I: Basic Participant Data

**Table 1: Details of tools, sources and data collection points for Part I**

| Questionnaires | Source | BL | M2-11 | EL |
| --- | --- | --- | --- | --- |
| **Eligibility & Recruitment Check** | - SMART2D | X |  |  |
| **Identification Information** | - SMART2D | X |  |  |
| **Demographic & Socioeconomic Measures** | - WHO STEPs Survey | X |  |  |
| **Medical and Medication History** | - WHO STEPs Survey  - GACD diabetes data dictionary | X |  |  |
| **Behavioural Measures – Diet** | - WHO STEPs Survey  - Summary of Diabetes Self-Care Activities (SDSCA) | X |  | X |
| **Behavioural Measures – Physical Activity** | - Summary of Diabetes Self-Care Activities (SDSCA)  - Single Question Physical Activity Questionnaire (Rose et al) <http://appliedresearch.cancer.gov/paq/q098.html> | X |  | X |
| **Behavioural Measures – Tobacco Use** | - WHO STEPs Survey | X |  | X |
| **Behavioural Measures – Alcohol Consumption** | - WHO STEPs Survey | X |  | X |
| **Other Behavioural Measures – Foot care** | - Summary of Diabetes Self-Care Activities (SDSCA) | X |  | X |
| **Biochemical – FPG^#^** | - WHO STEPs Survey | X | X |  |
| **Biochemical – HbA1c** | - WHO STEPs Survey | X |  | X |
| **Anthropometry and others: Height, weight & Waist circumference** | - WHO STEPs Survey | X |  | X |
| **Anthropometry and others: Blood Pressure** | - WHO STEPs Survey | X |  | X |
| **Anthropometry and others: 7-day pedometer steps** | - SMART2D | X |  | X |
| **Community activities §** | - Kerala Diabetes Prevention Programme |  |  | X |

^#^ FPG measurements will be used for screening in Ug & SA and interim monitoring in Ug

§ Not included in this document – to be finalised before end-line

| **SECTION 1: ELIGIBILITY & RECRUITMENT CHECK**  (Instructions: To be filled in by recruiting personnel before participants sign informed consent) | | | | |
| --- | --- | --- | --- | --- |
| **STEPs** | **Description & questions** | **RESPONSE** | | **CODE** |
|  | REDCAP: Assign the screening ID number | └──┘└──┘└──┘ | | study_id |
| **STEP 1** | **RECRUITMENT algorithm** | **No** | **Yes** |  |
|  | Does the site-specific recruitment algorithm for the screening and diagnostic tests (HbA1c or FPG) for the individual show RECRUIT? | └──┘ | └──┘ | ELI_1 |
|  | "If NO, the subject is NOT ELIGIBLE to be recruited for the trial. Do not continue with recruitment." |  |  | Inst_eli_1 |
| 🡺 If YES, proceed to STEP 2 | | | |  |
| **STEP 2** | **Answer the following ELIGIBILITY questions** | **No** | **Yes** |  |
| a) | Has the individual been a resident in the location of the interview for less than 6 months? | └──┘ | └──┘ | ELI_2a |
| b) | Is the individual less than 30 years, or older than 75 years of age | └──┘ | └──┘ | ELI_2b |
| c) | Has the individual indicated that he/she has plans of migrating out of the study area over the next 12 months? | └──┘ | └──┘ | ELI_2c |
| **For participants with pre-diabetes, SKIP to (e)** | | | | |
| d) | Was the individual diagnosed with diabetes longer than 12 months before being screened for the SMART2D intervention trial?  REDCAP: "FOR INTERVIEWERS: If the participant has pre-diabetes, mark the selection 'Participant has pre-diabetes'" | 0 = No  1 = Yes  2 = Participant has pre-diabetes | | ELI_2d |
| e) | If female, is the individual pregnant?  **If male, SKIP to (f)**  REDCAP: "FOR INTERVIEWERS: If the participant is male, mark the selection Participant is male" | 0 = No  1 = Yes  2 = Participant is male | | ELI_2e |
| f) | Has the individual been diagnosed with heart disease, stroke or peripheral vascular disease/serious mental disorders affecting cognitive functions? | └──┘ | └──┘ | ELI_2f |
| g) | Has any other individual in the same household been enrolled in this study? | └──┘ | └──┘ | ELI_2g |
| h) | Is the individual enrolled in any other study related to diabetes/ self-management | └──┘ | └──┘ | ELI_2h |
| 🡺 If all of the responses in STEP 2 are NO, the subject is ELIGIBLE to be recruited for the trial. If more than one individual in a household is found eligible, apply the KISH methodology (See Appendix 1). | | | | inst_eli_2 |

| **SECTION 2: IDENTIFICATION INFORMATION**  (Instruction: To be included in each set of participant questionnaires) | | | |
| --- | --- | --- | --- |
| **S.NO:** | **Questions** | **Response** | **Code** |
| A | Site Identification Number  UG/SA/SW | └──┘└──┘ | SI_ID |
| B | Cluster/facility Identification number  *Each site to assign numbers to each cluster (SA: 1-2, SW: 1-4, UG: 1-9)* | └──┘ | CL_ID |
| C | Trial arm identification code | 1= Facility only  2 = Facility + community └──┘  3 = No intervention (Uganda only) | TA_ID |
| D | Unique Participant ID number *(Assigned by study team in the field - assign a range of numbers to each cluster)* | └──┘└──┘└──┘ | PA_ID |
| E | To which diagnostic group is the participant recruited? | 1 = Diabetes  2 = Pre-diabetes └──┘ | DX_ID |
| F | Month of visit  0 3 6 9 12 | └─┴─┘ | MTH_ID |

| **SECTION 3: DEMOGRAPHIC & SOCIOECONOMIC MEASURES** | | | |
| --- | --- | --- | --- |
| **S.NO:** | **Questions** | **Response** | **Code** |
| A | What is your year of birth?  Don’t know: 777  Refused to answer: 999  *If participants do not know the year of birth, prompt for nearest estimate* | YYYY └─┴─┴─┴─┘ | DEM_1a |
| B | Sex of participant  Male = 1  Female = 2 | └──┘ | DEM_1b |
| C | What is your highest level of education passed?  1.None  2. School grades <5  3. School grades 5-7  4. School grades 8-10  5. School grades 11-12  6. Vocational training, with school grade > 12 but no university education  7. University-level undergraduate education  7. University-level graduate / post-graduate education  Don’t know 777  Refused to answer 999 | └─┴─┘ | DEM_1c |
| D | What is your current employment?   1. Employed in public sector 2. Employed in private sector 3. Self-employed 4. Daily wage employment 5. Un-employed 6. Retired 7. Petty trader 8. Peasant farmer 9. Other   777. Don’t know  999. Refused to answer | └──┘ | DEM_1d |
|  |  | If 9, specify: _______________ | DEM_1d_other |
| E | In total, how many members reside in your household (share a common kitchen/ share meals/ spend at least/on average 3 nights in a week at your home, including children of whom you have joint custody that live with you full or part time)? | └─┴─┘ | DEM_1e |
|  | "Choose ONE option to report average household income:  "Option 1 - salary per day * number of days of work per month  Option 2 - Salary per month" | 1 = Option 1  2 = Option 2 | inst_dem_1f_opt |
| F | What is the average income of your household?  (Record only ONE option)  *Instruction: Please answer in Ugandan shilling for Uganda, South African Rand in South Africa and Swedish Kroner for Sweden.*  Don’t know code 777  Refused to answer code 999 | OPTION 1:  └─┴─┴─┴─┴─┘/ day * ______no. of days of work per month  OPTION 2:  └─┴─┴─┴─┴─┴─┘/ Month | DEM_1f1  DEM_1f2 |
| G | What is your current marital status?  0 = Never married or cohabited  1 = **Presently** with somebody: Married/cohabiting  2 = **Presently** not with somebody: Separated/Divorced  3 = **Presently** not with somebody: widowed  4=Other | └──┘ | DEM_1g |
|  |  | **If ‘other’, please specify**  **_______________________** | DEM_1g_other |
| H | What is your country of birth?  777 = Don’t know  999 = Refused | **_____________________** | DEM_1h |

| **SECTION 4: MEDICAL AND MEDICATION HISTORY** | | | | |
| --- | --- | --- | --- | --- |
| **S.NO:** | **Questions** | | **Response** | **Code** |
| A | Have you ever been told by a doctor or other health worker that you have raised blood pressure or hypertension?  No = 0  Yes = 1  Don’t know/unsure = 777 | └──┘ | | MED_1a |
| B | Have you ever taken any drugs (medication) for raised blood pressure prescribed by a doctor or other health worker?  No = 0  Yes = 1  Don’t know/unsure = 777  *Ask the participant to only consider drugs for raised blood pressure prescribed by a doctor or other health worker (if possible interviewer to observe medication for confirmation)* | └──┘ | | MED_1b |
| C | Have you ever been told by a doctor or other health worker that you have raised blood sugar or diabetes?  No = 0  Yes = 1  Don’t know/unsure = 777 | └──┘ | | MED_1c |
| D | Have you ever been told by a doctor or other health worker that you have kidney disease?  No = 0  Yes = 1  Don’t know/unsure = 777 | └──┘ | | MED_1d |
| E | Have you developed any foot ulcers in the past year?  No = 0  Yes = 1  Don’t know/unsure = 777 | └──┘ | | MED_1e |
| F | Have you had any loss of vision in the past year?  No = 0  Yes = 1  Don’t know/unsure = 777 | └──┘ | | MED_1f |
| **For participants with pre-diabetes, SKIP to J** | | | | |
| G | Are you currently on any injectable insulin?  No = 0  Yes = 1  Don’t know/unsure = 777  If, NO, skip to I | └──┘ | | MED_1g |
| H | Are you currently on any injectables other than insulin?  No = 0  Yes = 1  Don’t know/unsure = 777 | └──┘ | | MED_1h |
| I | Are you currently on any oral hypoglycemic agents  No = 0  Yes = 1  Don’t know/unsure = 777 | └──┘ | | MED_1i |
| J | Are you currently on any traditional or complementary medicines to treat or prevent diabetes or pre-diabetes?  No = 0  Yes = 1  Don’t know/unsure = 777 | └──┘ | | MED_1j |

| **SECTION 5: BEHAVIOURAL MEASURES – DIET** | | | | | |
| --- | --- | --- | --- | --- | --- |
| The next questions ask about the fruits and vegetables that you usually eat. I have a nutrition card here that shows you some portion sizes of the foods we are talking about. Each picture represents the size of a serving. As you answer these questions please think of a TYPICAL WEEK and a TYPICAL DAY in the LAST MONTH.  *(Use the SHOW CARDS provided - Important for interviewers to show the right card for the right question as indicated: so for whole fruit – show card 1, for cut fruits show card 2, etc. Each show card represents 1 serving of the food indicated.)* | | | | | inst_diet1 |
| **S.NO:** | **Question** | | **Response** | **Code** | |
| A | In a typical week, on how many days do you eat **fruit**?  Don’t know 777 | | Number of days└─┴─┘ *If zero, go to C* | DIET_1a | |
| B | 1. How many servings of **whole fruit** do you eat on a typical day? (SHOWCARD 1)  Don’t know 777  2. How many servings of **cut or small fruit (fresh or frozen)** do you eat on a typical day? (SHOWCARD 2)  Don’t know 777  3. How many servings of **canned fruit** do you eat on a typical day? (SHOWCARD 2)  Don’t know 777  4. How many servings of **dried fruits** do you eat on a typical day? (SHOWCARD 4) | | Number of servings:  1. └─┴─┘.└─┴─┘  2. └─┴─┘.└─┴─┘  3. └─┴─┘.└─┴─┘  4. └─┴─┘.└─┴─┘ | DIET_1b1,2,3,4 | |
| C | In a typical week, on how many days do you eat **vegetables** like tomatoes, carrots, onions, etc. (excluding tubers and high starch vegetables such as cassava, potatoes, matoke, yams, sweet potatoes)?  Don’t know 777 | | Number of days └─┴─┘ *If zero, go to E* | DIET_1c | |
| D | 1. How many servings of **cut vegetables (fresh or frozen)** do you eat on a typical day?  (SHOWCARD 2)  Don’t know 777  2. How many servings of **canned vegetables** do you eat on a typical day? (SHOWCARD 2)  Don’t know 777  3. How many servings of **uncooked leafy vegetables** do you eat on a typical day?  (SHOWCARD 1) | | Number of servings:  1. └─┴─┘.└─┴─┘  2. └─┴─┘.└─┴─┘  3. └─┴─┘.└─┴─┘ | DIET_1d1,2,3 | |
| E | In a typical week, how many days do you eat **tubers and high starch vegetables (**such as cassava, potatoes, matoke/plantain, yams, sweet potatoes)?  Don’t know 777 | | Number of days└─┴─┘ *If zero, go to G* | DIET_1e | |
| F | How many servings of **tubers and high starch vegetables** do you eat on one of those days? (Such as cassava, potatoes, matoke/plantain, yams, sweet potatoes) (SHOWCARD 1)  Don’t know 777 | | Number of servings└─┴─┘.└─┴─┘ | DIET_1f | |
| G | In a typical week, how many days do you eat **refined starch products**, (such as white rice, pasta, white breads, maize meal, cassava flour meal, pap)?  Don’t know 777 | | Number of days└─┴─┘ *If zero, go to K* | DIET_1g | |
| H | 1. "How many servings of bread do you eat on a typical day?" (SHOWCARD 3)  Don’t know 777  2. "How many servings of other refined starch products (such as white rice, pasta, maize meal pap) do you eat on a typical day?” (SHOWCARD 1)  Don’t know 777 | | Number of servings:   1. └─┴─┘.└─┴─┘ 2. └─┴─┘.└─┴─┘ | DIET_1h1,2 | |
| I | In a typical week, how many days do you eat **non-refined starch** (such as brown rice, whole grain pasta, wholegrain cereal, samp or whole meal/whole wheat / brown breads)?  Don’t know 777 | | Number of days└─┴─┘ *If zero, go to M* | DIET_1i | |
| J | 1. "How many servings of non-refined bread do you eat on a typical day?" (SHOWCARD 3)  Don’t know 777  2. "How many servings of other non-refined starch products (such as brown rice, whole grain pasta, wholegrain cereal, samp or whole meal) do you eat on a typical day?" (SHOWCARD 1)  Don’t know 777 | | Number of servings:   1. └─┴─┘.└─┴─┘ 2. └─┴─┘.└─┴─┘ | DIET_1j1,2 | |
| K | In a typical week, how many days do you eat **fish**?  Don’t know 777 | | Number of days└─┴─┘ *If zero, go to O* | DIET_1k | |
| L | How many servings of **fish**, do you eat on one of those days?  (SHOWCARD 3)  Don’t know 777 | | Number of servings└─┴─┘.└─┴─┘ | DIET_1l | |
| The next questions ask about the way you most often prepare different types of food in your household. | | | | | |
| M | Which cooking method do you usually use when preparing chicken?  1 = Grill/Oven/Roast  2 = Fry  3 = Boil  4 = Steam  5 = Other (specify) | | └──┘  If 5, please specify  **_____________________** | DIET_1m  DIET_1m_other | |
| N | Which cooking method do you usually use when preparing other meat?  1 = Grill/Oven/Roast  2 = Fry  3 = Boil  4 = Steam  5 = Other (specify) | | └──┘  If 5, please specify  **___________________** | DIET_1n  DIET_1n_other | |
| O | Which cooking method do you usually use when preparing fish?  1 = Grill/Oven/Roast  2 = Fry  3 = Boil  4 = Steam  5 = Other (specify) | | └──┘  If 5, please specify  **___________________** | DIET_1o  DIET_1o_other | |
| P | Which cooking method do you usually use when preparing vegetables?  1 = Grill/Oven/Roast  2 = Fry  3 = Boil  4 = Steam  5 = Other (specify) | | └──┘  If 5, please specify  **___________________** | DIET_1p  DIET_1p_other | |
| The next questions ask about the oil or fat that is most often used for meal preparation in your household, and about meals that you eat outside a home. | | | **How often** do you use the following **oils and fats** in your household? | inst_diet3 | |
| Q | | Use the scale below:  1 = Daily  2 = 5-6 days per week  3 = 2-4 days per week  4 = 1-2 days per week  5 = 1-3 days per month  6 = Occasionally, usually less than once a month  7 = Never  Don’t know 777 | 1. Vegetable oil └──┘ 2. Lard or suet └──┘ 3. Butter or ghee └──┘ 4. Margarine └──┘ 5. Mixed butter + oil combination └──┘ 6. Other └──┘If Other, specify below   Other, please specify  ____________________________ | DIET_1q_1-6 | |
|  |  |  |  | DIET_1q_other | |
| R | | On average, how often do you eat meals that were **not prepared at a home**? (By meal, we mean breakfast, lunch or dinner)  1 = Daily = 1  2 = 5-6 days per week  3 = 2-4 days per week  4 = 1-2 days per week  5 = 1-3 days per month  6 = Occasionally, usually less than once a month  7 = Never  Don’t know 777 | └──┘ | DIET_1r | |
| S | | In a typical week, on how many days do you drink **sugar-sweetened beverages** (such as sodas, and other non-carbonated commercially prepared fruit drinks)?  Don’t know 777 | Number of days└─┴─┘ *If zero, go to SECTION 6* | DIET_1s | |
| T | | How many servings of **sweetened non-carbonated drinks** (ready to drink diluted cordials and syrups/commercially prepared sweetened fruit drinks) do you drink on a typical day? | | inst_diet2a | |
|  |  | (SHOWCARD 5)  *Enter the number of drinks consumed per category as shown in the show card.*  Don’t know 777 | 1.a Number of Showcard 5.1 drinks consumed: └──┘.└─┴─┘  1.a.total: Amount in mL of Showcard 5.1 drinks consumed: ____”  1.b Number of Showcard 5.2 drinks consumed: └──┘.└─┴─┘  1.b.total: Amount in mL of Showcard 5.2 drinks consumed: ____”  1.c Number of Showcard 5.3 drinks consumed: └──┘.└─┴─┘  1.c.total: Amount in mL of Showcard 5.3 drinks consumed: ____”  1.d Number of Showcard 5.4 drinks consumed: └──┘.└─┴─┘  1.d.total: Amount in mL of Showcard 5.4 drinks consumed: ____”  1.e Number of Showcard 5.5 drinks consumed: └──┘.└─┴─┘  1.e.total: Amount in mL of Showcard 5.5 drinks consumed: ____”  1.f Number of Showcard 5.6 drinks consumed:└──┘.└─┴─┘  1.f.total: Amount in mL of Showcard 5.6 drinks consumed: ____” | DIET_1t1a,1b..2a,  2b,..  diet_1t1a_total, diet_1t1b_total, diet_1t1c_total,…. | |
|  |  | How many servings of **sugar-sweetened fizzy drinks** (sodas) do you drink on a typical day? | | inst_diet2b | |
|  |  | (SHOWCARD 5)  *Enter the number of drinks consumed per category as shown in the show card.*  Don’t know 777 | 2.a Number of Showcard 5.1 drinks consumed: └──┘.└─┴─┘  2.a.total: Amount in mL of Showcard 5.1 drinks consumed: ____”  2.b Number of Showcard 5.2 drinks consumed: └──┘.└─┴─┘  2.b.total: Amount in mL of Showcard 5.2 drinks consumed: ____”  2.c Number of Showcard 5.3 drinks consumed: └──┘.└─┴─┘  2.c.total: Amount in mL of Showcard 5.3 drinks consumed: ____”  2.d Number of Showcard 5.4 drinks consumed: └──┘.└─┴─┘  2.d.total: Amount in mL of Showcard 5.4 drinks consumed: ____”  2.e Number of Showcard 5.5 drinks consumed: └──┘.└─┴─┘  2.e.total: Amount in mL of Showcard 5.5 drinks consumed: ____”  2.f Number of Showcard 5.6 drinks consumed:└──┘.└─┴─┘  2.f.total: Amount in mL of Showcard 5.6 drinks consumed: ____” | DIET_1t2a,2b,2c,  2d,..  diet_1t2a_total, diet_1t2b_total, diet_1t2c_total,…. | |

| **SECTION 6: BEHAVIOURAL MEASURES – PHYSICAL ACTIVITY** | | | |
| --- | --- | --- | --- |
| **S.NO:** | **Question** | **Response** | **Code** |
| Please think about the last ONE MONTH and the last SEVEN DAYS when you answer these questions. | | | |
| A | On how many of the last SEVEN DAYS did you do **vigorous activities** for **at least 15 minutes**, such as cycling uphill or at fast pace; swimming laps; carrying heavy loads; shovelling or digging; jogging; running or a sport?  0 1 2 3 4 5 6 7 | └──┘ | PA_1a |
| B | On how many of the last SEVEN DAYS did you do **moderate activities** for **at least 30 minutes**, such as recreational swimming; gardening; heavy cleaning such as washing windows, vacuuming, sweeping or mopping; brisk walking; biking at moderate pace; etc.?  0 1 2 3 4 5 6 7 | └──┘ | PA_1b |

| **SECTION 7: BEHAVIOURAL MEASURES – TOBACCO USE** | | | |
| --- | --- | --- | --- |
| **S.NO:** | **Question** | **Response** | **Code** |
| A | Have you used any tobacco products, such as cigarettes, cigars, pipes, chewing tobacco or snuff/snus in the last 7 days?  1. Daily  2. Less than daily  3. Never  **If ‘never’, SKIP to SECTION 8** | **└──┘** | TOB_1a |
| B | During any visit to a doctor or other health worker in the PAST 12 MONTHS, were you **advised to quit** using tobacco?  Yes = 1  No = 0  Zero visits during the past 12 months = 3 | **└──┘** | TOB_1b |

| **SECTION 8: BEHAVIOURAL MEASURES – ALCOHOL CONSUMPTION** | | | |
| --- | --- | --- | --- |
| **S.NO:** | **Question** | **Response** | **Code** |
| A | During the PAST MONTH, **how frequently** have you had at least one standard alcoholic drink? *(USE SHOWCARD 6)*  1 = Daily = 1  2 = 5-6 days per week  3 = 2-4 days per week  4 = 1-2 days per week  5 = 1-3 days per month  6 = Occasionally, usually less than once a month  7 = Never | **└──┘** | ALC_1a |

| **SECTION 9: OTHER BEHAVIOURAL MEASURES – FOOT CARE**  **For participants with pre-diabetes, SKIP to SECTION 10** | | | |
| --- | --- | --- | --- |
| **S.NO:** | **Question** | **Response** | **Code** |
| A | On how many of the last SEVEN DAYS did you check your feet for sore/ulcers?  0 1 2 3 4 5 6 7 | **└──┘** | FOOT_1a |

| **SECTION 10: BIOCHEMICAL** | | | |
| --- | --- | --- | --- |
| **S.NO:** | **Question** | **Response** | **Code** |
| **BIOCHEMICAL – FPG** | | | |
| A | During the past 12 hours have you had anything to eat or drink other than plain water  Yes = 1  No = 0  *(To be asked when collecting FPG for diagnosis/recruitment)* | **└──┘** | FPG_1a |
|  | If yes, do not proceed |  | inst_fpg1 |
| B | Choose the units appropriate for the device measuring the fasting blood glucose  *(Essential that fasting sample collected, do not proceed if participant has not fasted)* | 1. mmol/L  2. mg/dL | FPG_1b_opt |
|  | Fasting blood glucose measure (mmol/L) | └─┴─┘**. └─┘**mmol/L | FPG_1b |
|  | Fasting blood glucose measure (mg/dL) | └─┴─┴─┘**. └─┘**mg/dL | FPG_1b_units |
| **BIOCHEMICAL – HBA1C** | | | |
| A | Choose the units appropriate for the device measuring the  glycated haemoglobin concentration Test 1  (% or mmol/m) | 1. Percent (%)  2. mmol/m | hba1c_1a_opt |
|  | Glycated haemoglobin concentration Test 1 (%) | └─┴─┘**. └─┘**% | HBA1C_1a |
|  | Glycated haemoglobin concentration Test 1 (mmol/m) | └─┴─┴─┘**.└─┘**mmol/mol | HBA1C_1a_units |

| **SECTION 11: ANTHROPOMETRY AND OTHERS: HEIGHT, WEIGHT & WAIST CIRCUMFERENCE** | | | |
| --- | --- | --- | --- |
| **S.NO:** | **Question** | **Response** | **Code** |
| **Section 11.1: ANTHROPOMETRY** | | | |
| A | Height | in Centimetres (cm) └─┴─┴─┘ | HT_1 |
| B | Weight | in Kilograms (kg) └─┴─┴─┘.└─┘ | WT_1 |
| C | Waist circumference | in Centimetres (cm) └─┴─┴─┘.└─┘ | WC_1 |
| **Section 11.2: BLOOD PRESSURE (SITTING)**  *(Spread the 3 measurements throughout the total time used for the data collection of this patient – usually with a 10-min gap in-between)* | | | |
| A | Blood pressure, reading 1 | Systolic (mmHg) └─┴─┴─┘ | SBP_1a |
|  |  | Diastolic (mmHg) └─┴─┴─┘ | DBP_1a |
| B | Blood pressure, reading 2 | Systolic (mmHg) └─┴─┴─┘ | SBP_1b |
|  |  | Diastolic (mmHg) └─┴─┴─┘ | DBP_1b |
| C | Blood pressure, reading 3 | Systolic (mmHg) └─┴─┴─┘ | SBP_1c |
|  |  | Diastolic (mmHg) └─┴─┴─┘ | DBP_1c |
| **Section 11.3: 7-DAY PEDOMETER STEPS**  ***(****Blind the number of steps displayed by the pedometer using a masking tape.*  *Separate procedure about pedometers in each country to be made by country teams:*  *Clear instruction about pedometer at the time of handing out to participants: only complete days, wear it the whole day!*  *If not complete 7 days: still write down the measurements. Re-do only if participant is willing.*   - *Number of minimum complete days required (5?) – to be considered.* | | | |
| A | Period of pedometer recording  *(Enter only completed days)* | Start date: DD/MM └─┴─┘/└─┴─┘  End date: DD/MM └─┴─┘/└─┴─┘ | PED_1a_start  PED_1a_end |
| B | Total pedometer steps recorded by the device for the period | └─┴─┴─┴─┴─┘ | PED_1b |

**BREAK data collection HERE, according to instructions on page 5**

PART II: Intervention process measures (mainly based on Self Determination Theory)

The broad principles of the intervention are based on Self-determination theory (SDT). Therefore, the evaluation of theoretical components is also based on the SDT framework. Figure 1 shows a simplified version of the SDT framework. This section of the document contains tools to measure each of these components.

**
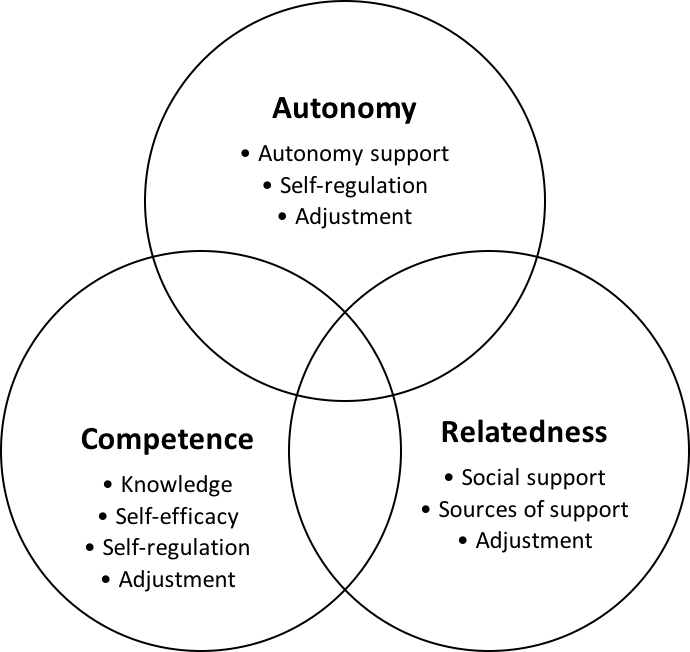
**

*Figure 1: Components of SDT as measured by the evaluation tools in this section*

**Table 2: Details of tools, sources and data collection points for Part II**

| Questionnaires | Source | BL | M2-11 | EL |
| --- | --- | --- | --- | --- |
| **Social Support** | GACD diabetes data dictionary | X |  | X |
| **Sources of support** | Kerala Diabetes Prevention Programme (KDPP) | X |  | X |
| **Self-efficacy** | Pilvikki et al: The perceived self-efficacy scale  <http://www.ncbi.nlm.nih.gov/pubmed/23740266> | X |  | X |
| **Autonomy support** | Health care climate questionnaire  (see website self-determination theory) | X |  | X |
| **Self-regulation** | Treatment Self-Regulation Questionnaire (TSRQ)  (see website self-determination theory) | X |  | X |
| **Psychological Adjustment** | Torres et al: <http://www.ncbi.nlm.nih.gov/pubmed/16341399>  Rodrigues et al: <http://www.ncbi.nlm.nih.gov/pubmed/19820852> | X |  | X |
| **Quality of Life & Stress*** | - GACD diabetes data dictionary  - The 2-item Diabetes Distress Screening Scale | X |  | X |
| **Treatment Satisfaction*** | SMART2D | X |  | X |
| **Knowledge about diabetes** | Garcia et al: <http://care.diabetesjournals.org/content/24/1/16> | X |  | X |

*** The quality of life and stress and Diabetes treatment satisfaction questionnaires do not belong with SDT. However, they do measure process outcomes and use the Likert scale responses. We therefore felt that participants will be able to respond easier in this section as responses are along similar lines to the other SDT-related tools.

| **SECTION 12: SOCIAL SUPPORT** | | | |
| --- | --- | --- | --- |
| **S.No:** | **Questions** | **Response** | **Code** |
| A | Intro: **We want to understand to what extent people close to you (friends, family or relatives) have helped you to do physical activity.** | |  |
|  | How often have people close to you (friends, family or relatives) **exercised with you**?  1= Never  2= less than once a week  3= once a week  4= more than once a week  888= no answer/not applicable | └──┘ | SUP_a_exercised |
|  | How often have people close to you (friends, family or relatives) **encouraged you to exercise**?  1= Never  2= less than once a week  3= once a week  4= more than once a week  888= no answer/not applicable | └──┘ | SUP_a_encouraged |
|  | How often have people close to you (friends, family or relatives) **changed their schedule so you could exercise together**?  1= Never  2= less than once a week  3= once a week  4= more than once a week  888= no answer/not applicable | └──┘ | SUP_a_changed |
|  | How often have people close to you (friends, family or relatives) **discussed exercising with you**?  1= Never  2= less than once a week  3= once a week  4= more than once a week  888= no answer/not applicable | └──┘ | SUP_a_discussed |
|  | How often have people close to you (friends, family or relatives) **helped you with exercising on special occasions such as holidays, feasts, family gatherings**?  1= Never  2= less than once a week  3= once a week  4= more than once a week  888= no answer/not applicable | └──┘ | SUP_a_planned |
| B | Intro: **We want to understand to what extent people close to you (friends, family or relatives) have helped you to maintain a healthy diet.** | |  |
|  | How often have people close to you (friends, family or relatives) **eaten healthy food with you**?  1= Never  2= less than once a week  3= once a week  4= more than once a week  888= no answer/not applicable | └──┘ | SUP_b_eaten |
|  | How often have people close to you (friends, family or relatives) **encouraged you to stick with your healthy diet**?  1= Never  2= less than once a week  3= once a week  4= more than once a week  888= no answer/not applicable | └──┘ | SUP_b_encouraged |
|  | How often have people close to you (friends, family or relatives) **changed their eating habits into healthier so you could eat the same foods**?  1= Never  2= less than once a week  3= once a week  4= more than once a week  888= no answer/not applicable | └──┘ | SUP_b_changed |
|  | How often have people close to you (friends, family or relatives) **discussed healthy eating with you**?  1= Never  2= less than once a week  3= once a week  4= more than once a week  888= no answer/not applicable | └──┘ | SUP_b_discussed |
|  | How often have people close to you (friends, family or relatives) **helped you with eating healthy food on special occasions such as holidays, feasts, family gatherings**?  1= Never  2= less than once a week  3= once a week  4= more than once a week  888= no answer/not applicable | └──┘ | SUP_b_planned |

| **SECTION 13: SOURCES of SUPPORT / ENCOURAGEMENT** | | | | |
| --- | --- | --- | --- | --- |
| **Instruction for data collectors on LIKERT scales:**  In this section, the 2 step approach needs to be used:  **Give SHOWCARD 7 to the participant to help them through the responses**  Ask the question in two parts:   1. Ask the participant the specific question as written below 🡪Participant answers: Yes/neutral/no   Data collector asks: ‘do you strongly or moderately (dis)agree’ | | | inst_sos1 | |
|  | **Sources of Support** | **Response** | | **Code** |
| **For participants with pre-diabetes, SKIP to B.** | | | | |
| A | Intro: We want to know about different groups of people if they are helping you with your diabetes management. Diabetes management can include various things like taking medication, helping with exercise, maintaining a healthy diet, accompanying you to the health centre, etc.  *Clarification for data-collectors: The meaning of “help” relates to encouragement, information sharing, sharing experiences, etc..*  *Instructions:’ Not applicable’ to be used only for the following items, if they are not available at the time of the survey: peer leaders, peer groups, community link team (SA: CHW) and care companions.* | | |  |
|  | Do **family members** help you with your diabetes management?  1 Strongly disagree  2 Disagree  3 Neutral  4 Agree  5 Strongly agree  888 Not applicable | └──┘ | | SOS_1a_family |
|  | Do **friends** help you with your diabetes management?  1 Strongly disagree  2 Disagree  3 Neutral  4 Agree  5 Strongly agree  888 Not applicable | └──┘ | | SOS_1a_friends |
|  | Does the **health centre** help you with your diabetes management?  1 Strongly disagree  2 Disagree  3 Neutral  4 Agree  5 Strongly agree  888 Not applicable | └──┘ | | SOS_1a_hc |
| **For participants with diabetes, SKIP to Section 14** | | | | |
| B | Intro: We want to know about different groups of people if they are helping you with maintaining a healthier lifestyle. A healthier lifestyle can include various things like helping you to exercise, eat well, stop smoking, etc.  *Clarification for data-collectors: The meaning of “help” relates to encouragement, information sharing, sharing experiences, etc..*  *~~Instructions:’ Not applicable’ to be used only for the following items, if they are not available at the time of the survey: peer leaders, peer groups, community link team (SA: CHW) and care companions.~~* | | |  |
|  | Do **family members** help you with your healthy lifestyle such as exercising and eating well?  1 Strongly disagree  2 Disagree  3 Neutral  4 Agree  5 Strongly agree  888 Not applicable | └──┘ | | SOS_1b_family |
|  | Do **friends** help you with your healthy lifestyle such as exercising and eating well?  1 Strongly disagree  2 Disagree  3 Neutral  4 Agree  5 Strongly agree  888 Not applicable | └──┘ | | SOS_1b_friends |
|  | Does the **health centre** help you with your healthy lifestyle such as exercising and eating well?  1 Strongly disagree  2 Disagree  3 Neutral  4 Agree  5 Strongly agree  888 Not applicable | └──┘ | | SOS_1b_hc |

| **SECTION 14: SELF-EFFICACY** | | | |
| --- | --- | --- | --- |
| **Instruction for data collectors on LIKERT scales:**  In this section, the 2 step approach needs to be used:  **Give SHOWCARD 7 to the participant to help them through the responses**  Ask the question in two parts:   1. Ask the participant the specific question as written below 🡪Participant answers: Yes/neutral/no   Data collector asks: ‘do you strongly or moderately (dis)agree’ | | | inst_eff1 |
| Intro: **We want to know if you can maintain a healthy diet ~~and do physical activity~~ under specific circumstances.** | | | |
| **S.NO:** | **Question** | **Response** | **Code** |
| A | Do you think you can maintain a healthy diet **even if you need to change how you cook at home**?  1 Strongly disagree  2 Disagree  3 Neutral  4 Agree  5 Strongly agree  888 Not applicable | └──┘ | EFF_1a |
| B | Do you think you can maintain a healthy diet **even if you are not used to the taste of these foods**?  1 Strongly disagree  2 Disagree  3 Neutral  4 Agree  5 Strongly agree  888 Not applicable | └──┘ | EFF_1b |
| C | Do you think you can maintain a healthy diet **even if this means that you have to eat less**?  1 Strongly disagree  2 Disagree  3 Neutral  4 Agree  5 Strongly agree  888 Not applicable | └──┘ | EFF_1c |
| D | Do you think you can maintain a healthy diet **even if your family or friends don’t help you**?  1 Strongly disagree  2 Disagree  3 Neutral  4 Agree  5 Strongly agree  888 Not applicable | └──┘ | EFF_1d |
| E | Do you think you can maintain a healthy **diet even if you would have to pay more for it**?  1 Strongly disagree  2 Disagree  3 Neutral  4 Agree  5 Strongly agree  888 Not applicable | └──┘ | EFF_1e |
| F | Do you think you can maintain a healthy diet **even if others around you eat unhealthy food**?  1 Strongly disagree  2 Disagree  3 Neutral  4 Agree  5 Strongly agree  888 Not applicable | └──┘ | EFF_1f |
| **We want to know if you can ~~maintain a healthy diet and~~ do physical activity under specific circumstances.** | | | |
| G | Do you think you can be physically active **even during holidays, weddings or other special events**?  1 Strongly disagree  2 Disagree  3 Neutral  4 Agree  5 Strongly agree  888 Not applicable | └──┘ | EFF_1g |
| H | Do you think you can be physically active **even if your family does not encourage you to be physically activity**?  1 Strongly disagree  2 Disagree  3 Neutral  4 Agree  5 Strongly agree  888 Not applicable | └──┘ | EFF_1h |
| I | Do you think you can be physically active **even if you think it is not the best weather for doing sports**?  1 Strongly disagree  2 Disagree  3 Neutral  4 Agree  5 Strongly agree  888 Not applicable | └──┘ | EFF_1i |
| J | Do you think you can be physically active **even if you are in a place with no exercise facilities or safe roads for walking**?  (probing ‘for instance at home or at work or in another way’)    1 Strongly disagree  2 Disagree  3 Neutral  4 Agree  5 Strongly agree  888 Not applicable | └──┘ | EFF_1j |
| K | Do you think you can be physically active **even if you have health problems?**  1 Strongly disagree  2 Disagree  3 Neutral  4 Agree  5 Strongly agree  888 Not applicable | └──┘ | EFF_1k |
| L | Do you think you can be physically active **even if no other people around you are doing exercise or walking**?  1 Strongly disagree  2 Disagree  3 Neutral  4 Agree  5 Strongly agree  888 Not applicable | └──┘ | EFF_1l |

| **SECTION 15: AUTONOMY SUPPORT** | |
| --- | --- |
| **Instruction for data collectors on LIKERT scales:**  In this section, the 2 step approach needs to be used:  **Give SHOWCARD 7 to the participant to help them through the responses**  Ask the question in two parts:   1. Ask the participant the specific question as written below 🡪Participant answers: Yes/neutral/no   Data collector asks: ‘do you strongly or moderately (dis)agree’ | inst_aus1 |
| Intro: We want to know how the doctors and nurses working at the health centre or clinic treat you regarding your diabetes/pre-diabetes management. Diabetes management can include various things like taking medication, exercising, following a healthy diet, etc. Pre-diabetes management can include various things like exercising, following a healthy diet, etc.  Please respond according to the condition you have, either diabetes or pre-diabetes: | |

| **S.NO** | **Question** | **Response** | **Code** |
| --- | --- | --- | --- |
| A | Do you feel that the nurses and doctors at the health centre or clinic **give you choices and options *about your diabetes / pre-diabetes management****?*  1 Strongly disagree  2 Disagree  3 Neutral  4 Agree  5 Strongly agree  888 Not applicable | └──┘ | AUS_1a |

| B | Do you feel that the nurses and doctors at the health centre or clinic **understand how you see things *regarding* *your diabetes / pre-diabetes management****?*  1 Strongly disagree  2 Disagree  3 Neutral  4 Agree  5 Strongly agree  888 Not applicable | └──┘ | AUS_1b |
| --- | --- | --- | --- |
| C | Do you feel that the nurses and doctors at the health centre or clinic **make you feel able to change *your diabetes / pre-diabetes management****?*  1 Strongly disagree  2 Disagree  3 Neutral  4 Agree  5 Strongly agree  888 Not applicable | └──┘ | AUS_1c |
| D | Do you feel that the nurses and doctors at the health centre or clinic **listen to how you would like to do things regarding *your diabetes / pre-diabetes management***?  1 Strongly disagree  2 Disagree  3 Neutral  4 Agree  5 Strongly agree  888 Not applicable | └──┘ | AUS_1d |
| E | Do you feel that the nurses and doctors at the health centre or clinic **encourage you to ask questions about *your diabetes / pre-diabetes management***?  1 Strongly disagree  2 Disagree  3 Neutral  4 Agree  5 Strongly agree  888 Not applicable | └──┘ | AUS_1e |
| F | Do you feel that the nurses and doctors at the health centre or clinic **try to understand how you see *your diabetes / pre-diabetes management,* before suggesting a different treatment**?  1 Strongly disagree  2 Disagree  3 Neutral  4 Agree  5 Strongly agree  888 Not applicable | └──┘ | AUS_1f |

| **SECTION 16: SELF-REGULATION** | | | |
| --- | --- | --- | --- |
| **Instruction for data collectors on LIKERT scales:**  In this section, the 2 step approach needs to be used:  **Give SHOWCARD 7 to the participant to help them through the responses**  Ask the question in two parts:   1. Ask the participant the specific question as written below 🡪Participant answers: Yes/neutral/no   Data collector asks: ‘do you strongly or moderately (dis)agree’ | | | inst_sreg1 |
| **S.No.** | **Question** | **Response** | **Code** |
|  | Intro: Think for a moment about the **reasons** why you actually would follow a healthy diet: why would you do this?  (*Instructions: give the patient a couple of seconds to think about it*).  We will now present possible reasons why you may follow a healthy diet. Please agree or disagree, using the options provided. | |  |
| A | Would you maintain a healthy diet **because** **you personally believe it is the best thing for your health**?  1 Strongly disagree  2 Disagree  3 Neutral  4 Agree  5 Strongly agree  888 Not applicable | └──┘ | SREG_1a |
| B | Would you maintain a healthy diet **because** **you'd feel bad about yourself if you didn't**?  1 Strongly disagree  2 Disagree  3 Neutral  4 Agree  5 Strongly agree  888 Not applicable | └──┘ | SREG_1b |
| C | Would you maintain a healthy diet **because** **you feel pressure from others to do it?**  1 Strongly disagree  2 Disagree  3 Neutral  4 Agree  5 Strongly agree  888 Not applicable | └──┘ | SREG_1c |
| D | Would you maintain a healthy diet **because** **others would be upset with you if you didn't**?  1 Strongly disagree  2 Disagree  3 Neutral  4 Agree  5 Strongly agree  888 Not applicable | └──┘ | SREG_1d |
| E | Would you maintain a healthy diet **because** **it is very important for being as healthy as possible**?  1 Strongly disagree  2 Disagree  3 Neutral  4 Agree  5 Strongly agree  888 Not applicable | └──┘ | SREG_1e |
| F | Would you maintain a healthy diet **because** **you would feel guilty or ashamed of yourself if you didn’t**?  1 Strongly disagree  2 Disagree  3 Neutral  4 Agree  5 Strongly agree  888 Not applicable | └──┘ | SREG_1f |
| G | Would you maintain a healthy diet **because** **you feel that you want to take responsibility for your own health**?  1 Strongly disagree  2 Disagree  3 Neutral  4 Agree  5 Strongly agree  888 Not applicable | └──┘ | SREG_1g |
| H | Would you maintain a healthy diet **because it is an important choice you really want to make**?  1 Strongly disagree  2 Disagree  3 Neutral  4 Agree  5 Strongly agree  888 Not applicable | └──┘ | SREG_1h |
|  | Intro: Think for a moment about the **reasons** why you actually would do physical exercise: why would you do this?  (*Instructions: give the patient a couple of seconds to think about it*).  We will now present possible reasons why you may do physical exercise. Please agree or disagree, using the options provided. | |  |
| I | Would you do physical exercise **because** **you personally believe it is the best thing for your health**?  1 Strongly disagree  2 Disagree  3 Neutral  4 Agree  5 Strongly agree  888 Not applicable | └──┘ | SREG_1i |
| J | Would you do physical exercise **because** **you'd feel bad about yourself if you didn't**?  1 Strongly disagree  2 Disagree  3 Neutral  4 Agree  5 Strongly agree  888 Not applicable | └──┘ | SREG_1j |
| K | Would you do physical exercise **because** **you feel pressure from others to do it**?  1 Strongly disagree  2 Disagree  3 Neutral  4 Agree  5 Strongly agree  888 Not applicable | └──┘ | SREG_1k |
| L | Would you do physical exercise **because** **others would be upset with you if you didn't**?  1 Strongly disagree  2 Disagree  3 Neutral  4 Agree  5 Strongly agree  888 Not applicable | └──┘ | SREG_1l |
| M | Would you do physical exercise **because it is very important for being as healthy as possible**?  1 Strongly disagree  2 Disagree  3 Neutral  4 Agree  5 Strongly agree  888 Not applicable | └──┘ | SREG_1m |
| N | Would you do physical exercise **because** **you would feel guilty or ashamed of yourself if you didn’t**?  1 Strongly disagree  2 Disagree  3 Neutral  4 Agree  5 Strongly agree  888 Not applicable | └──┘ | SREG_1n |
| O | Would you do physical exercise **because you feel that you want to take responsibility for your own health**?  1 Strongly disagree  2 Disagree  3 Neutral  4 Agree  5 Strongly agree  888 Not applicable | └──┘ | SREG_1o |
| P | Would you do physical exercise **because** **it is an important choice you really want to make**?  1 Strongly disagree  2 Disagree  3 Neutral  4 Agree  5 Strongly agree  888 Not applicable | └──┘ | SREG_1p |

| **SECTION 17: PSYCHOLOGICAL ADJUSTMENT** | | | |
| --- | --- | --- | --- |
|  | **Instruction for data collectors on LIKERT scales:**  In this section, the 2 step approach needs to be used:  **Give SHOWCARD 7 to the participant to help them through the responses**  Ask the question in two parts:   1. Ask the participant the specific question as written below 🡪Participant answers: Yes/neutral/no   Data collector asks: ‘do you strongly or moderately (dis)agree’ | | inst_psa1 |
|  | Intro: **We want to explore how you feel about having pre-diabetes / diabetes. Please respond in relation to the condition you have, i.e. either diabetes or pre-diabetes using the options provided.** | | |
| A | Do you think that pre-diabetes / diabetes is the worst thing that has ever happened to you?  1 Strongly disagree  2 Disagree  3 Neutral  4 Agree  5 Strongly agree  888 Not applicable | └──┘ | PSA_1a |
| B | Do you often feel embarrassed about having pre-diabetes / diabetes?  1 Strongly disagree  2 Disagree  3 Neutral  4 Agree  5 Strongly agree  888 Not applicable | └──┘ | PSA_1b |
| C | Do you think there is little hope of leading a normal life with pre-diabetes / diabetes?  1 Strongly disagree  2 Disagree  3 Neutral  4 Agree  5 Strongly agree  888 Not applicable | └──┘ | PSA_1c |
| D | Do you think the proper control of pre-diabetes / diabetes involves a lot of sacrifice and inconvenience?  1 Strongly disagree  2 Disagree  3 Neutral  4 Agree  5 Strongly agree  888 Not applicable | └──┘ | PSA_1d |
| E | Do you try not to let people know about your pre-diabetes / diabetes?  1 Strongly disagree  2 Disagree  3 Neutral  4 Agree  5 Strongly agree  888 Not applicable | └──┘ | PSA_1e |
| F | Do you think pre-diabetes / diabetes is not really a problem because it can be controlled?  1 Strongly disagree  2 Disagree  3 Neutral  4 Agree  5 Strongly agree  888 Not applicable | └──┘ | PSA_1f |

| **SECTION 18: TREATMENT SATISFACTION** | | | | |
| --- | --- | --- | --- | --- |
| **Instruction for data collectors on LIKERT scales:**  In this section, the 2 step approach needs to be used:  **Give SHOWCARD 7 to the participant to help them through the responses**  Ask the question in two parts:   1. Ask the participant the specific question as written below 🡪Participant answers: Yes/neutral/no   Data collector asks: ‘do you strongly or moderately (dis)agree’ | | | | inst_ts1 |
| The following question relates to your experience regarding the treatment and follow-up of your **diabetes / pre-diabetes** | | | | |
| **S.NO:** | **Questions** | **Response** | **Code** | |
| A | Are you satisfied with the services you received at the health center?  1 Strongly disagree  2 Disagree  3 Neutral  4 Agree  5 Strongly agree  888 Not applicable | **└──┘** | TS_1a | |

| **SECTION 19: QUALITY OF LIFE & STRESS** | | | |
| --- | --- | --- | --- |
| **S.NO:** | **Questions** | **Response** | **Code** |
| A | At this point of time in your life, consider your home situation, relationships, finances, work situation and other aspects of your life, how would you describe the quality of your life overall?  Excellent = 1  Good = 2  Fair = 3  Poor = 4  Don’t know/neutral/unsure = 777 | **└──┘** | QOL_1a |
| B | Which of these best describe your health?  Excellent = 1  Good = 2  Fair = 3  Poor = 4  Don’t know/neutral/unsure = 777 | **└──┘** | QOL_1b |
| C | 1. Are you currently facing any serious on-going problems that affects your health?  Yes = 1  No = 0  **If NO, SKIP to D** | **└──┘** | QOL_1c1 |
|  | 2. If YES, would you say this problem has been…  1 = Not very stressful  2 = Moderately stressful  3 = Very stressful | **└──┘** | QOL_1c2 |
|  | 3. Has this been a problem for 12 months or more?  Yes = 1  No = 0 | **└──┘** | QOL_1c3 |
| D | 1. Are you currently facing any serious on-going problem that affects your job or ability to work (including housework)?  Yes = 1  No = 0  **IF NO, SKIP to E** | **└──┘** | QOL_1d1 |
|  | 2. If YES, would you say this problem has been…  1 = Not very stressful  2 = Moderately stressful  3 = Very stressful | **└──┘** | QOL_1d2 |
|  | 3. Has this been a problem for 12 months or more?  Yes = 1  No = 0 | **└──┘** | QOL_1d3 |
| E | 1. Are you currently facing any on-going financial strain/stress or difficulties?  Yes = 1  No = 0 | **└──┘** | QOL_1e1 |
|  | 2. If YES, would you say this problem has been…  1 = Not very stressful  2 = Moderately stressful  3 = Very stressful | **└──┘** | QOL_1e2 |
|  | 3. Has this been a problem for 12 months or more?  Yes = 1  No = 0 | **└──┘** | QOL_1e3 |
| F | Are you currently facing any on-going difficulties in a relationship with someone close to you?  Yes = 1  No = 0 | **└──┘** | QOL_1f1 |
|  | 2. If YES, would you say this problem has been…  1 = Not very stressful  2 = Moderately stressful  3 = Very stressful | **└──┘** | QOL_1f2 |
|  | 3. Has this been a problem for 12 months or more?  Yes = 1  No = 0 | **└──┘** | QOL_1f3 |
|  | **Do any of the following currently cause stress in your life?**  ~~1 Yes~~  ~~2 No~~  ~~3 Don’t know~~ | |  |
| ~~L~~ G | Social isolation  1 Yes  0 No  777 Don’t know | **└──┘** | QOL_1g |
| ~~M~~ H | Lack of education  1 Yes  0 No  777 Don’t know | **└──┘** | QOL_1h |
| ~~N~~ I | Unemployment  1 Yes  0 No  777 Don’t know | **└──┘** | QOL_1i |
| ~~O~~ J | Family problems  1 Yes  0 No  777 Don’t know | **└──┘** | QOL_1j |
| ~~P~~ K | Marriage/Relationships  1 Yes  0 No  777 Don’t know | **└──┘** | QOL_1k |

| **SECTION 20: KNOWLEDGE ABOUT DIABETES** | | | |
| --- | --- | --- | --- |
| **S.No.** | **Questions** | **Response** | **Code** |
| A | If someone has diabetes, that persons’ children have a higher chance of getting diabetes  1 Yes  0 No  777 Don’t know | └──┘ | KNOW_1a |
| B | Regular exercise will decrease the need for insulin or other diabetic medication  1 Yes  0 No  777 Don’t know | └──┘ | KNOW_1b |
| C | People with diabetes should take extra care when cutting their toenails  1 Yes  0 No  777 Don’t know | └──┘ | KNOW_1c |
| D | Diabetes can cause loss of feeling in my hands, fingers and feet  1 Yes  0 No  777 Don’t know | └──┘ | KNOW_1d |
| E | A diabetic diet consists mostly of special foods which are difficult to obtain  1 Yes  0 No  777 Don’t know | └──┘ | KNOW_1e |
| F | Diabetes can damage the kidneys  1 Yes  0 No  777 Don’t know | └──┘ | KNOW_1f |
| G | Frequent urination and thirst are typical signs of low blood sugar  1 Yes  0 No  777 Don’t know | └──┘ | KNOW_1g |
| H | Diabetes is a disease that requires lifelong treatment  1 Yes  0 No  777 Don’t know | └──┘ | KNOW_1h |
| I | Taking medication against diabetes is only useful when you have symptoms  1 Yes  0 No  777 Don’t know | └──┘ | KNOW_1i |
| J | Overweight may cause diabetes  1 Yes  0 No  777 Don’t know | └──┘ | KNOW_1j |
| K | An person with uncontrolled diabetes has a higher risk to suffer from a heart disease or a stroke  1 Yes  0 No  777 Don’t know | └──┘ | KNOW_1k |

**For participants with PRE-DIABETES, END HERE!**

PART III: Selected context measures

Context is multi-layered and highly complex, including micro- and macro-components stretching from individual attitudes and beliefs to the wider socio-political climate. We will attempt to measure only those aspects of context which are relevant to the implementation of SMART2D and thereby the outcome as well. The conceptual figure shown below has been modified from Taplin et al and attempts to describe the most relevant layers for SMART2D and the aspects that will be measured using quantitative data collection tools. Figure 2 shows each aspect that will the measured and the corresponding tools *(in italics)*. The grey circles represent areas not covered by these evaluation tools. Some aspects such as lay support networks have been studied to some extent in the formative part and others such as socio-political climate of each setting will be mainly described using narratives to keep track of temporal changes.

*Figure 2: Context measures and corresponding tools*

*
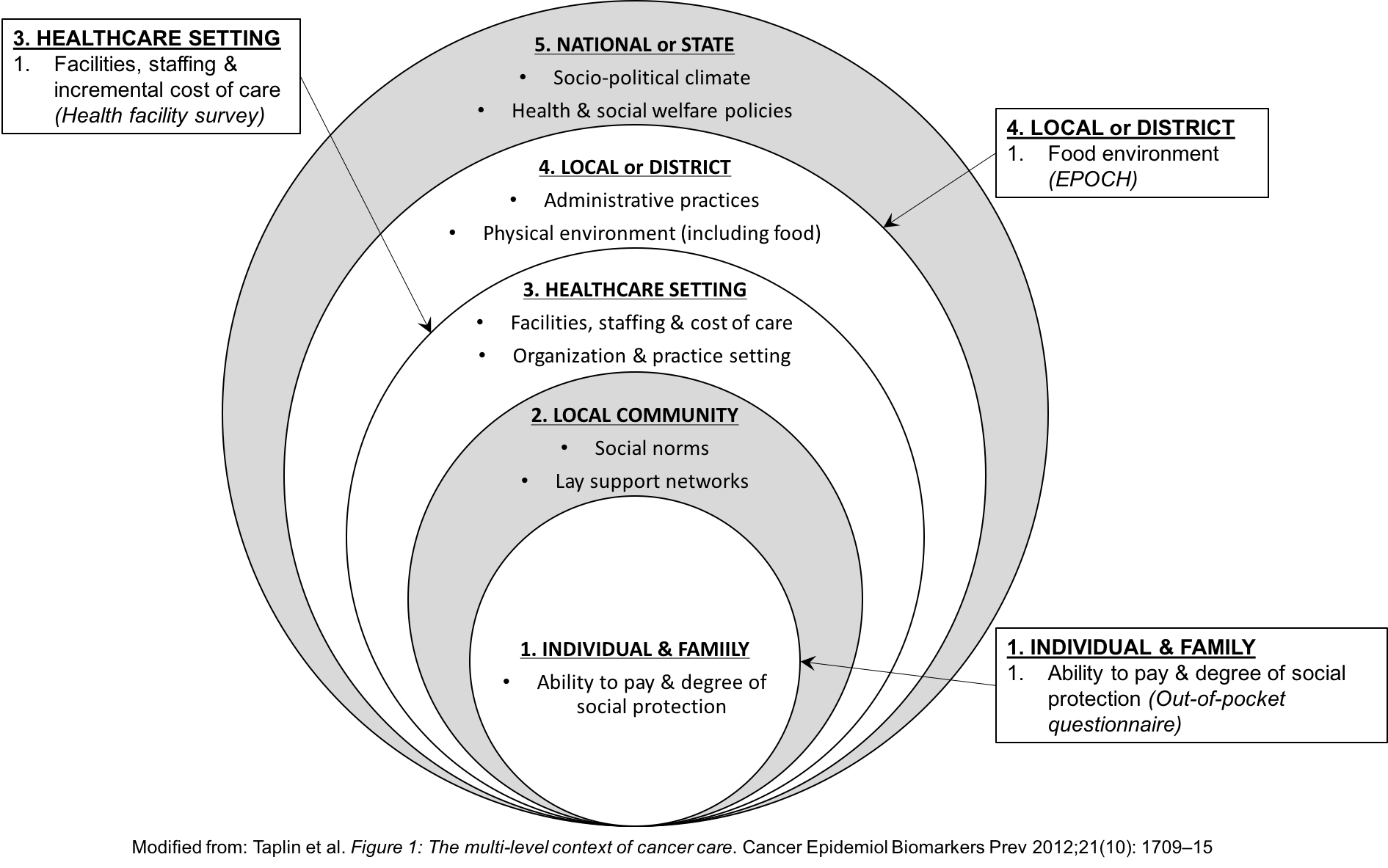
*

**Table 3: Details of tools, sources and data collection points for Part III**

| Questionnaires | Source | BL | M2-11 | EL |
| --- | --- | --- | --- | --- |
| **Out of pocket expenditure: Individual and family questionnaire** | Prepared by Elizabeth Ekirappa | X |  | X |
| **Health facility survey – to be administered to head of health facility*** | Prepared by Elizabeth Ekirappa |  | X |  |
| **EPOCH – to be conducted by researcher and collected one per community*** | Environmental Profile of a Community’s Health (Clara Chow – Population Health Research Institute, Hamilton) |  | X |  |

* Not included in this set of data collection tools

| **SECTION 21: Out of Pocket Expenditure related to type 2 diabetes** | | | |
| --- | --- | --- | --- |
| **SECTION 21A: Out of Pocket Expenditure – Outpatient cost** | | | |
| **S.NO:** | **Questions** | **Response** | **Code** |
| A | Do you usually spend any money on medication and other related costs? | 1 Yes regularly  2 Yes occasionally  0 No  **If NO, SKIP to B** | OOPO_1a |
|  | **In the last month, how much did you spend on the following items in relation to treatment of type 2 diabetes?**  *Please record in respective local currency for each setting: Ug Sh (Uganda)/SAR (South Africa)/SEK (Sweden)* | | |
| B | Medicine:  1. Diabetes medication  2. Other medication | 1. └─┴─┴─┴─┴─┴─┴─┘  2. └─┴─┴─┴─┴─┴─┴─┘ | OOPO_1b1  OOPO_1b2 |
| C | Consultation fees  Not applicable = 888 | └─┴─┴─┴─┴─┴─┴─┘  └──┴──┘ | OOPO_1c |
| D | Laboratory tests  Not applicable = 888 | └─┴─┴─┴─┴─┴─┴─┘  └──┴──┘ | OOPO_1d |
| E | Other supplies  Not applicable = 888 | └─┴─┴─┴─┴─┴─┴─┘  └──┴──┘ | OOPO_1e |
| F | Food while at the health facility  Not applicable = 888 | └─┴─┴─┴─┴─┴─┴─┘  └──┴──┘ | OOPO_1f |
| G | Appreciation of health worker  Not applicable = 888 | └─┴─┴─┴─┴─┴─┴─┘  └──┴──┘ | OOPO_1g |
| H | Transport ( to and from facility)  Not applicable = 888 | └──┴──┘ |  |

O

| **SECTION 21B: Out of Pocket Expenditure – Hospitalisation cost** | | | | | |
| --- | --- | --- | --- | --- | --- |
| I | In the past 12 months, how many times were you hospitalized ? | Number of times └─┴**─┘**  **If 0 SKIP to SECTION 21C** | | | OOPI_1i |
| **For each hospitalization** (max 3 – if more, take last 3) can you answer the following questions | | | | | inst_oopi1 |
|  |  | **1** | **2** | **3** |  |
| J | What was the reason for the hospitalization? | 1. Related to diabetes 2. Unrelated to diabetes   777. I don’t know | 1. Related to diabetes 2. Unrelated to diabetes   777. I don’t know | 1. Related to diabetes 2. Unrelated to diabetes   777. I don’t know | OOPI_1j1  OOPI_1j2  OOPI_1j3 |
| K | How many days were you hospitalised ? | **└──┘** | **└──┘** | **└──┘** | OOPI_1k1  OOPI_1k2  OOPI_1k3 |
| L | What was the total cost of your stay in the hospital? | └─┴─┴─┴─┴─┴─┴─┘ | └─┴─┴─┴─┴─┴─┴─┘ | └─┴─┴─┴─┴─┴─┴─┘ | OOPI_1l1  OOPI_1l2  OOPI_1l3 |
|  | Can you divide the costs for each hospitalisation into the following categories?  *Please record in respective local currency for each setting: Ug Sh (Uganda)/SAR (South Africa)/SEK (Sweden)* | | | | inst_oopi2 |
| M | Cost for medication while in the hospital | └─┴─┴─┴─┴─┴─┴─┘  777. I don’t know | └─┴─┴─┴─┴─┴─┴─┘  777. I don’t know | └─┴─┴─┴─┴─┴─┴─┘  777. I don’t know | OOPI_1m1 OOPI_1m2 OOPI_1m3 |
| N | Payment for accommodation | └─┴─┴─┴─┴─┴─┴─┘  777. I don’t know | └─┴─┴─┴─┴─┴─┴─┘  777. I don’t know | └─┴─┴─┴─┴─┴─┴─┘  777. I don’t know | OOPI_1n1  OOPI_1n2  OOPI_1n3 |
| O | Emergency room fees | └─┴─┴─┴─┴─┴─┴─┘  777. I don’t know | └─┴─┴─┴─┴─┴─┴─┘  777. I don’t know | └─┴─┴─┴─┴─┴─┴─┘  777. I don’t know | OOPI_1o1  OOPI_1o2  OOPI_1o3 |
| P | Consultation (doctor) fees | └─┴─┴─┴─┴─┴─┴─┘  777. I don’t know | └─┴─┴─┴─┴─┴─┴─┘  777. I don’t know | └─┴─┴─┴─┴─┴─┴─┘  777. I don’t know | OOPI_1p1  OOPI_1p2  OOPI_1p3 |
| Q | Surgery | └─┴─┴─┴─┴─┴─┴─┘  777. I don’t know | └─┴─┴─┴─┴─┴─┴─┘  777. I don’t know | └─┴─┴─┴─┴─┴─┴─┘  777. I don’t know | OOPI_1q1  OOPI_1q2  OOPI_1q3 |
| R | Supplies (bandages, syringes, catheters, etc) | └─┴─┴─┴─┴─┴─┴─┘  777. I don’t know | └─┴─┴─┴─┴─┴─┴─┘  777. I don’t know | └─┴─┴─┴─┴─┴─┴─┘  777. I don’t know | OOPI_1r1  OOPI_1r2  OOPI_1r3 |
| S | Laboratory test | └─┴─┴─┴─┴─┴─┴─┘  777. I don’t know | └─┴─┴─┴─┴─┴─┴─┘  777. I don’t know | └─┴─┴─┴─┴─┴─┴─┘  777. I don’t know | OOPI_1s1  OOPI_1s2  OOPI_1s3 |
| T | X-rays, scans etc | └─┴─┴─┴─┴─┴─┴─┘  777. I don’t know | └─┴─┴─┴─┴─┴─┴─┘  777. I don’t know | └─┴─┴─┴─┴─┴─┴─┘  777. I don’t know | OOPI_1t1  OOPI_1t2  OOPI_1t3 |
| U | My own food | └─┴─┴─┴─┴─┴─┴─┘  777. I don’t know | └─┴─┴─┴─┴─┴─┴─┘  777. I don’t know | └─┴─┴─┴─┴─┴─┴─┘  777. I don’t know | OOPI_1u1  OOPI_1u2  OOPI_1u3 |
| V | Ambulance/transport | └─┴─┴─┴─┴─┴─┴─┘  777. I don’t know | └─┴─┴─┴─┴─┴─┴─┘  777. I don’t know | └─┴─┴─┴─┴─┴─┴─┘  777. I don’t know | OOPI_1v1  OOPI_1v2  OOPI_1v3 |
| W | Cost for somebody accompanying me: accommodation | └─┴─┴─┴─┴─┴─┴─┘  777. I don’t know | └─┴─┴─┴─┴─┴─┴─┘  777. I don’t know | └─┴─┴─┴─┴─┴─┴─┘  777. I don’t know | OOPI_1w1  OOPI_1w2  OOPI_1w3 |

| **SECTION 21C: Household Expenditure** | | | |
| --- | --- | --- | --- |
| On average, how much did you spent in your household on the following in the LAST ONE MONTH?” Enter 777 if amount is not known.  *Please record in respective local currency for each setting: Ug Sh (Uganda)/SAR (South Africa)/SEK (Sweden)* | | | |
| A | Food, including such things as rice, meat, fruits, vegetables and cooking oil | └─┴─┴─┴─┴─┴─┴─┘ | HH_1a |
| B | Value of food consumed by the household which is produced/grown in the household | └─┴─┴─┴─┴─┴─┴─┘ | HH_1b |
| C | Gas, electricity, water, telephone and heating fuel | └─┴─┴─┴─┴─┴─┴─┘ | HH_1c |
| D | Transport, repair of vehicles | └─┴─┴─┴─┴─┴─┴─┘ | HH_1d |
| E | Housing rent. If house is not rented, ask about the rental value of patient’s house. | └─┴─┴─┴─┴─┴─┴─┘ | HH_1e |
| F | Education fees and supplies | └─┴─┴─┴─┴─┴─┴─┘ | HH_1f |
| G | Reimbursement of loans | └─┴─┴─┴─┴─┴─┴─┘ | HH_1g |
| H | Insurance premiums or prepaid health plans | └─┴─┴─┴─┴─┴─┴─┘ | HH_1h |
| I | Clothes, shoes and related items | └─┴─┴─┴─┴─┴─┴─┘ | HH_1i |
| J | Goods like washing machines, cooking utensils, stove, radio, furniture, bicycle | └─┴─┴─┴─┴─┴─┴─┘ | HH_1j |
| K | Special occasions including festivals, weddings, funerals, etc | └─┴─┴─┴─┴─┴─┴─┘ | HH_1k |
| L | Others | └─┴─┴─┴─┴─┴─┴─┘ | HH_1l |
| In addition to the above, have you spent any money in the LAST ONE YEAR on any special occasions in your family, illness of other members, purchase of house, car or other assets? 0 = No, 1 = Yes | | | |
| M | Specify |  | HH_1m |
| N | Specify |  | HH_1n |

APPENDIX 1: KISH methodology

**
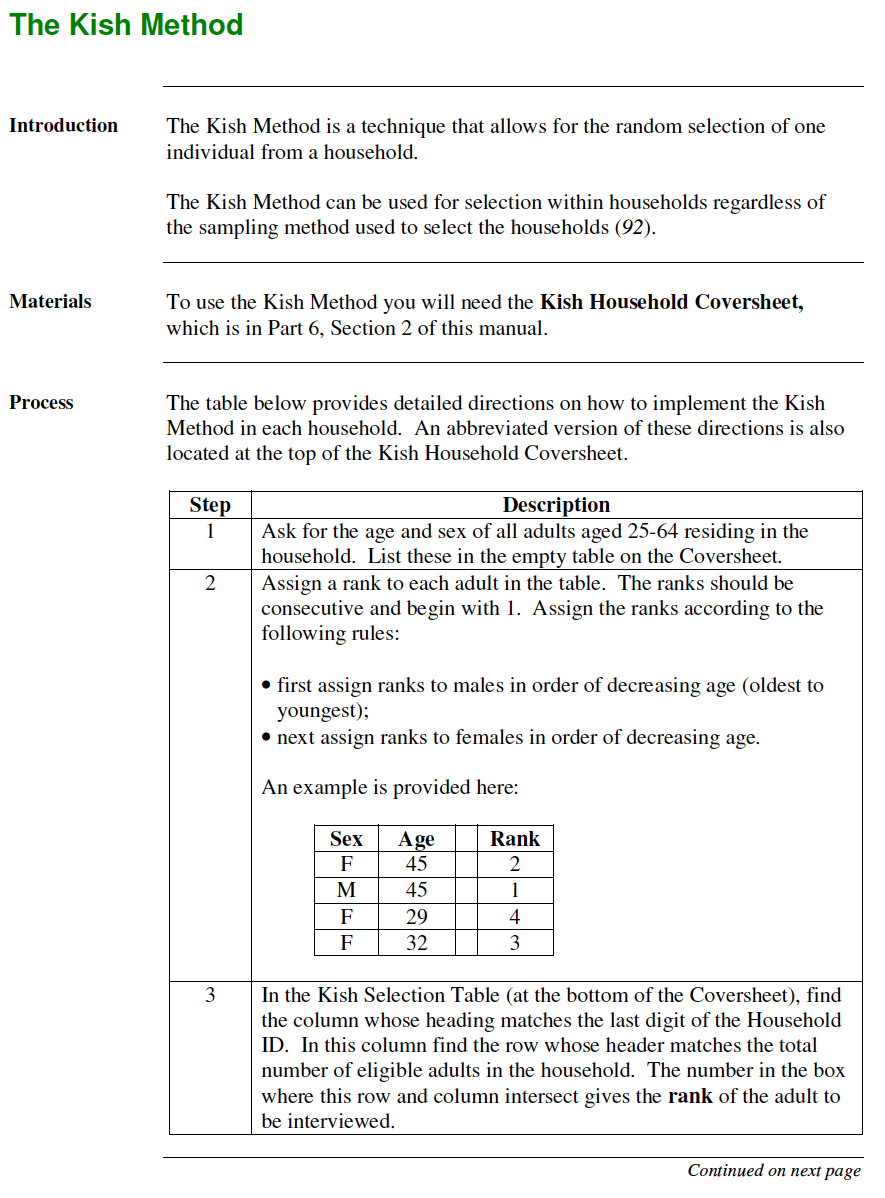
**

**
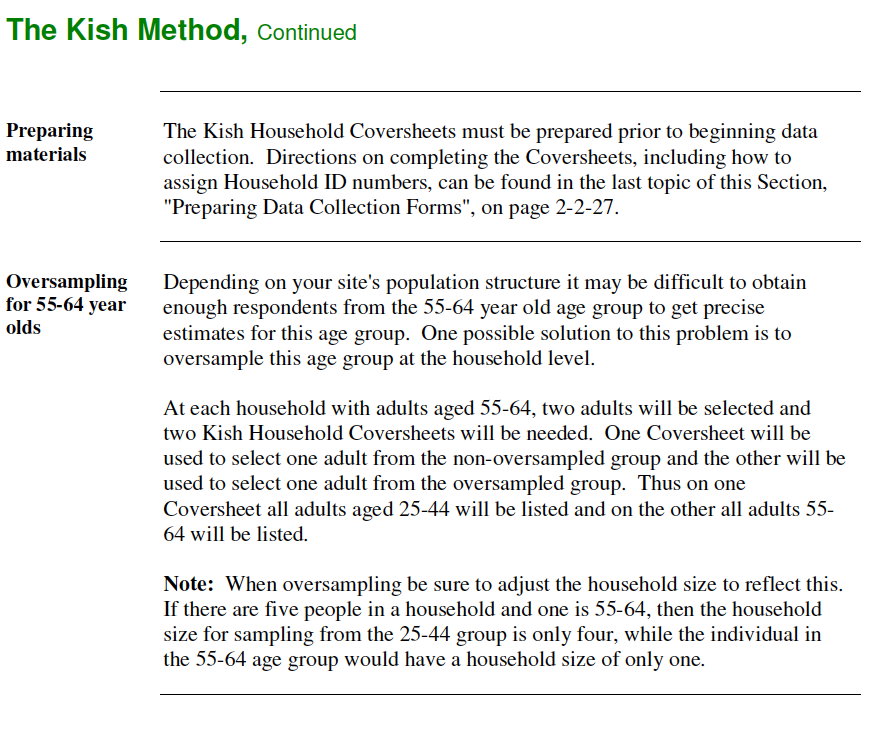
**

**
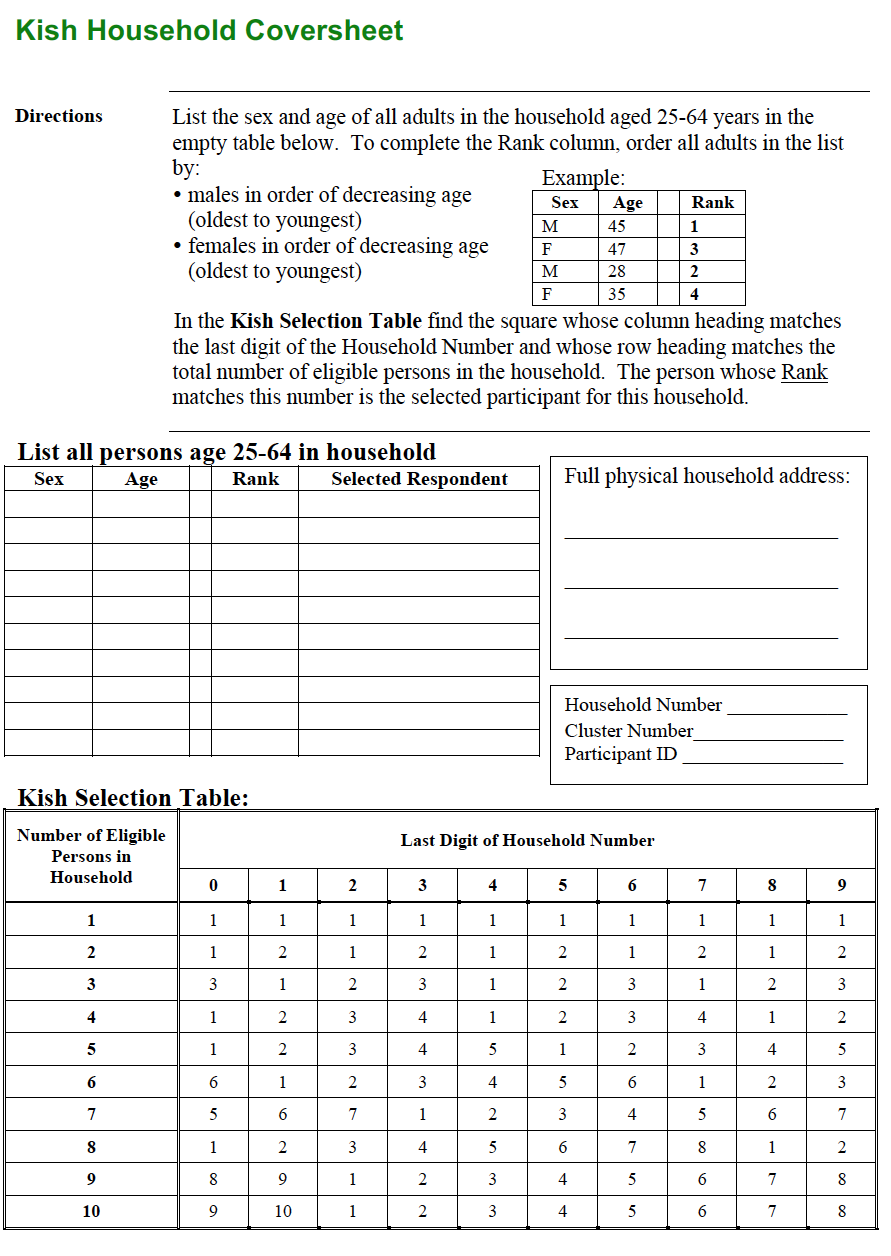
**

**
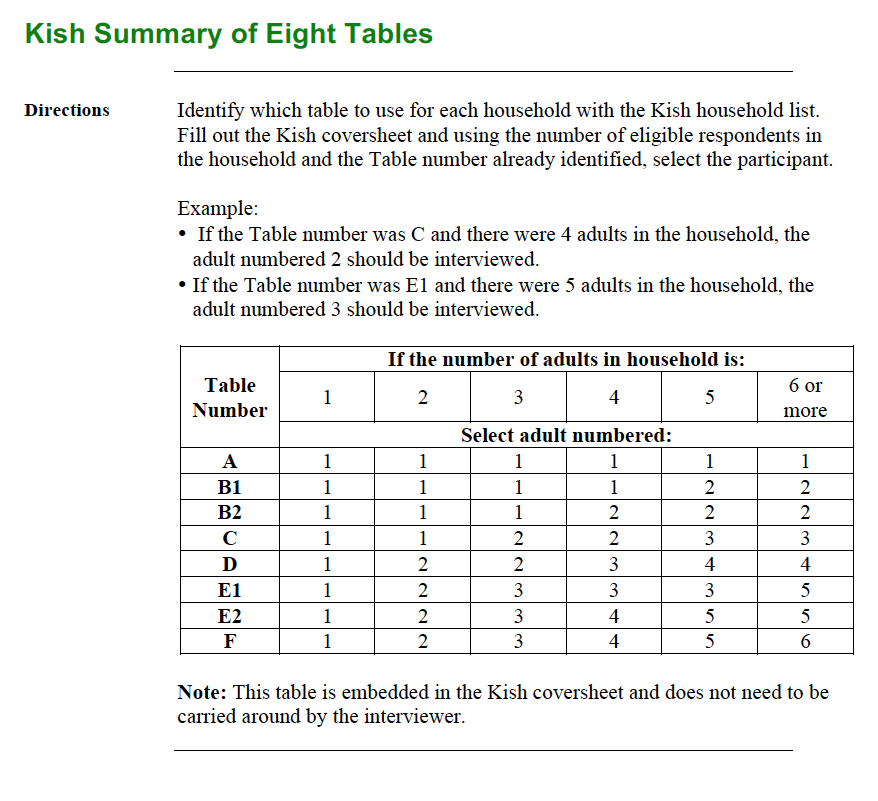
**

**SOURCE:** The appendix on KISH method, coversheet and summary of eight tables are taken from WHO STEPS documents and resources without modification.

APPENDIX 2: SHOW Cards

**SHOW CARDS: Serving sizes based on hand/fist**

| **SHOW CARD 1:**  1 Fist = 1 serving of:   - Whole fruit - Uncooked leafy veg - Tubers and high starch vegetables - Refined cereals and whole grain fibre-rich foods (excluding breads) | **SHOW CARD 2:**  Half Fist = 1 serving of:   - Small/cut fruit: fresh, frozen or canned - Small/cut vegetables: fresh, frozen or canned |
| --- | --- |
| 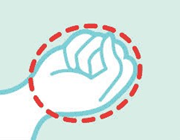  1 Fist = 1 cup = 250 ml | 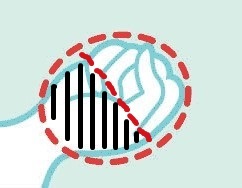  Half fist = ½ cup = 125ml |
| **SHOW CARD 3:**  Palm of hand = 1 serving of:   - 1 palm size slice of bread - 1 serving of fish | **SHOW CARD 4:**  Cupped hand = 1 serving of:   - Dried fruit |
| 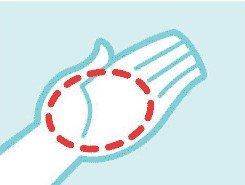  Palm of your hand | 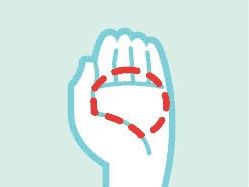  Cupped hand = ¼ cup = 60ml |

**SHOW CARD 5: Volume of Drinks**

| 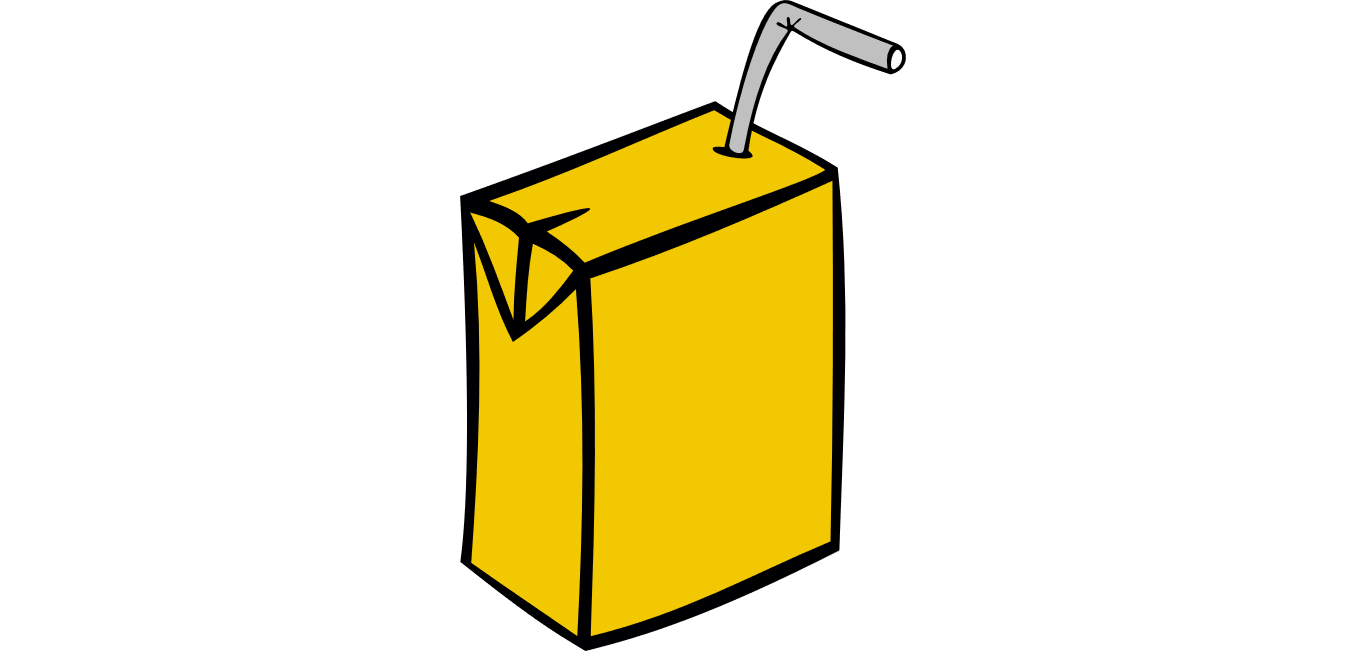 | 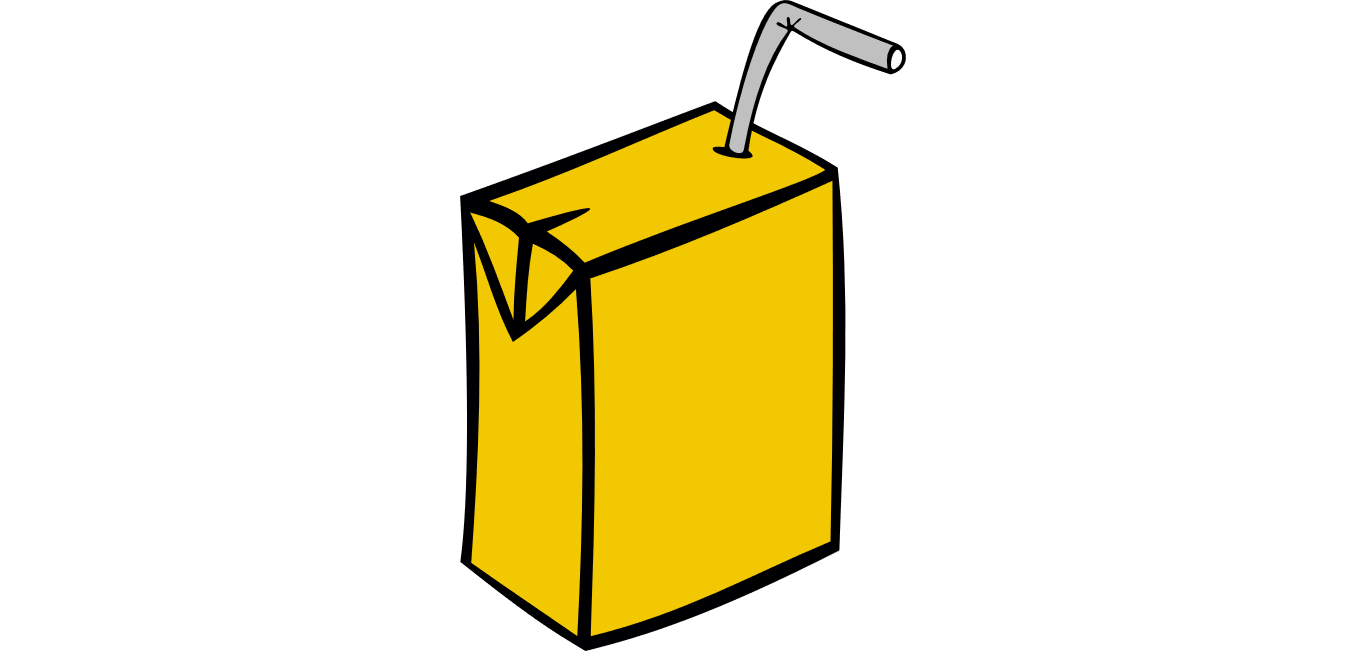 | 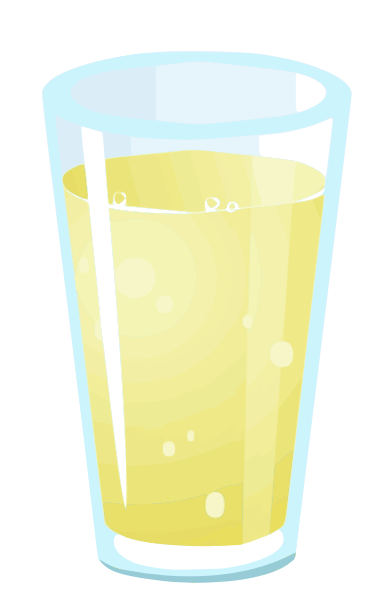 |
| --- | --- | --- |
| **5.1** Box drink 200ml | **5.2** Box drink 250ml | **5.3** Medium size cup 250ml |
| 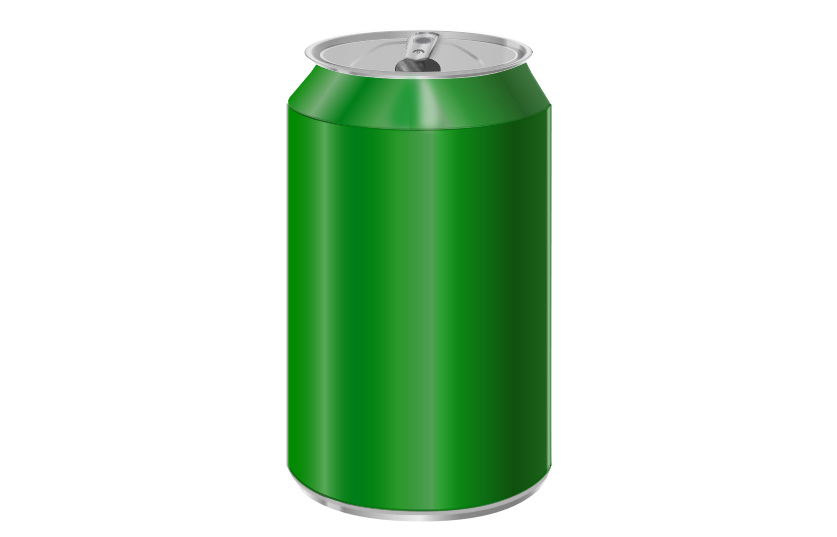  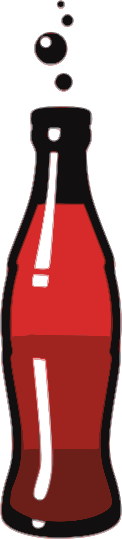 | 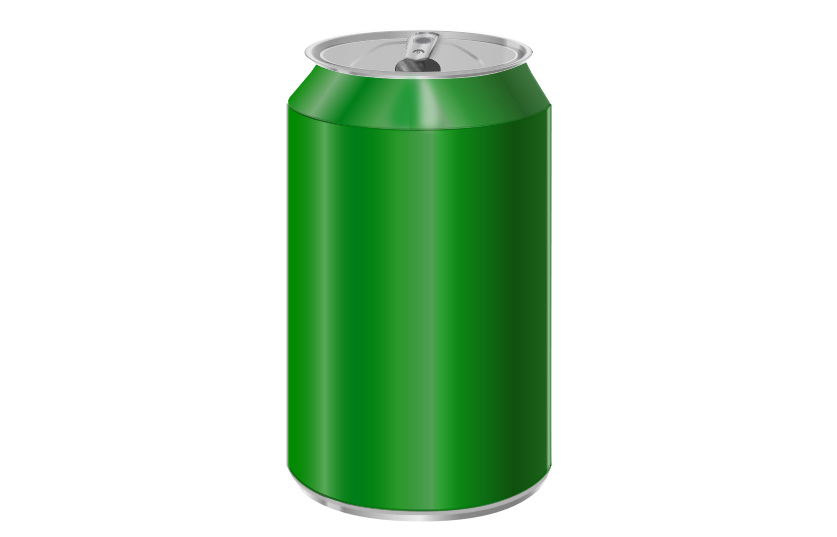  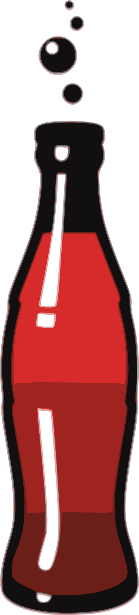 | 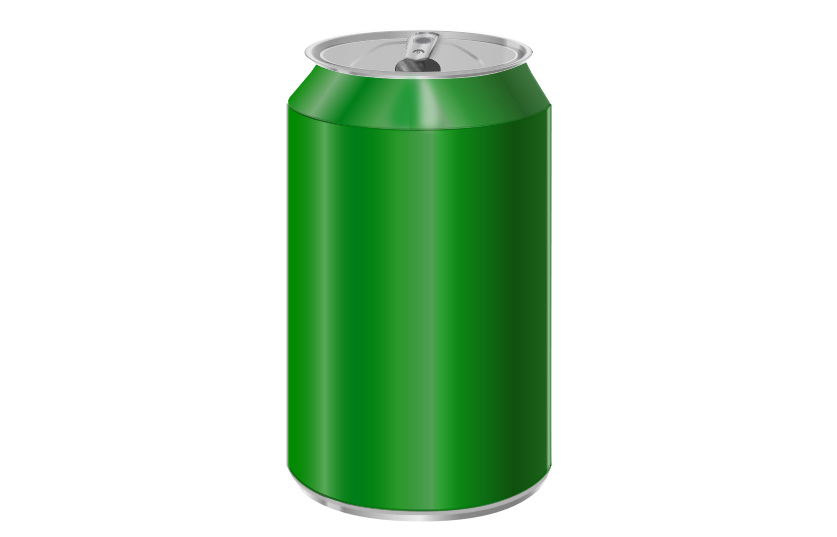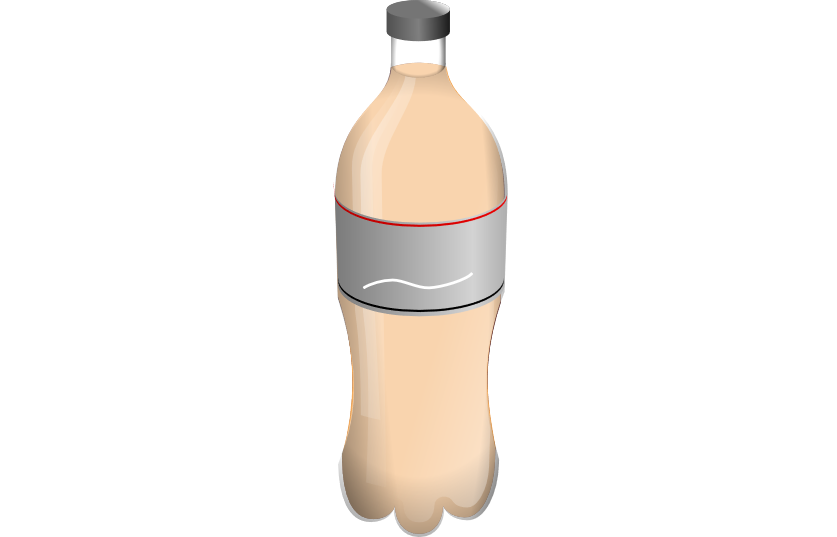 |
| **5.4** Regular can = smaller bottle = 300-330ml | **5.5** Medium can = medium bottle = 375ml | **5.6** Large can = large individual bottle = 500ml |

**SHOW CARD 6: Serving sizes for alcoholic drinks**

| 1 STANDARD DRINK = | |
| --- | --- |
| **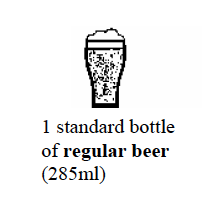** | **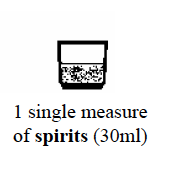** |
| **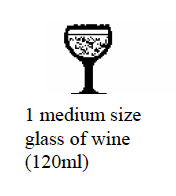** | **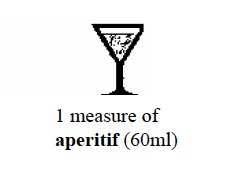** |
| **Note:** Net alcohol content of a **standard drink is approximately 10g** of ethanol. However, standard drinks in different countries can contain different amounts of ethanol. Please modify if this does not apply to your context. | |

**SHOW CARD 7: Responses (sections 13-18)**

**(Give the SHOWCARD to the participant so it will be easier for him/her to respond to the questions)**

| **1 Strongly disagree**  **2 Disagree**  **3 Neutral**  **4 Agree**  **5 Strongly agree**  **888 Not applicable** |
| --- |

1. In Health Facility and Community arm only [↑](#footnote-ref-1)
