# Supplementary material for: Prevention and management of type 2 diabetes mellitus in Uganda and South Africa: Findings from the SMART2D pragmatic implementation trial
Source: PLOS Glob Public Health. 2022 May 2;2(5):e0000425. doi: 10.1371/journal.pgph.0000425 (PMC10021626; doi:10.1371/journal.pgph.0000425)
Supplement: Attachment — Submitted filename: Response to Reviewers.docx [file pgph.0000425.s005.docx]

1^st^ March 2022.

The Editor,

PLOS Global Public Health.

Dear Sir/ Madam:

**Re:	PGPH-D-21-00724: Prevention and management of type 2 diabetes mellitus in Uganda and South Africa: Findings from the SMART2D pragmatic implementation trial**

Thank you very much for the review comments on our manuscript # PGPH-D-21-00724 titled “*Prevention and management of type 2 diabetes mellitus in Uganda and South Africa: Findings from the SMART2D pragmatic implementation trial*”; that we received on 26th January 2022. Attached to this letter is the list of a point-by-point response to the review comments. Where necessary, we have made changes in the manuscript as part of responding these comments.

I would like to mention that in the previous version of the manuscript, we had used the intention-to-treat analysis strategy in which missing observations on the study outcomes were treated as not have achieved the outcome of interest, and included in the analysis. Some of the comments by the reviewers prompted us to conduct a re-analysis of data on the two primary outcomes (glycemic control and reduction in HbA1c), using the multiple imputations strategy, which is currently a more robust and more acceptable technique to handle missing data. Given that multiple imputations of missing observations is currently known as the more robust technique to handle missing data, we have decided to replace the intention-to-treat analysis results with the multiple imputation analysis results (Table 3). But we are also submitting as supplementary information (S1_Table), the intention-to-treat analysis result in case readers may want to see these. We have included in the Discussion section of the manuscript, text discussing the two results. Important to note is that the retention into care findings that show significant and substantial difference between study-arms, have not change.

On behalf of my co-authors, I thank you for your consideration of our manuscript, and hope that you will find the revised version satisfactory for publication in your journal.

Yours Sincerely,

David Guwatudde, PhD.

Makerere University School of Public Health,

Kampala, Uganda.

| **#** | **Reviewer comment** | **Author response** |
| --- | --- | --- |
| **JOURNAL REQUIREMENTS** | | |
| 1. | Please ensure that the funders and grant numbers match between the Financial Disclosure field and the Funding Information tab in your submission form. Note that the funders must be provided in the same order in both places as well. Funding Information:  Funder Name: H2020 European Research Council  Grant Number: 643692  Grant Recipient: Meena Daivadanam  Funder Name: Agence Nationale de Recherches sur le Sida et les Hépatites Virales  Grant Number: HS 343  Grant Recipient: Prof David Guwatudde  Financial Disclosure:  This study was part of the SMART2D project funded by the European Commission's Horizon2020 Health Coordination Activities (Grant Agreement No 643692) under call “HCO-05-2014: Global Alliance for Chronic Diseases: prevention and treatment of type 2 diabetes”. The Uganda site was co-funded by the Sweden International Development Cooperation Agency (Sida) capacity-building grant to Makerere University 2015-2010, Project number HS 343. The contents of this article are solely the responsibility of the authors and do not reflect the views of the funders of the SMART2D Project. | This has been corrected. The uploaded funding source information now matches with that in the manuscript. The information is now as follows:  Funder Name: European Commission's Horizon2020 Health Coordination Activities  Grant Number: 643692  Grant Recipient: Meena Daivadanam  Funder Name: Swedish International Development Cooperation Agency (Sida)  Grant Number: HS 343  Grant Recipient: David Guwatudde, PhD  Financial Disclosure:  This study was part of the SMART2D project funded by the European Commission's Horizon2020 Health Coordination Activities (Grant Agreement No 643692) under call “HCO-05-2014: Global Alliance for Chronic Diseases: prevention and treatment of type 2 diabetes”. The Uganda site was co-funded by the Swedish International Development Cooperation Agency (Sida) capacity-building grant to Makerere University 2015-2010, Project #HS 343. The contents of this article are solely the responsibility of the authors and do not reflect the views of the funders of the SMART2D Project. |
| 2 | Please update your Competing Interests statement. If you have no competing interests to declare, please state:  “The authors have declared that no competing interests exist.” | This has been corrected in the uploaded information. |
| 3 | In the online submission form, you indicated that "The original study data is available from the corresponding author, and can be made available upon request and approval of co-investigators on:  David Guwatudde, Department of Epidemiology and Biostatistics,  School of Public Health, Makerere University College of Health Sciences, Kampala, Uganda. Email: dguwatudde@musph.ac.ug".  All PLOS journals now require all data underlying the findings described in their manuscript to be freely available to other researchers, either 1. In a public repository, 2. Within the manuscript itself, or 3. Uploaded as supplementary information. This policy applies to all data except where public deposition would breach compliance with the protocol approved by your research ethics board. If your data cannot be made publicly available for ethical or legal reasons (e.g., public availability would compromise patient privacy), please explain your reasons by return email and your exemption request will be escalated to the editor for approval. Your exemption request will be handled independently and will not hold up the peer review process, but will need to be resolved should your manuscript be accepted for publication. One of the Editorial team will then be in touch if there are any issues. | We have uploaded a de-identified data set of our study as supplementary information. |
| 4 | We have noticed that you have uploaded supporting information but you have not included a list of legends. Please add a full list of legends for all supporting information files (including figures, table and data files) after the references list. | We have provided a complete list of legends after the references list, for all the supporting information files that we have uploaded. |
| **REVIEWER #1** | | |
| 1 | General Comment: Diabetes Mellitus is a multisystem disease that is now a major public health concern worldwide. This is an interesting study and exciting to review. The manuscript is generally well written and structured, and will be a good read for researchers, clinicians, and the health promotion and disease prevention communities. Specific comments have been listed below. | No response required |
| 2 | Consider including a brief introduction or background in the abstract | We have improved the Abstract, and have included an opening introduction statement - see lines 37 – 42 of the clean copy. |
| 3 | Line 39: The design is not reproducible. Clarity with precision will be required. | Also based on other comments, we have improved the wording in the abstract, and hope that the improved wording in regards to the study design makes it clearer - see lines 43 – 45 of the clean copy. Given the word count limit for the abstract, it is not possible to be more explicit in the abstract. The reader has to go further into the paper, i.e. the Method section, to see details about the design – see lines 113 – 124.. |
| 4 | Line 52: Be specific, indicate p-values | The statement the reviewer is referring to was in regard to adjusted analysis. We have improved the statement for clarity - see lines 51 – 55 of the clean copy. |
| 5 | Line 54: indicate p-values | Similar to the preceding response, we have provided the incident rate ratios including 95% confidence intervals from our adjusted analysis. We believe these are sufficient to give the reader the level of precision. |
| 6 | Line 65: Full stop to be placed after the reference citation, not before: example - "….especially in low- and middle income counties (LMICs)"[1]. Consider this in entire text. | This has been corrected throughout the manuscript. |
| 7 | Line 68, comma after "2019" | This has been done – see line 69 of the clean copy |
| 8 | Lines 74-76, "..18.1% were treated but uncontrolled, and only 19.4% were treated and controlled; suggesting that 80.6% of the population with diabetes had unmet need for care". Consult your reference and reword for simplicity. | We made an error when summarizing the unmet need for care reported in this article. This has been corrected – see lines 74 – 78 of the clean copy. |
|  | Clinical definition of Type 2 DM should be stated in your introduction or outcome section | We provided the definition, or the inclusion criteria for the participants with T2D that we enrolled into the trial. The inclusion criteria now appears in lines 141 - 145, of the clean copy. |
| 9 | Line 127, comma after criteria | The comma has been added – see line 120 of the clean copy. |
| 10 | Line 187: How long was the intervention? | We have added a sentence in the “Interventions” section, stating that “Participants were exposed to the interventions for at least 9 months”. See lines 168 - 169 in the clean copy. |
| 11 | Line 223: since this is a pre-post intervention study, was there a consideration to evaluate the possible effect of the intervention using the dependent/paired t- test? | Our study did not use a “pre-post intervention study design”. The study had two concurrent/parallel study arms in South Africa, and three concurrent/parallel study arms in Uganda. We believe this is clear as described in the “Study Design and setting” sub-section” – see lines 113 - 134 of the tracked copy |
| 12 | Line 253: check spelling - baseline | The spelling mistake has been corrected. See line 278 of the tracked copy. |
| 13 | Table 1: The break-off to new page must capture the headings as in previous page. | This has been corrected – see Table 1. |
| 14 | Line 323: Significance should be relative to p-value and not percentage in each country. It is vital to clearly indicate how the post intervention characteristics differed from the baseline characteristic in each country/study area using an appropriate statistical test. | In addition to the percentages in each country that the reviewer is referring to (previously lines 323 -324), we also did provide the p-value at the end of the sentence in question. This now appears in lines 348- 349 of the clean copy. |
| 15 | Table 3: "0.00128¥" Should this be here in this column or the next column? If this is the p-value, check if this contradicts the statement in lines 324-325. | This is the inter-cluster correlation coefficient value, it is not a p-value. For clarity, we have now included the standard error (SE) for each of the ICC values, in Table 4. |
| 16 | Table 3": write in full. Avoid using "#" as headings | This has been corrected in all the tables |
| 17 | Table 3: place the p-values for the ICC at the appropriate columns dedicated for p-values | These are the inter-cluster correlation coefficient values, they are not p-values. For clarity, we have included the standard error (SE) values against each ICC value – Table 4. |
| 18 | Table 6: regarding the Adjusted IRR, were there measures in place to control confounding factors? | Yes, we controlled for confounding during adjusted analysis, and for each final model the factors we controlled for, are listed in the footnotes of each Table 3 and 4. |
| 19 | Table 6: Any explanation why there is no 'usual care' in South Africa? | We had provided an explanation for this but we realize this may not have been clear. We have improved the wording in the sub-section on “Study design and setting”, lines 113 – 124 of the clean copy, which provides the explanation. |
| 20 | Line 353, hba1c to be written appropriately - HbA1c | This has been corrected – see line 356 of the clean copy. |
| 21 | Line 399: Was there any confounding factor(s) that may have affected the percentage outcome based on the crude IRR and adjusted models? | Yes and although we do not list these, we provided both the crude and adjusted IRRs so that readers can see how some of the crude IRRs changed after adjusting for confounding. The factors adjusted for, during multivariable analysis are listed in the footnotes of each of the Tables 3 and 4. |
| 22 | Lines 406-407: "….rates of retention into were significantly higher in…" Seems incomplete; possible omission.  Read again and revise accordingly. | The rates reported here are in comparison to the Usual care arm. A comma was missing in the sentence “*Adjusted analysis showed that in Uganda compared to the usual care arm, rates of retention into were significantly higher in the facility care arm with an IRR= 2.46 [1.33 – 4.43], and in integrated care arm with an IRR= 3.52 [2.13 – 5.80]*”; which made the sentence appear like there was something missing.  The comma has been added between the words Uganda and compared to read: “*Adjusted analysis showed that in Uganda, compared to the usual care arm, rates of retention into were significantly higher in the facility care arm with an IRR= 2.46 [1.33 – 4.43], and in integrated care arm with an IRR= 3.52 [2.13 – 5.80]*”; which now makes the sentence complete. See lines 390-394 of the clean copy. |
| 23 | Line 411: Cited table in text should be in circle brackets. | This has been done. See line 394 of the clean copy. |
| **REVIEWER #2** | | |
| 1 | This is an important addition to literature. It is novel and topical. I strongly support its publication. However, in its present form, it is really difficult to understand the study design, who the patients were (e.g. how selected) and therefore what do the findings mean. | Regarding the study design, we have improved the description in the “Study design and site” sub-section, and we believe that this now makes it clearer. See lines 113 – 124 of the clean copy. |
| 2 | The abstract lacks details and is impossible to understand. What are the 3 arms, what is a high risk patient and what is the difference between glycaemic control and HbA1c control? I don't understand the rate ratios in the abstract. | We have improved the Abstract, and we hope the current version is clearer. |
| 3 | Even in the methods, it is unclear what are the different arms? What is the difference between facility care and usual care. The latter is not described. Perhaps the authors should consider a Table / chart to describe these side by side so that the reader can see the difference. Also think of better names for these arms that bring great clarity. | The intervention elements in each of the study arms are described in the “Interventions” sub-section, lines 157 – 172 of the clean copy. Further, details of each these intervention strategies and elements are described, in fact in a tabular form, in our already published protocol article which we reference in line 168. We did not find it necessary to reproduce these details in this manuscript, since these details are already published. |
| 4 | In the outcomes section, please explain what does 3 mmol/mol mean clinically. | The cutoff reduction of 3 mmol/mol by month 12 among participants at high risk of T2D was based on findings from intervention study reported by Katula et al (2021) that aimed at reducing HbA1c levels among people with pre-diabetes. In their study, investigators observed a significant reduction in HbA1c level, an average of -2.52 mmol/mol [95% CI= -2.89 – -2.16]. Based on this, we decided to use a cutoff reduction of 3 mmol/mol by month 12. We have added text to clarify the basis of this cutoff – see lines 220 - 226. |
| 5 | The study design is not clear. Is this a randomised trial, with randomisation done by clusters? And how were participants selected from the pool of people with diabetes or at high risk. | Yes indeed the randomization was done by clusters. We have improved the description in the “Study design and site” sub-section (see lines 113 – 124), and we believe that this now makes it clearer. |
| 6 | The results section is very difficult to follow with too many comparisons and too many outcomes. The authors really must focus on their primary questions, which I guess are the effects of the different models of care. I can't work out whether the analyses in T4 tally with those in T3. These 2 tables should really be combined into one table. | We have merged previously Table 3 and previously Table 4,into now Table 3. |
| 7 | Why is there such a powerful effect of integrated care among high-risk but not among people with T2D on glycaemic indicators. Does seem a bit odd? | We thank the reviewer for this observation. In the previous version of the manuscript, we had used the intention-to-treat analysis strategy in which missing observations on the study outcomes were treated as not have achieved the outcome of interest, and included in the analysis.  However this comment prompted us to conduct a re-analysis of data on the two primary outcomes (glycemic control and reduction in HbA1c), using the multiple imputations strategy, which is currently a more robust and more acceptable technique to handle missing data. Given that multiple imputations of missing observations is currently known as the more robust technique to handle missing data, we have decided to replace the intention-to-treat analysis results with the multiple imputation analysis results (Table 3). Multiple imputation analysis does not reveal any significant differences between the study arms in regards to glycemic control or reduction in HbA1c.  But we are also submitting as supplementary information (S1_Table), the intention-to-treat analysis results in case readers may want to see these. We have included in the Discussion section of the manuscript, text discussing the two results. Important to note is that findings on retention into care that were significant and substantial difference between study-arms, have not changed. |
| 8 | Likewise, T5 and T6 ought to be combined into one table. | We have merged the previously Tables 5 and 6, into onto one table, now Table 4. |
